# Evidence that the 5p12 Variant rs10941679 Confers Susceptibility to Estrogen-Receptor-Positive Breast Cancer through *FGF10* and *MRPS30* Regulation

**DOI:** 10.1016/j.ajhg.2016.07.017

**Published:** 2016-09-15

**Authors:** Maya Ghoussaini, Juliet D. French, Kyriaki Michailidou, Silje Nord, Jonathan Beesley, Sander Canisus, Kristine M. Hillman, Susanne Kaufmann, Haran Sivakumaran, Mahdi Moradi Marjaneh, Jason S. Lee, Joe Dennis, Manjeet K. Bolla, Qin Wang, Ed Dicks, Roger L. Milne, John L. Hopper, Melissa C. Southey, Marjanka K. Schmidt, Annegien Broeks, Kenneth Muir, Artitaya Lophatananon, Peter A. Fasching, Matthias W. Beckmann, Olivia Fletcher, Nichola Johnson, Elinor J. Sawyer, Ian Tomlinson, Barbara Burwinkel, Frederik Marme, Pascal Guénel, Thérèse Truong, Stig E. Bojesen, Henrik Flyger, Javier Benitez, Anna González-Neira, M. Rosario Alonso, Guillermo Pita, Susan L. Neuhausen, Hoda Anton-Culver, Hermann Brenner, Volker Arndt, Alfons Meindl, Rita K. Schmutzler, Hiltrud Brauch, Ute Hamann, Daniel C. Tessier, Daniel Vincent, Heli Nevanlinna, Sofia Khan, Keitaro Matsuo, Hidemi Ito, Thilo Dörk, Natalia V. Bogdanova, Annika Lindblom, Sara Margolin, Arto Mannermaa, Veli-Matti Kosma, kConFab/AOCS Investigators, Anna H. Wu, David Van Den Berg, Diether Lambrechts, Giuseppe Floris, Jenny Chang-Claude, Anja Rudolph, Paolo Radice, Monica Barile, Fergus J. Couch, Emily Hallberg, Graham G. Giles, Christopher A. Haiman, Loic Le Marchand, Mark S. Goldberg, Soo H. Teo, Cheng Har Yip, Anne-Lise Borresen-Dale, NBCS Collaborators, Wei Zheng, Qiuyin Cai, Robert Winqvist, Katri Pylkäs, Irene L. Andrulis, Peter Devilee, Rob A.E.M. Tollenaar, Montserrat García-Closas, Jonine Figueroa, Per Hall, Kamila Czene, Judith S. Brand, Hatef Darabi, Mikael Eriksson, Maartje J. Hooning, Linetta B. Koppert, Jingmei Li, Xiao-Ou Shu, Ying Zheng, Angela Cox, Simon S. Cross, Mitul Shah, Valerie Rhenius, Ji-Yeob Choi, Daehee Kang, Mikael Hartman, Kee Seng Chia, Maria Kabisch, Diana Torres, Craig Luccarini, Don M. Conroy, Anna Jakubowska, Jan Lubinski, Suleeporn Sangrajrang, Paul Brennan, Curtis Olswold, Susan Slager, Chen-Yang Shen, Ming-Feng Hou, Anthony Swerdlow, Minouk J. Schoemaker, Jacques Simard, Paul D.P. Pharoah, Vessela Kristensen, Georgia Chenevix-Trench, Douglas F. Easton, Alison M. Dunning, Stacey L. Edwards

**Affiliations:** 1 Centre for Cancer Genetic Epidemiology, Department of Oncology, University of Cambridge, Cambridge CB1 8RN, UK; 2 Cancer Division, QIMR Berghofer Medical Research Institute, Brisbane, QLD 4006, Australia; 3 Centre for Cancer Genetic Epidemiology, Department of Public Health and Primary Care, University of Cambridge, Cambridge CB1 8RN, UK; 4 Department of Electron Microscopy/Molecular Pathology, The Cyprus Institute of Neurology and Genetics, Nicosia 1683, Cyprus; 5 Department of Cancer Genetics, Institute for Cancer Research, Oslo University Hospital Radiumhospitalet, 0310 Oslo, Norway; 6 Netherlands Cancer Institute, Antoni van Leeuwenhoek Hospital, 1066 CX Amsterdam, the Netherlands; 7 Cancer Epidemiology Centre, Cancer Council Victoria, Melbourne, VIC 3004, Australia; 8 Centre for Epidemiology and Biostatistics, Melbourne School of Population and Global health, The University of Melbourne, Melbourne, VIC 3010, Australia; 9 Department of Pathology, The University of Melbourne, Melbourne, VIC 3010, Australia; 10 Institute of Population Health, University of Manchester, Manchester M13 9PL, UK; 11 Division of Health Sciences, Warwick Medical School, Warwick University, Coventry CV4 7AL, UK; 12 Department of Gynaecology and Obstetrics, University Hospital Erlangen, Friedrich-Alexander University Erlangen-Nuremberg, Comprehensive Cancer Center Erlangen-EMN, 91054 Erlangen, Germany; 13 David Geffen School of Medicine, Department of Medicine Division of Hematology and Oncology, University of California at Los Angeles, Los Angeles, CA 90095, USA; 14 Toby Robins Breast Cancer Now Research Centre, The Institute of Cancer Research, London SW3 6JB, UK; 15 Division of Breast Cancer Research, The Institute of Cancer Research, London SW7 3RP, UK; 16 Research Oncology, Guy’s Hospital, King’s College London, London SE1 9RT, UK; 17 Wellcome Trust Centre for Human Genetics and Oxford NIHR Biomedical Research Centre, University of Oxford, Oxford OX3 7BN, UK; 18 Department of Obstetrics and Gynecology, University of Heidelberg, 69120 Heidelberg, Germany; 19 Molecular Epidemiology Group, German Cancer Research Center (DKFZ), 69120 Heidelberg, Germany; 20 National Center for Tumor Diseases, University of Heidelberg, 69120 Heidelberg, Germany; 21 Cancer & Environment Group, Center for Research in Epidemiology and Population Health (CESP), INSERM, University Paris-Sud, University Paris-Saclay, 94807 Villejuif, France; 22 Copenhagen General Population Study, Herlev and Gentofte Hospital, Copenhagen University Hospital, 2730 Herlev, Denmark; 23 Department of Clinical Biochemistry, Herlev and Gentofte Hospital, Copenhagen University Hospital, 2730 Herlev, Denmark; 24 Faculty of Health and Medical Sciences, University of Copenhagen, 2200 Copenhagen, Denmark; 25 Department of Breast Surgery, Herlev and Gentofte Hospital, Copenhagen University Hospital, 2730 Herlev, Denmark; 26 Human Cancer Genetics Program, Spanish National Cancer Research Centre, 28029 Madrid, Spain; 27 Centro de Investigación en Red de Enfermedades Raras, 46010 Valencia, Spain; 28 Human Genotyping-CEGEN Unit, Human Cancer Genetic Program, Spanish National Cancer Research Centre, 28029 Madrid, Spain; 29 Department of Population Sciences, Beckman Research Institute of City of Hope, Duarte, CA 92697, USA; 30 Department of Epidemiology, University of California Irvine, Irvine, CA 92697, USA; 31 Division of Clinical Epidemiology and Aging Research, German Cancer Research Center (DKFZ), 69120 Heidelberg, Germany; 32 German Cancer Consortium (DKTK), German Cancer Research Center (DKFZ), 69120 Heidelberg, Germany; 33 Division of Preventive Oncology, German Cancer Research Center (DKFZ) and National Center for Tumor Diseases (NCT), 69120 Heidelberg, Germany; 34 Division of Gynaecology and Obstetrics, Technische Universität München, 81675 Munich, Germany; 35 Center for Hereditary Breast and Ovarian Cancer, University Hospital of Cologne, 50931 Cologne, Germany; 36 Center for Integrated Oncology (CIO), University Hospital of Cologne, 50937 Cologne, Germany; 37 Center for Molecular Medicine Cologne (CMMC), University of Cologne, 50931 Cologne, Germany; 38 Dr. Margarete Fischer-Bosch-Institute of Clinical Pharmacology, 70376 Stuttgart, Germany; 39 University of Tübingen, 72074 Tübingen, Germany; 40 Molecular Genetics of Breast Cancer, German Cancer Research Center (DKFZ), 69120 Heidelberg, Germany; 41 McGill University and Génome Québec Innovation Centre, Montréal, QC H3A OG1, Canada; 42 Department of Obstetrics and Gynecology, Helsinki University Hospital, University of Helsinki, 00029 Helsinki, Finland; 43 Division of Molecular Medicine, Aichi Cancer Center Research Institute, Nagoya 464-8681, Japan; 44 Division of Epidemiology and Prevention, Aichi Cancer Center Research Institute, Nagoya 464-8681, Japan; 45 Gynaecology Research Unit, Hannover Medical School, 30625 Hannover, Germany; 46 Department of Radiation Oncology, Hannover Medical School, 30625 Hannover, Germany; 47 Department of Molecular Medicine and Surgery, Karolinska Institutet, 17177 Stockholm, Sweden; 48 Department of Oncology-Pathology, Karolinska Institutet, 17177 Stockholm, Sweden; 49 Cancer Center of Eastern Finland, University of Eastern Finland, 70211 Kuopio, Finland; 50 Institute of Clinical Medicine, Pathology and Forensic Medicine, University of Eastern Finland, 70211 Kuopio, Finland; 51 Imaging Center, Department of Clinical Pathology, Kuopio University Hospital, 70210 Kuopio, Finland; 52 Department of Preventive Medicine, Keck School of Medicine, University of Southern California, Los Angeles, CA 90033, USA; 53 Vesalius Research Center, VIB, 3000 Leuven, Belgium; 54 Laboratory for Translational Genetics, Department of Oncology, University of Leuven, 3000 Leuven, Belgium; 55 University Hospital Gashuisberg, 3000 Leuven, Belgium; 56 Division of Cancer Epidemiology, German Cancer Research Center (DKFZ), 69120 Heidelberg, Germany; 57 University Cancer Center Hamburg (UCCH), University Medical Center Hamburg-Eppendorf, 20246 Hamburg, Germany; 58 Unit of Molecular Bases of Genetic Risk and Genetic Testing, Department of Preventive and Predictive Medicine, Fondazione IRCCS (Istituto Di Ricovero e Cura a Carattere Scientifico) Istituto Nazionale dei Tumori (INT), 20133 Milan, Italy; 59 Division of Cancer Prevention and Genetics, Istituto Europeo di Oncologia, 20141 Milan, Italy; 60 Department of Laboratory Medicine and Pathology, Mayo Clinic, Rochester, MN 55905, USA; 61 Department of Health Sciences Research, Mayo Clinic, Rochester, MN 55905, USA; 62 University of Hawaii Cancer Center, Honolulu, HI 96813, USA; 63 Department of Medicine, McGill University, Montreal, QC H3G 2M1, Canada; 64 Division of Clinical Epidemiology, Royal Victoria Hospital, McGill University, Montreal, QC H3A 1A8, Canada; 65 Cancer Research Initiatives Foundation, Subang Jaya, 47500 Selangor, Malaysia; 66 Breast Cancer Research Unit, Cancer Research Institute, University Malaya Medical Centre, 59100 Kuala Lumpur, Malaysia; 67 Division of Epidemiology, Department of Medicine, Vanderbilt-Ingram Cancer Center, Vanderbilt University School of Medicine, Nashville, TN 37203, USA; 68 Laboratory of Cancer Genetics and Tumor Biology, Cancer Research and Translational Medicine, Biocenter Oulu, University of Oulu, 90220 Oulu, Finland; 69 Laboratory of Cancer Genetics and Tumor Biology, Northern Finland Laboratory Centre Oulu, 90220 Oulu, Finland; 70 Lunenfeld-Tanenbaum Research Institute of Mount Sinai Hospital, Toronto, ON M5G 1X5, Canada; 71 Department of Molecular Genetics, University of Toronto, Toronto, ON M5S 1A8, Canada; 72 Department of Pathology, Leiden University Medical Center, 2300 RC Leiden, the Netherlands; 73 Department of Human Genetics, Leiden University Medical Center, 2300 RC Leiden, the Netherlands; 74 Department of Surgery, Leiden University Medical Center, 2300 RC Leiden, the Netherlands; 75 Division of Cancer Epidemiology and Genetics, National Cancer Institute, Rockville, MD 20850, USA; 76 Usher Institute of Population Health Sciences and Informatics, The University of Edinburgh Medical School, Edinburgh EH8 9AG, UK; 77 Department of Medical Epidemiology and Biostatistics, Karolinska Institutet, 17177 Stockholm, Sweden; 78 Department of Medical Oncology, Family Cancer Clinic, Erasmus MC Cancer Institute, 3008 AE Rotterdam, the Netherlands; 79 Department of Surgical Oncology, Family Cancer Clinic, Erasmus MC Cancer Institute, 3008 AE Rotterdam, the Netherlands; 80 Shanghai Municipal Center for Disease Control and Prevention, 200336 Shanghai, China; 81 Sheffield Cancer Research, Department of Oncology and Metabolism, University of Sheffield, Sheffield S10 2RX, UK; 82 Academic Unit of Pathology, Department of Neuroscience, University of Sheffield, Sheffield S10 2HQ, UK; 83 Department of Biomedical Sciences, Seoul National University College of Medicine, Seoul 110-799, Korea; 84 Cancer Research Institute, Seoul National University, Seoul 110-799, Korea; 85 Department of Preventive Medicine, Seoul National University College of Medicine, Seoul 110-799, Korea; 86 Saw Swee Hock School of Public Health, National University of Singapore, Singapore 117597, Singapore; 87 Department of Surgery, National University Health System, Singapore 117597, Singapore; 88 Institute of Human Genetics, Pontificia Universidad Javeriana, Bogota, DC 11001000, Colombia; 89 Department of Genetics and Pathology, Pomeranian Medical University, 70-115 Szczecin, Poland; 90 National Cancer Institute, Bangkok 10400, Thailand; 91 International Agency for Research on Cancer, Lyon Cedex 08, France; 92 School of Public Health, China Medical University, Taichung 40402, Taiwan; 93 Taiwan Biobank, Institute of Biomedical Sciences, Academia Sinica, Taipei 115, Taiwan; 94 Department of Surgery, Kaohsiung Municipal Hsiao-Kang Hospital, Kaohsiung 812, Taiwan; 95 Division of Genetics and Epidemiology, The Institute of Cancer Research, London SM2 5NG, UK; 96 Genomics Center, Centre Hospitalier Universitaire de Québec Research Center, Laval University, Québec City, QC G1V 4G2, Canada; 97 Department of Clinical Molecular Biology, Oslo University Hospital, University of Oslo, 0450 Oslo, Norway

## Abstract

Genome-wide association studies (GWASs) have revealed increased breast cancer risk associated with multiple genetic variants at 5p12. Here, we report the fine mapping of this locus using data from 104,660 subjects from 50 case-control studies in the Breast Cancer Association Consortium (BCAC). With data for 3,365 genotyped and imputed SNPs across a 1 Mb region (positions 44,394,495–45,364,167; NCBI build 37), we found evidence for at least three independent signals: the strongest signal, consisting of a single SNP rs10941679, was associated with risk of estrogen-receptor-positive (ER^+^) breast cancer (per-*g* allele OR ER^+^ = 1.15; 95% CI 1.13–1.18; p = 8.35 × 10^−30^). After adjustment for rs10941679, we detected signal 2, consisting of 38 SNPs more strongly associated with ER-negative (ER^−^) breast cancer (lead SNP rs6864776: per-*a* allele OR ER^−^ = 1.10; 95% CI 1.05–1.14; p conditional = 1.44 × 10^−12^), and a single signal 3 SNP (rs200229088: per-*t* allele OR ER^+^ = 1.12; 95% CI 1.09–1.15; p conditional *=* 1.12 × 10^−05^). Expression quantitative trait locus analysis in normal breast tissues and breast tumors showed that the *g* (risk) allele of rs10941679 was associated with increased expression of *FGF10* and *MRPS30*. Functional assays demonstrated that SNP rs10941679 maps to an enhancer element that physically interacts with the *FGF10* and *MRPS30* promoter regions in breast cancer cell lines. FGF10 is an oncogene that binds to FGFR2 and is overexpressed in ∼10% of human breast cancers, whereas *MRPS30* plays a key role in apoptosis. These data suggest that the strongest signal of association at 5p12 is mediated through coordinated activation of *FGF10* and *MRPS30*, two candidate genes for breast cancer pathogenesis.

## Main Text

Strong evidence for the existence of a breast cancer (MIM: 114480) susceptibility locus at 5p12 has been observed through a GWAS in Iceland (SNP rs7703618),^1^ in the Breast Cancer Association Consortium (BCAC; SNP rs981782, 371 Kb centromeric),^2^ and in the Cancer GEnetic Markers of Susceptibility study (CGEMS; SNP rs4866929; 352 Kb centromeric; *r*^2^ = 0.18).^3^ A subsequent study, using 22 SNPs in ∼5,000 case subjects and ∼33,000 control subjects of European ancestry, reported that risk at this locus could be explained by two SNPs: rs4415084 and rs10941679.^4^ More recently, a BCAC study confirmed that rs10941679 was associated with risk of lower-grade, progesterone receptor (*PGR* [MIM: 607311])-positive breast cancer tumors.^5^

Here, we report the comprehensive fine-scale mapping of this locus in 104,660 subjects from 50 case-control studies participating in BCAC, including 41 studies from populations of European ancestry and nine of East Asian ancestry, and we explore the functional mechanisms underlying the associations in this region. Genotyping was conducted with the COGS array, a custom array comprising approximately 200,000 SNPs.^6^ After quality-control exclusions, we analyzed data from 48,155 case subjects and 43,612 control subjects of European ancestry and 6,269 case subjects and 6,624 control subjects of Asian ancestry. Estrogen receptor (*ESR1* [MIM: 133430]) status of the primary tumor was available for 27,748 European and 4,997 Asian case subjects; of these, 7,646 (22%) European and 1,623 (32%) Asian case subjects were ER^−^.

We examined a 1 Mb region (positions 44,394,495–45,364,167; NCBI build 37 assembly) in which the 1000 Genomes Project cataloged 1,811 variants (March 2010 Pilot version 60 CEU project data). We aimed to genotype all 628 SNPs with minor allele frequency (MAF) > 2% and correlated with rs981782 and rs10941679 at *r*^2^ > 0.1 (n = 424), plus a set of SNPs designed to tag all remaining SNPs with *r*^2^ > 0.9 (n = 184), but we managed to include 563 SNPs with a designability score (DS) > 0.9 and which passed QC.^6^ IMPUTE v.2.0 was used to impute genotypes of all known SNPs in the region using the 1000 Genome Project data (March 2012 version) as a reference panel.

**Figure 1:**
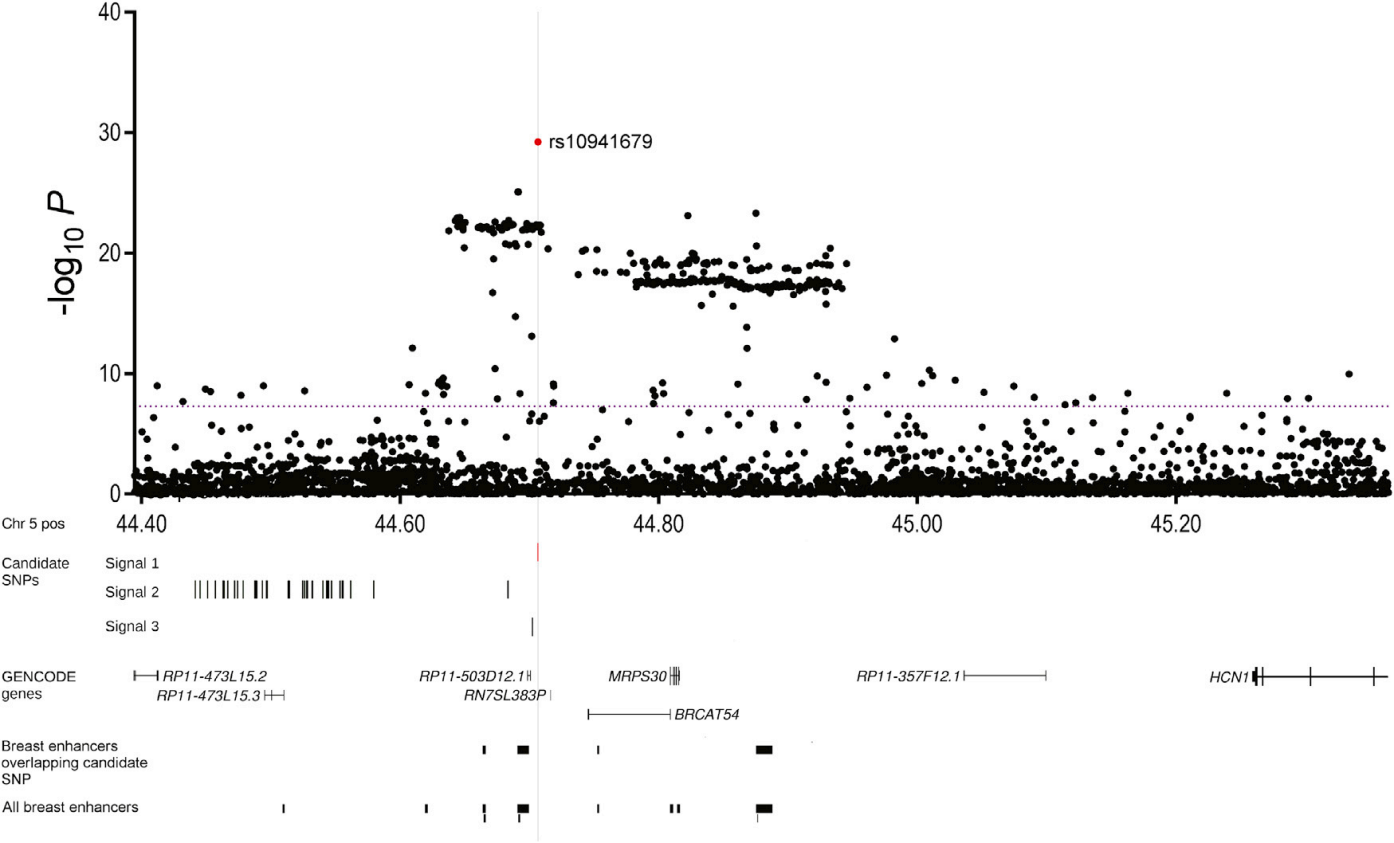
Manhattan Plot of the 5p12 Breast Cancer Susceptibility Locus SNPs are plotted according to their chromosomal position on the x axis and their overall p values (log_10_ values, likelihood ratio test) from the European BCAC studies (48,155 case and 43,612 control subjects) on the y axis. The purple dotted line intersects the y axis at p = 10^−8^ and indicates genome-wide significance. Candidate SNPs in signal 1 (rs10941679), signal 2 (38 SNPs), and signal 3 (rs200229088) are shown as short vertical lines. The locations of annotated genes and putative lncRNA transcripts from GENCODE and enhancers predicted in Corradin et al.^13^ and Hnisz et al.^12^ from breast cancer cell lines are shown in the bottom panels.

Case-control analyses were conducted on 3,365 SNPs (563 genotyped and 2,776 imputed at *r*^2^ > 0.3). In European-ancestry women, 461 of these SNPs were associated with overall breast cancer risk, 489 with ER^+^ and 38 with ER^−^ breast cancer risk (p < 10^−4^; Table S1). SNP rs10941679 showed the strongest overall association (MAF = 0.27, per-minor (*g*) allele: OR = 1.12; 95% CI 1.10–1.14; p = 2.55 × 10^−26^; Figure 1, Tables 1 and S1). To identify additional association signals at this region, we conducted a forward stepwise logistic regression examining SNPs with univariate p < 0.1 (n = 1,040).^6^ The most parsimonious model included three variants: SNP1 rs10941679 (signal 1), SNP2 rs6864776 (signal 2; conditional p = 6.22 × 10^−11^), and SNP3 rs200229088 (signal 3; conditional p = 1.12 × 10^−5^, borderline significance; Table S2). SNP1 and SNP3 are weakly correlated (*r*^2^ = 0.15) but SNP2 was uncorrelated with the other two (*r*^2^ = 0.07 and 0.05).

The top signal, SNP1 rs10941679, is markedly more significant than any other SNP in the locus (likelihood ratio > 10,000:1). Hence, the most parsimonious explanation is that this SNP is causally related to risk. The next most strongly associated SNP, after adjustment for signal 1 SNP rs10941679, was rs6864776, representing signal 2 (OR per minor allele = 1.04; 95% CI 1.02–1.06; p = 7.84 × 10^−4^; conditional p = 1.44 × 10^−12^). Within signal 2, a further 37 SNPs correlated with rs6864776 at *r*^2^ > 0.6, had likelihood ratios of <100:1 relative to rs6864776, and hence could not be excluded from being causative statistically (Table S2). After adjustment for both signal 1 SNP rs10941679 and signal 2 top SNP rs6864776, a single SNP remained: rs200229088 (OR overall = 1.09, 95%; CI 1.07–1.12; p = 2.28 × 10^−12^; conditional p = 1.12 × 10^−5^). There are no other SNPs correlated with rs200229088 that could explain this association. All other SNPs were excluded from causality (likelihood ratio > 10,000:1; Table S2). Two of the excluded variants had been previously postulated as likely causative variants^4^, ^7^ and so we investigated these in more depth. We found both SNPs to be partially correlated with all three signals and consequently display initially inflated effects, which are adjusted by the conditional analyses. Thus, SNP rs4415084^4^ (*r*^2^ with signal 1 SNP rs10941679 = 0.51, with signal 2 SNP rs6864776 = 0.11, and with signal 3 SNP rs200229088 = 0.37) has odds against causality > 10 million:1 versus signal 1 candidate rs10941679. Similarly, SNP rs7716600, which is an eQTL for MRPS30 expression^7^ (*r*^2^ with SNP rs10941679 = 0.77, with SNP rs6864776 = 0.05, and with SNP rs200229088 = 0.12) has odds against causality >160,000:1 versus signal 1 candidate rs10941679. These exclusions of former causal candidates highlight the need for fine-mapping studies before conducting functional analyses.

Haplotype analyses were conducted using the above three signal-representative variants, which generated eight haplotypes (Table 2). Haplotypes carrying the rare allele of signal 3 SNP rs200229088 conferred higher risks than corresponding haplotypes carrying the common allele, consistent with this allele having an independent effect. Haplotype G, carrying the minor alleles of both the signal 1 and 2 representative SNPs, is very rare and reveals that their risk alleles are negatively correlated, which is also consistent with our finding that signal 2 top SNP rs6864776 increases in significance after conditioning on signal 1 SNP rs10941679 (Table 1).

We examined the associations of these three SNPs in the Asian case-control studies within BCAC. SNP1 and SNP3 both replicated in the Asian studies and the relative risk estimates with overall breast cancer were consistent with those seen in the European population: per *g*-allele OR (rs10941679) = 1.09; 95% CI 1.04–1.15; p = 0.0009, conditional p = 0.0859 and per *t*-allele OR (rs200229088) = 1.09; 95% CI 1.02–1.15; p = 0.0065, conditional p = 0.9149 (Table 1). SNP2 was not replicated in Asians (per *a*-allele OR = 0.94; 95% CI 0.89–1.00; p = 0.034, conditional p = 0.8901) (Table 1).

We investigated the associations of these three signals with tumor subtypes based on ER status. SNP1 rs10941679 was largely associated with ER^+^ breast cancer (OR ER^+^ = 1.15; 95% CI 1.13–1.18; p = 8.35 × 10^−30^ versus OR ER^−^ disease = 1.04; 95% CI 1.00–1.08; p = 0.059; p heterogeneity = 1.5 × 10^−5^; Table 1) as was SNP3 rs200229088 (OR ER^+^ = 1.12; 95% CI 1.09–1.15; p = 7.51 × 10^−14^ versus OR ER^−^ = 1.03; 95% CI 0.99–1.09; p = 0.11, p heterogeneity = 0.02). By contrast, SNP2 rs6864776 was moderately associated with ER^−^ but not ER^+^ tumors (OR ER^−^ = 1.10; 95% CI 1.05–1.14; p = 2.55 × 10^−5^ versus OR ER^*+*^ = 1.02; 95% CI 0.99–1.05; p = 0.08; p heterogeneity = 0.01; Table 1).

**Figure 2:**
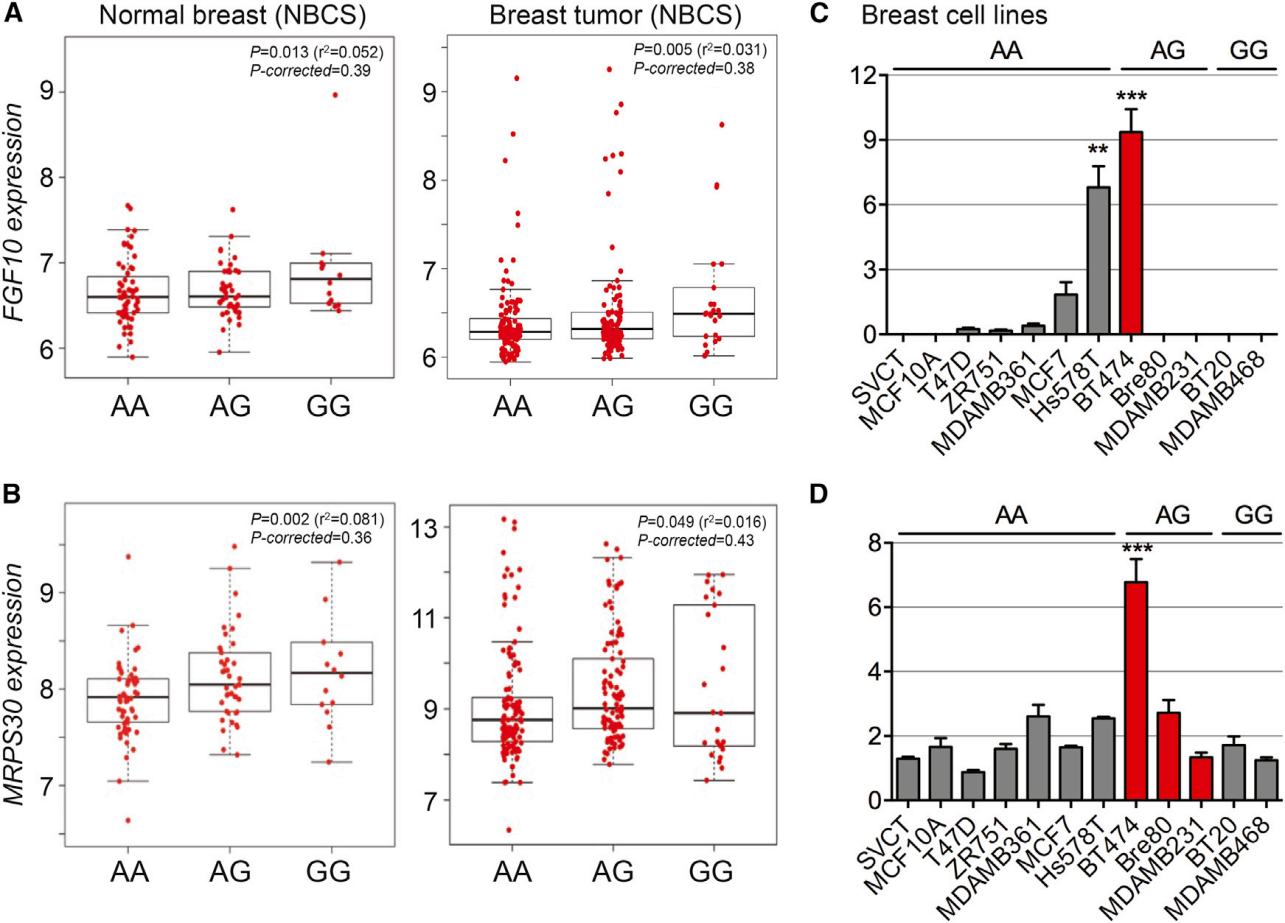
Association of rs10941679 with *FGF10* and *MRPS30* Expression in Normal Breast Tissues, Breast Tumors, and Breast Cancer Cell Lines (A and B) *FGF10* (A) or *MRPS30* (B) expression in normal breast (n = 116) or breast tumors from NBCS dataset (n = 241). SNP genotypes are shown on the x axis and log2-normalized gene expression values on the y axis. p values are presented before and after correction for multiple testing using FDR as implemented in p.adjust function in R. Each box plot shows the median rank normalized gene expression (horizontal line), the first through third quartiles (box), and 1.5× the interquartile range (whiskers). (C and D) Endogenous *FGF10* (Hs00610298_m1) (C) or *MRPS30* (Hs00169612_m1) (D) expression measured by qPCR in untreated breast cell lines and normalized to *GUSB* (4326320E). Error bars denote SEM (n = 3). p values were determined with a two-tailed t test. ^∗∗^p < 0.01, ^∗∗∗^p < 0.001.

Candidate SNPs 1–3 span a 1.7 Mb region on 5p12 that includes three annotated genes—*FGF10* (MIM: 602115), *MRPS30* (MIM: 611991), and *HCN1* (MIM: 602780)—and several putative long noncoding RNAs (lncRNAs; Figure 1). To identify potential target gene(s), we examined the associations of the three lead SNPs with expression levels of genes located within 1 Mb in three different studies: (1) 116 normal breast samples and 241 breast tumors from the Norwegian Breast Cancer Study (NBCS),^8^ (2) 93 normal and 765 breast cancer tissues from the TCGA study (germline genotype data from Affymetrix SNP 6 array were obtained from TCGA dbGAP data portal^9^), and (3) 183 normal breast samples from the Genotype-Tissue Expression (GTEx) project.^10^ The SNP1 rs10941679 risk-associated *g*-allele was moderately associated with increased *FGF10* mRNA expression in NBCS normal breast (p = 0.013, p corrected = 0.39) and breast tumors (p = 0.005, p corrected = 0.38) as well as in GTEx normal breast (p corrected = 0.02; Figures 2A and S1A). The effect in TCGA was in the same direction, though not significant (normal breast p = 0.353, p corrected = 0.95 and breast tumors p = 0.057, p corrected = 0.41; Figure S1B). The *g*-allele was also associated with increased expression of *MRPS30* in the NBCS normal (p = 0.002, p corrected = 0.36) and breast tumors (p = 0.049, p corrected = 0.43), in GTEx normal breast (p corrected = 0.002), and in TCGA (normal breast p = 6.86 × 10^−5^, p corrected = 5.31 × 10^−3^ and breast tumors p = 7.21 × 10^−6^, p corrected = 9.35 × 10^−4^; Figures 2B, S1A, and S1C). No associations were observed with SNP2 rs6864776 or SNP3 variant rs200229088. We also measured endogenous levels of *FGF10*, *MRPS30*, and nearby lncRNAs *FGF10-AS1*, *BRCAT54*, *RP11-503D12.1*, and *RP11-473L15.3* mRNA in breast cell lines homozygous (A/A or G/G) or heterozygous (A/G) for the common allele of SNP1 (Table S3, Figures 2C, 2D, S2, and S3). Total RNA from cell lines was extracted using Trizol and complementary DNA synthesized using random primers as per manufacturers’ instructions. Quantitative PCR (qPCR) were performed using TaqMan assays for *FGF10* and *MRPS30* normalized against beta-glucuronidase (*GUSB* [MIM: 611499]) or with SYTO9 for lncRNAs normalized against TATA box-binding protein (*TBP* [MIM: 600075]; primers are listed in Table S4). Although the number of ER^+^ breast cell lines carrying the risk allele was limited, *FGF10* and *MRPS30* mRNA levels were significantly higher in the BT474 heterozygous cell line (Figures 2C and 2D). *BRCAT54* was detected in the majority of cell lines but its expression appears to be genotype independent (Figure S3A). *FGF10-AS1*, *RP11-503D12.1*, and *RP11-473L15.3* transcripts were either expressed at very low levels or not detected in the cell lines analyzed (Figures S3B–S3D). Therefore, although we cannot rule out the possibility that the risk SNPs may influence local lncRNA expression, the low or absent transcript levels precluded any further evaluation.

Candidate causal SNPs were then explored using publicly available datasets from ENCODE,^11^ which includes information such as the location of promoter and enhancer histone marks, open chromatin, bound proteins, and altered motifs for the MCF7 breast cancer cell line, and from Hnisz et al.^12^ and Corradin et al.^13^ to identify the location of likely enhancers and their gene targets in a cell-specific context. Analysis of *cis* enhancer-gene interactions via PreSTIGE^13^ showed evidence of putative regulatory elements (PREs) surrounding the top risk-associated SNPs in MCF7 breast cancer cells, but no histone-marked elements harboring a risk SNP in this cell line or in a range of cell lines and tissues analyzed in Roadmap (Figures 1 and S4). However, it is possible that certain epigenetic marks may be detected only in a specific cell subtype such as breast stem cells or in response to an external stimulus.

**Figure 3:**
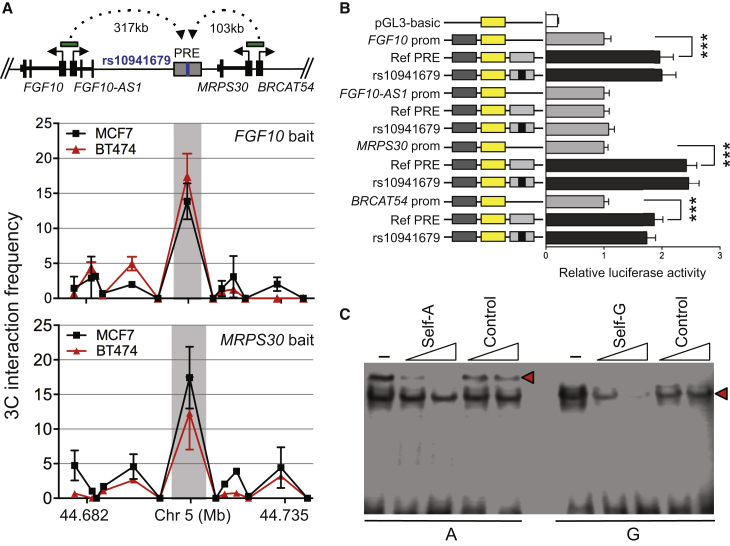
Distal Regulation of *FGF10* and *MRPS30* at the 5p12 Risk Region (A) 3C interaction profiles between the *FGF10/FGF10AS-1* or *MRPS30/BRCAT54* bidirectional promoters and the putative regulatory element (PRE; gray bar) containing SNP rs10941679. Anchor points are set at the promoters. Graphs represent one of three independent experiments (see Figure S5B). Error bars denote SD. (B) Luciferase reporter assays after transient transfection of ER^+^ BT474 breast cancer cell lines. The PRE containing the major SNP allele was cloned downstream of target gene promoter-driven luciferase constructs (Ref PRE). The risk *g*-allele was engineered into the constructs and designated by the rs ID. Primers are listed in Table S7. Error bars denote 95% confidence intervals from three independent experiments. p values were determined by 2-way ANOVA followed by Dunnett’s multiple comparisons test (^∗∗∗^p < 0.001). (C) EMSA for oligonucleotides containing SNP rs1094617 with the A = common allele and G = minor allele as indicated below the panel, assayed using BT474 nuclear extracts. Primers are listed in Table S8. Labels above each lane indicate inclusion of competitor oligonucleotides at 30- and 100-fold molar excess, respectively: (-) no competitor and control denotes a non-specific competitor. A red arrowhead shows a band of different mobility detected between the common and minor alleles.

To identify target gene(s), we performed chromatin conformation capture (3C) assays in ER^+^ MCF7, BT474, and MDA-MB-361 and ER^−^ MDA-MB-231 breast cancer cell lines and Bre80 normal breast cells (Table S5).^8^ 3C libraries were created by cross-linking the chromatin from cell lines; DNA was then digested with EcoRI, which flanks 12 contiguous fragments that cover the PRE, and the *FGF10*, *MRPS30*, and *HCN1* promoters (Table S6); DNA was religated and decrosslinked; and qPCR with primers for the bait (gene promoters) and interactors (12 PRE fragments) was performed to detect the presence of ligation products, representing gene loops. BAC clones covering the regions of interest were used to normalize for PCR efficiency. These assays showed that the PRE containing SNP1 frequently interacted with the *FGF10* and *MRPS30* promoter regions in MCF7 and BT474 breast cancer cell lines, but only with *MRPS30* in the MDA-MB-361, MDA-MB-231, and Bre80 cell lines. This latter result was expected because *FGF10* is not expressed or expressed at very low levels in these cell lines (Figures 2C, 3A, S5, and S6). Notably, both genes share a bidirectional promoter with the lncRNAs *FGF10-AS1* and *BRCAT54*, raising the possibility that these transcripts are also targets of the PRE (Figure 3A). No additional interactions were detected between the PRE and other annotated genes within 1 Mb of the PRE, including *HCN1* (Figure S5). To assess the potential impact of SNP1 on the identified chromatin interactions, allele-specific 3C was performed in heterozygous BT474 cell lines.^8^ However, the sequence profiles revealed that SNP1 had no significant effect on chromatin looping (Figure S7).

The regulatory capability of the PRE, combined with the effect of SNP1, was further examined in reporter assays. Promoter-driven luciferase reporter constructs were generated by the insertion of PCR-amplified fragments containing *FGF10*, *FGF10-AS1*, *MRPS30*, or *BRCAT54* promoters into pGL3-Basic.^14^ A 1,736-bp PRE fragment (containing either the common or minor allele of rs10941679) was then generated by PCR and cloned downstream of the modified pGL3-promoter constructs (Table S7). MCF7 and BT474 breast cancer cell lines plus Bre80 normal breast cells were transfected with the reporter plasmids and luciferase activity was measured 24 hr after transfection. To correct for any differences in transfection efficiency or cell lysate preparation, *Firefly* luciferase activity was normalized to *Renilla*. Notably, the “Ref PRE” acted as a transcriptional enhancer, leading to a 2- to 3-fold increase in *FGF10*, *MRPS30*, and *BRCAT54* promoter activity, but had no effect on the *FGF10-AS1* promoter in MCF7 and BT474 cells (Figures 3B and S8). The enhancer activity was also observed for the *MRPS30* and *BRCAT54* promoters in Bre80 cells (Figure S8). In all cell lines, inclusion of the SNP1 risk (*g*) allele had no significant effect on the PRE enhancer activity. Although this appears to rule out an effect of this SNP on transactivation, it is possible that SNP1 affects the recruitment of key proteins required for the epigenetic modification of the enhancer, which would not be observed in a reporter assay. Another possibility is that the SNP effect may be observed only under certain biological conditions such as growth factor stimulation.

To seek further evidence that SNP1 lies within an enhancer element, we performed electrophoretic mobility shift assays (EMSAs) for both the protective (*a*) and risk (*g*) alleles.^15^ Nuclear lysates were prepared from ER^+^ BT474, MCF7, and MDA-MB-361 or ER^−^ MDA-MB-231 and Hs578T cells using the NE-PER nuclear and cytoplasmic extraction reagents. Biotinylated oligonucleotide duplexes were prepared by combining sense and antisense oligonucleotides, heat annealing, and slow cooling. Duplex-bound complexes were transferred onto Zeta-Probe positively charged nylon membranes by semi-dry transfer then cross-linked onto the membranes. Membranes were processed with the LightShift Chemiluminescent EMSA kit as per the manufacturer’s instructions, and signals were visualized with the C-DiGit blot scanner. For SNP1, we observed allele-specific binding by nuclear proteins only in the ER^+^ BT474, MCF7, and MDA-MB-361 extracts (Figures 3C and S9). The protein-DNA complexes were shown to be specific, as demonstrated by increasing amounts of cold self-competitor (Figures 3C and S9 and Table S8).

Further EMSAs using competitor DNA or antibody supershifts against predicted transcription factors (TFs) suggested four proteins bound to the SNP site including FOXA1, FOXA2, CEBPB, and OCT1 (Figure S10 and Table S9). To confirm TF binding in vivo, we performed chromatin immunoprecipitation (ChIP) in heterozygous BT474 cells as previously described (Table S10).^15^ When compared to an IgG control antibody, we observed a moderate enrichment in FOXA1 and OCT1 binding to DNA overlapping SNP rs10941679, but no difference between alleles in this cell line (Figure S11). In addition, western blot analysis indicated that FOXA1 protein expression was restricted to the ER^+^ breast cancer cell lines analyzed, whereas OCT1 was more widely expressed (Figure S12). FOXA1 is a pioneer factor and master regulator of ER activity due to its ability to open local chromatin and recruit ER to target gene promoters.^16^ Notably, breast cancer-associated SNPs are enriched for FOXA1 binding^17^ and several studies have linked cooperative binding of FOXA1, ER, and OCT1 to increased gene transcription.^18^, ^19^ Consistent with our eQTL data, it is tempting to speculate that in specific ER^+^ cell subtypes and/or conditions, rs10941679 alters FOXA1 affinity and OCT1 recruitment leading to target gene activation.

In conclusion, we have provided evidence for at least three independent causal SNPs with effects on the risk of breast cancer at this locus. The minor *g*-allele of signal 1 SNP rs10941679 conferred a 15% increased risk of ER^+^ breast cancer and higher expression levels of the *MRPS30* and *FGF10* genes and was the most strongly associated SNP with *MRPS30* expression in this 1 Mb region. MRPS30—also called PDCD9 (Programmed Cell Death protein 9)—encodes a mitochondrial ribosomal protein involved in apoptosis.^20^ Although the role of mitochondria in apoptosis remains unclear, it is well established that cytochrome *c* and other pro-apoptotic proteins are released during cell death initiation.^20^ Clearly, further investigation of the function of this protein is now merited. By contrast, *FGF10* is an extensively studied gene with compelling data suggesting its involvement in breast tumorigenesis. *FGF10* is a member of the fibroblast growth factor (FGF) family and encodes a glycoprotein that specifically binds to FGFR2 (splice FGFR2IIIb) to control signaling pathways including cell differentiation, proliferation, and apoptosis.^21^ Variants regulating *FGFR2* (MIM: 176943) have the strongest association with ER^+^ breast cancer susceptibility identified to date.^22^ FGF10 is overexpressed in ∼10% of human breast cancers^23^ and increased levels of *FGF10* are highly correlated with proliferation rate of breast cancer cell lines and cancer cell invasion.^24^, ^25^ It signals through multiple downstream pathways including MAPK and WNT and genes such as *FGFR2*, *CCND1* (MIM: 168461), and *TGFB1* (MIM: 190180),^21^, ^24^ all known to play key roles in breast cancer. Therapeutic targeting of FGFs and their receptors (FGFRs) is currently a major area of drug development research, and the identification of a subgroup of individuals diagnosed with breast cancer with alterations in these pathways may open new avenues for personalized medicine and pathway-targeted treatments.

## Figures and Tables

**Table 1:** Associations of the Top SNPs from Each Signal with Overall Breast Cancer Risk and Breast Cancer Stratified by ER Status

**Sig**	**SNP**	**Com**	**Min**	**MAF^∗^**	**OR Overall 95% CI**	**p Overall**	**Conditional p Value**	**OR ER**^**−**^	**p ER**^**−**^	**OR ER**^**+**^	**p ER**^**+**^
**Europeans**											

1	rs10941679	A	G	0.27	1.12 (1.10–1.14)	2.55 × 10^−26^	6.55 × 10^−24^	1.04 (1–1.08)	0.059	1.15 (1.13–1.18)	8.35 × 10^−30^
2	rs6864776	G	A	0.23	1.04 (1.02–1.06)	7.84 × 10^−4^	1.44 × 10^−12^	1.10 (1.05–1.14)	2.5 × 10^−5^	1.02 (0.99–1.05)	0.08
3	rs200229088	TTG	T	0.31	1.09 (1.07–1.12)	2.28 × 10^−12^	1.12 × 10^−5^	1.03 (0.99–1.09)	0.11	1.12 (1.09–1.15)	7.51 × 10^−14^

**Asians**											

1	rs10941679	A	G	0.50	1.09 (1.04–1.15)	9.12 × 10^−4^	0.0859	1.03 (0.95–1.11)	0.53	1.11 (1.04–1.18)	1.32 × 10^−3^
2	rs6864776	G	A	0.32	0.94 (0.89–1.00)	3.47 × 10^−2^	0.8901	0.95 (0.87–1.04)	0.28	0.94 (0.89–1.00)	6.24 × 10^−2^
3	rs200229088	TTG	T	0.37	1.09 (1.02–1.15)	6.52 × 10^−3^	0.9149	1.04 (0.95–1.14)	0.43	1.08 (1.00–1.16)	3.65 × 10^−2^

Abbreviations are as follows: Com, common alleles; Min, minor alleles; MAF, minor allele frequency; OR, per-allele odds ratios (OR); 95% CI, 95% confidence intervals and 1 degree of freedom; p, significance levels for overall breast cancer are indicated in European and Asian case-control studies, and separately for ER^+^ and ER^−^ disease.

**Table 2:** Haplotype Analysis across the BCAC Studies

**Haplotypes**	**rs10941679 Signal 1**	**rs6864776 Signal 2**	**rs200229088 Signal 3**	**Haplotype Frequency**	**OR**	**p Value**
A	1	1	1	0.395440	–	–
B	1	1	2	0.120099	1.06 (1.02–1.10)	1.49 × 10^−3^
C	1	2	1	0.199599	1.10 (1.06–1.13)	7.76 × 10^−11^
D	1	2	2	0.018665	1.15 (1.04–1.27)	5.03 × 10^−3^
E	2	1	1	0.098169	1.14 (1.09–1.19)	1.45 × 10^−11^
F	2	1	2	0.154525	1.20 (1.16–1.24)	2.72 × 10^−30^
G	2	2	1	0.004248	0.91 (0.72–1.15)	4.15 × 10^−1^
H	2	2	2	0.009253	1.28 (1.10–1.48)	1.14 × 10^−3^

Each haplotype was compared to the ancestral haplotype carrying the common alleles of signal 1 SNP rs10941679, signal 2 SNP rs6864776, and signal 3 SNP rs200229088 (haplotype A).

## Web Resources

1000 Genomes, http://www.1000genomes.org
Cancer Cell Line Encyclopedia (CCLE), https://portals.broadinstitute.org/ccle/home
ENCODE, https://www.encodeproject.org/
GEO, http://www.ncbi.nlm.nih.gov/geo/
GTEx Portal, http://www.gtexportal.org/home/
OMIM, http://www.omim.org/
PreSTIGE, http://genetics.case.edu/prestige/
The Cancer Genome Atlas, http://cancergenome.nih.gov/

## Supplemental Data

Document S1. Supplemental Acknowledgments, Figures S1–S12, Tables S1–S10, and Consortia Information Document S1. Supplemental Acknowledgments, Figures S1–S12, Tables S1–S10, and Consortia Information

Document S2. Article plus Supplemental Data Document S2. Article plus Supplemental Data
