# Supplementary material for: Evidence that the 5p12 Variant rs10941679 Confers Susceptibility to Estrogen-Receptor-Positive Breast Cancer through FGF10 and MRPS30 Regulation
Source: Am J Hum Genet. 2016 Sep 15;99(4):903–11. doi: 10.1016/j.ajhg.2016.07.017 (PMC5065698; doi:10.1016/j.ajhg.2016.07.017)
Supplement: Document S1. Supplemental Acknowledgments, Figures S1–S12, Tables S1–S10, and Consortia Information [file mmc1.pdf]

The American Journal of Human Genetics, Volume *99*

Supplemental Data

Evidence that the 5p12 Variant rs10941679 Confers Susceptibility to Estrogen-Receptor-Positive Breast Cancer through *FGF10* and *MRPS30* Regulation

Maya Ghoussaini, Juliet D. French, Kyriaki Michailidou, Silje Nord, Jonathan Beesley, Sander Canisus, Kristine M. Hillman, Susanne Kaufmann, Haran Sivakumaran, Mahdi Moradi Marjaneh, Jason S. Lee, Joe Dennis, Manjeet K. Bolla, Qin Wang, Ed Dicks, Roger L. Milne, John L. Hopper, Melissa C. Southey, Marjanka K. Schmidt, Annegien Broeks, Kenneth Muir, Artitaya Lophatananon, Peter A. Fasching, Matthias W. Beckmann, Olivia Fletcher, Nichola Johnson, Elinor J. Sawyer, Ian Tomlinson, Barbara Burwinkel, Frederik Marme, Pascal Guénel, Thérèse Truong, Stig E. Bojesen, Henrik Flyger, Javier Benitez, Anna González-Neira, M. Rosario Alonso, Guillermo Pita, Susan L. Neuhausen, Hoda Anton-Culver, Hermann Brenner, Volker Arndt, Alfons Meindl, Rita K. Schmutzler, Hiltrud Brauch, Ute Hamann, Daniel C. Tessier, Daniel Vincent, Heli Nevanlinna, Sofia Khan, Keitaro Matsuo, Hidemi Ito, Thilo Dörk, Natalia V. Bogdanova, Annika Lindblom, Sara Margolin, Arto Mannermaa, Veli-Matti Kosma, kConFab/AOCS Investigators, Anna H. Wu, David Van Den Berg, Diether Lambrechts, Giuseppe Floris, Jenny Chang-Claude, Anja Rudolph, Paolo Radice, Monica Barile, Fergus J. Couch, Emily Hallberg, Graham G. Giles, Christopher A. Haiman, Loic Le Marchand, Mark S. Goldberg, Soo H. Teo, Cheng Har Yip, Anne-Lise Borresen-Dale, NBCS Collaborators, Wei Zheng, Qiuyin Cai, Robert Winqvist, Katri Pylkäs, Irene L. Andrulis, Peter Devilee, Rob A.E.M. Tollenaar, Montserrat García-Closas, Jonine Figueroa, Per Hall, Kamila Czene, Judith S. Brand, Hatef Darabi, Mikael Eriksson, Maartje J. Hooning, Linetta B. Koppert, Jingmei Li, Xiao-Ou Shu, Ying Zheng, Angela Cox, Simon S. Cross, Mitul Shah, Valerie Rhenius, Ji-Yeob Choi, Daehee Kang, Mikael Hartman, Kee Seng Chia, Maria Kabisch, Diana Torres, Craig Luccarini, Don M. Conroy, Anna Jakubowska, Jan Lubinski, Suleeporn Sangrajrang, Paul Brennan, Curtis Olswold, Susan Slager, Chen-Yang Shen, Ming-Feng Hou, Anthony Swerdlow, Minouk J. Schoemaker, Jacques Simard, Paul D.P. Pharoah, Vessela Kristensen, Georgia Chenevix-Trench, Douglas F. Easton, Alison M. Dunning, and Stacey L. Edwards

SUPPLEMENTAL ACKNOWLEDGMENTS

We thank all the individuals who took part in these studies and all the researchers, clinicians, technicians and administrative staff who have enabled this work to be carried out. The procedures followed were in accordance with the ethical standards of the responsible committee on human experimentation (institutional and national) and that proper informed consent was obtained. This study would not have been possible without the contributions of the following: Andrew Berchuck (OCAC), Rosalind A. Eeles, Ali Amin Al Olama, Zsofia Kote-Jarai, Sara Benlloch (PRACTICAL), Craig Luccarini and the staff of the Centre for Genetic Epidemiology Laboratory, the staff of the CNIO genotyping unit, Daniel C. Tessier, Francois Bacot, Daniel Vincent, Sylvie LaBoissière and Frederic Robidoux and the staff of the McGill University and Génome Québec Innovation Centre, Sune F. Nielsen, Borge G. Nordestgaard, and the staff of the Copenhagen DNA laboratory, and Julie M. Cunningham, Sharon A. Windebank, Christopher A. Hilker, Jeffrey Meyer and the staff of Mayo Clinic Genotyping Core Facility. Normal human tissues from the Susan G. Komen for the Cure® Tissue Bank at the IU Simon Cancer Center, Indianapolis were used in this study. We thank contributors, including Indiana University who collected samples used in this study, as well as donors and their families, whose help and participation made this work possible. Also NIHR Support to the Royal Marsden Biomedical Research Centre. Funding for the iCOGS infrastructure came from: the European Community's Seventh Framework Programme under grant agreement n° 223175 (HEALTH-F2-2009-223175) (COGS), Cancer Research UK (C1287/A10118, C1287/A10710, C12292/A11174, C1281/A12014, C5047/A8384, C5047/A15007, C5047/A10692, C8197/A16565), the National Institutes of Health (CA128978) and Post-Cancer GWAS initiative (1U19 CA148537, 1U19 CA148065 and 1U19 CA148112 - the GAME-ON initiative), the Department of Defence (W81XWH-10-1-0341), the Canadian Institutes of Health Research (CIHR) for the CIHR Team in Familial Risks of Breast Cancer, Komen Foundation for the Cure, the Breast Cancer Research Foundation, and the Ovarian Cancer Research Fund. The QIMR Berghofer group was supported by a National Health and Medical Research Council of Australia project grant (1058415). GCT is an NHMRC Senior Principal Research Fellow. SLE and JDF are supported by Fellowships from the National Breast Cancer Foundation (NBCF) Australia. SN is supported by a carrier grant from the Norwegian Regional Health authorities (Grant number 2014061). The funders have no role in study design, data collection and analysis, decision to publish, or preparation of the manuscript. The authors declare no competing financial interests.

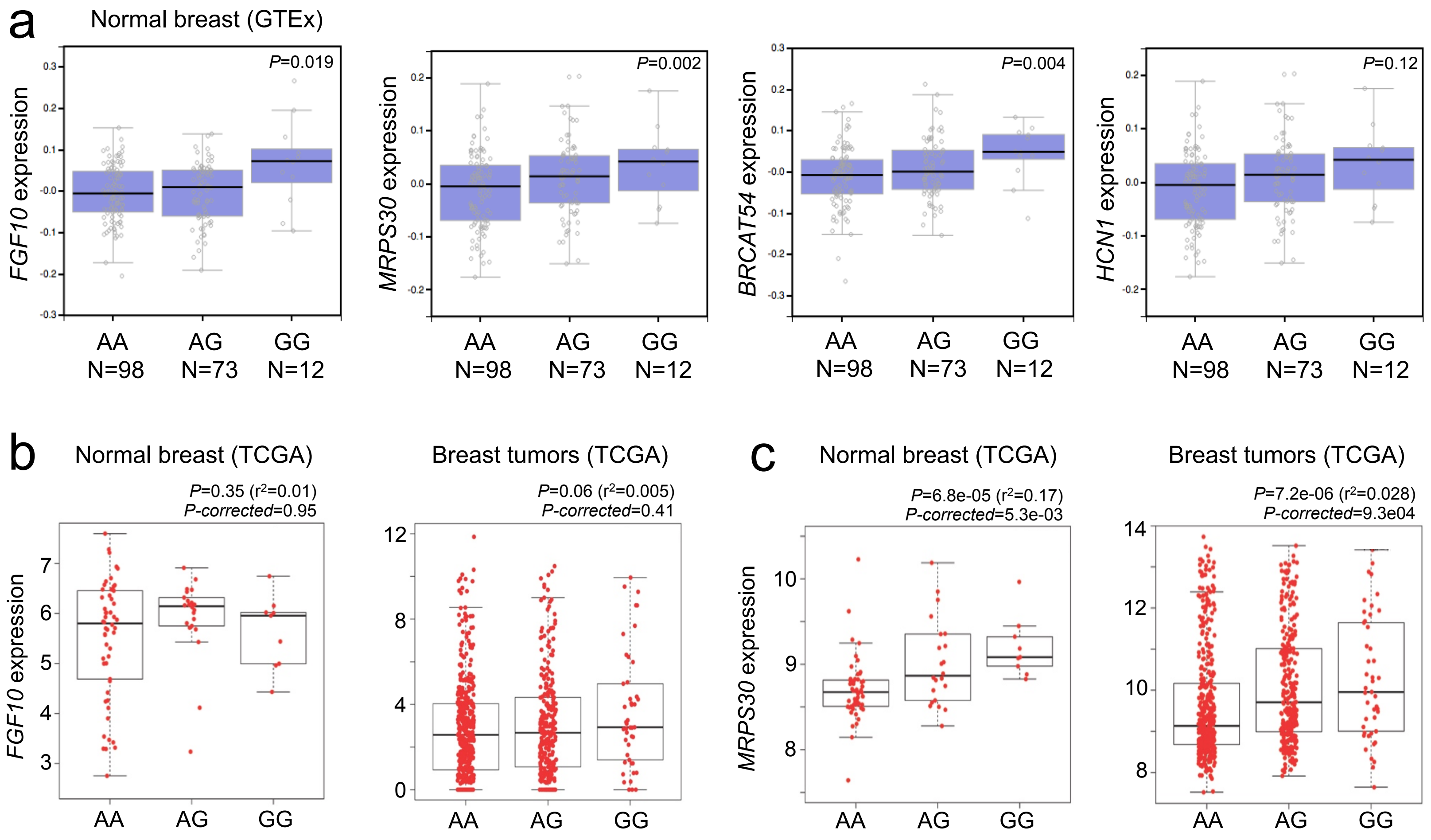

Supplementary Figure 1. Associations of SNP rs10941679 with expression of candidate target genes. (a) 183 normal breast samples from the GTEx database, **(b)** *FGF10* or **(c)** *MRPS30* in normal breast TCGA (n=93) and breast tumours TCGA (n=765). The *x*-axis of each plot corresponds to the three observed SNP genotypes and the *y*-axis represents log2-normalized gene expression values. *P*-values in the GTEx datasets are pre-corrected for multiple testing. *P*-values in the TCGA datasets are presented before and after correction for multiple testing using FDR as implemented in p.adjust function in R. Each box plot shows the median rank normalized gene expression (horizontal line), the first through third quartiles (box) and 1.5× the interquartile range (whiskers).

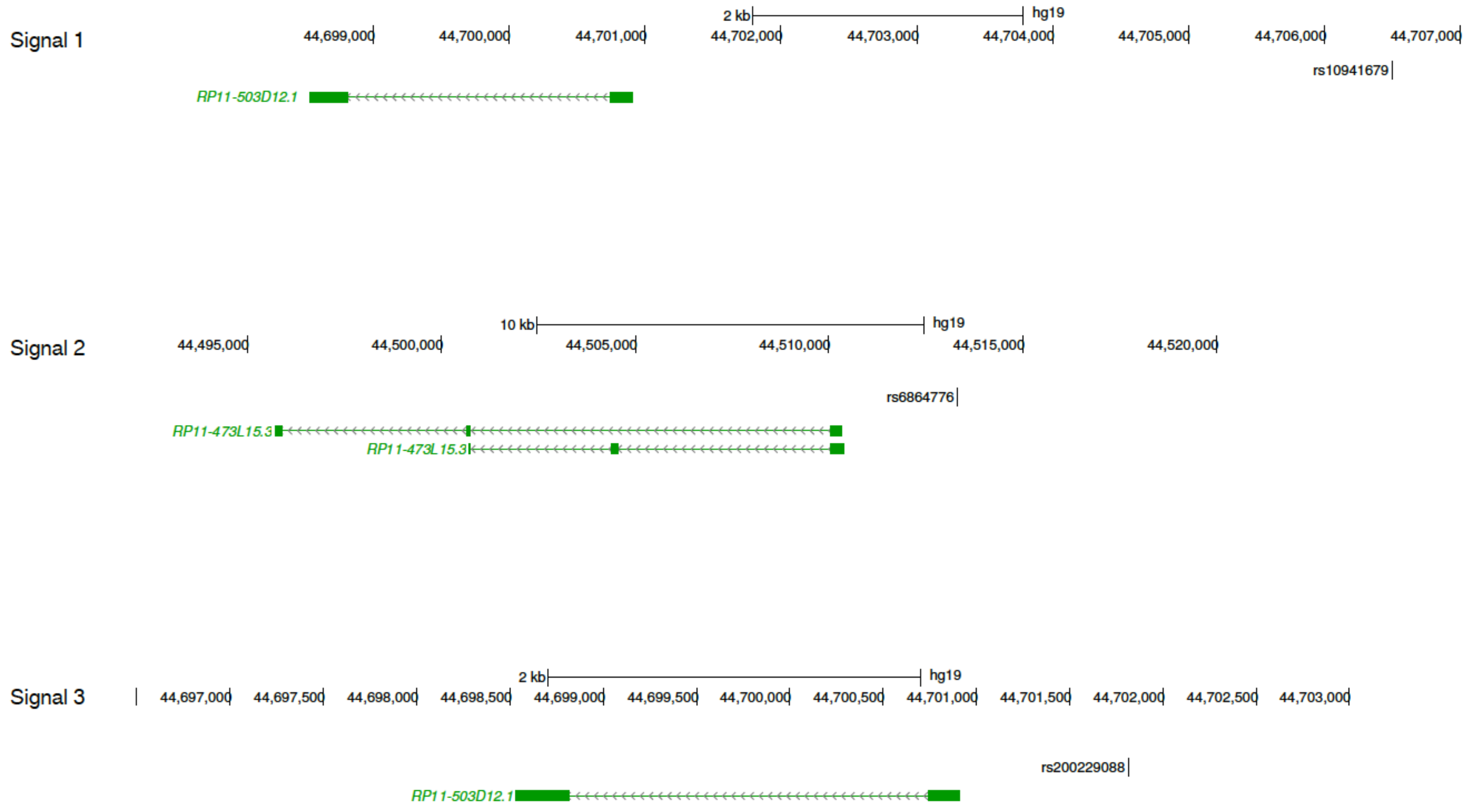

Supplementary Figure 2. Location of putative noncoding RNAs close to the top risk SNPs. The top risk SNPs from each signal are shown as small horizontal lines and labelled. Green boxes represent putative noncoding RNAs.

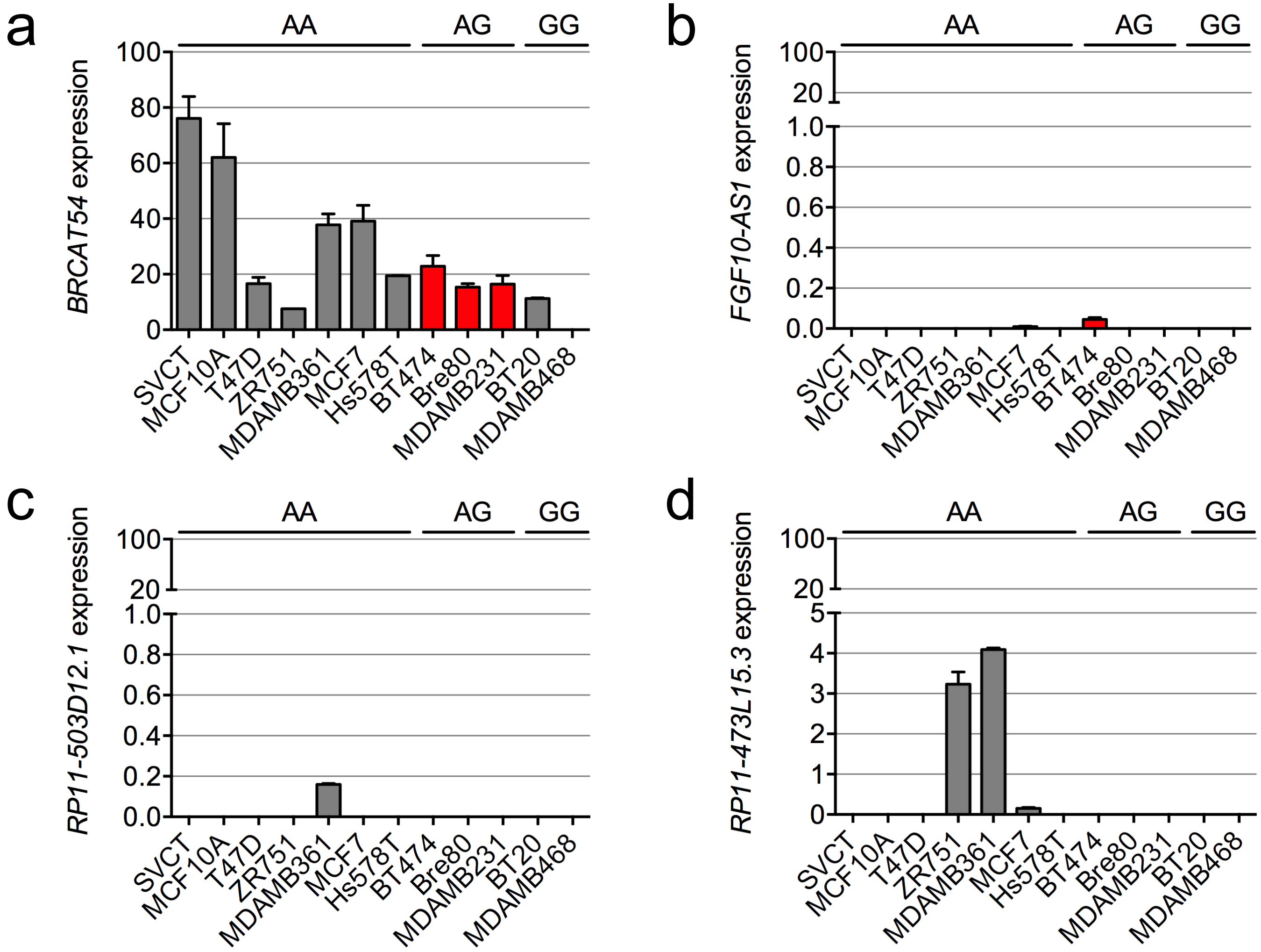

Supplementary Figure 3. Putative noncoding RNA expression measured by qPCR in untreated breast cell lines and normalised to *TBP*. Error bars denote SEM (n=3).

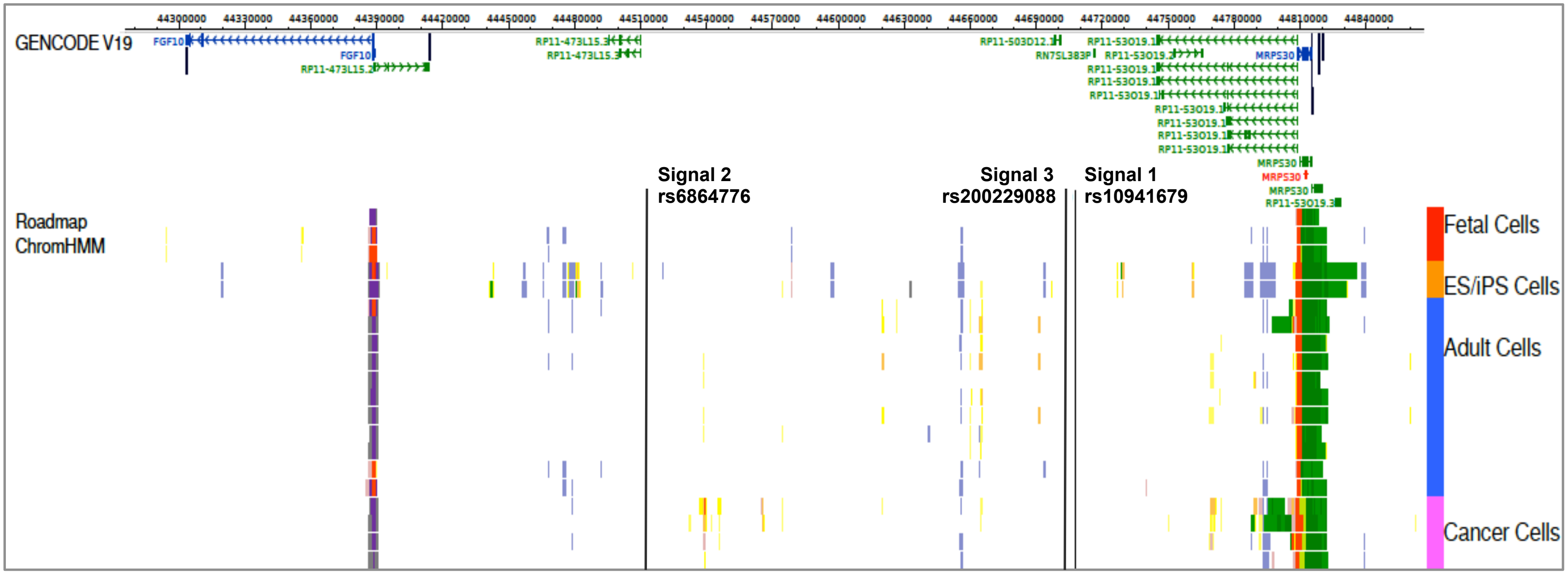

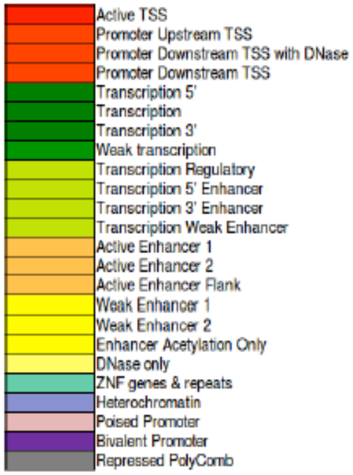

Supplementary Figure 4. Genomic region (chr5:44276112-44866764) showing chromatin state annotations called by Roadmap Epigenomics Project Chromatin Hidden Markov Modelling (Imputed ChromHMM) relative to GENCODE V19 gene models. The 25 states of chromatin segmentation depicted as colors along the genomic axis are described in the key. The position of top risk SNPs for signals 1-3 are shown by the vertical black lines.

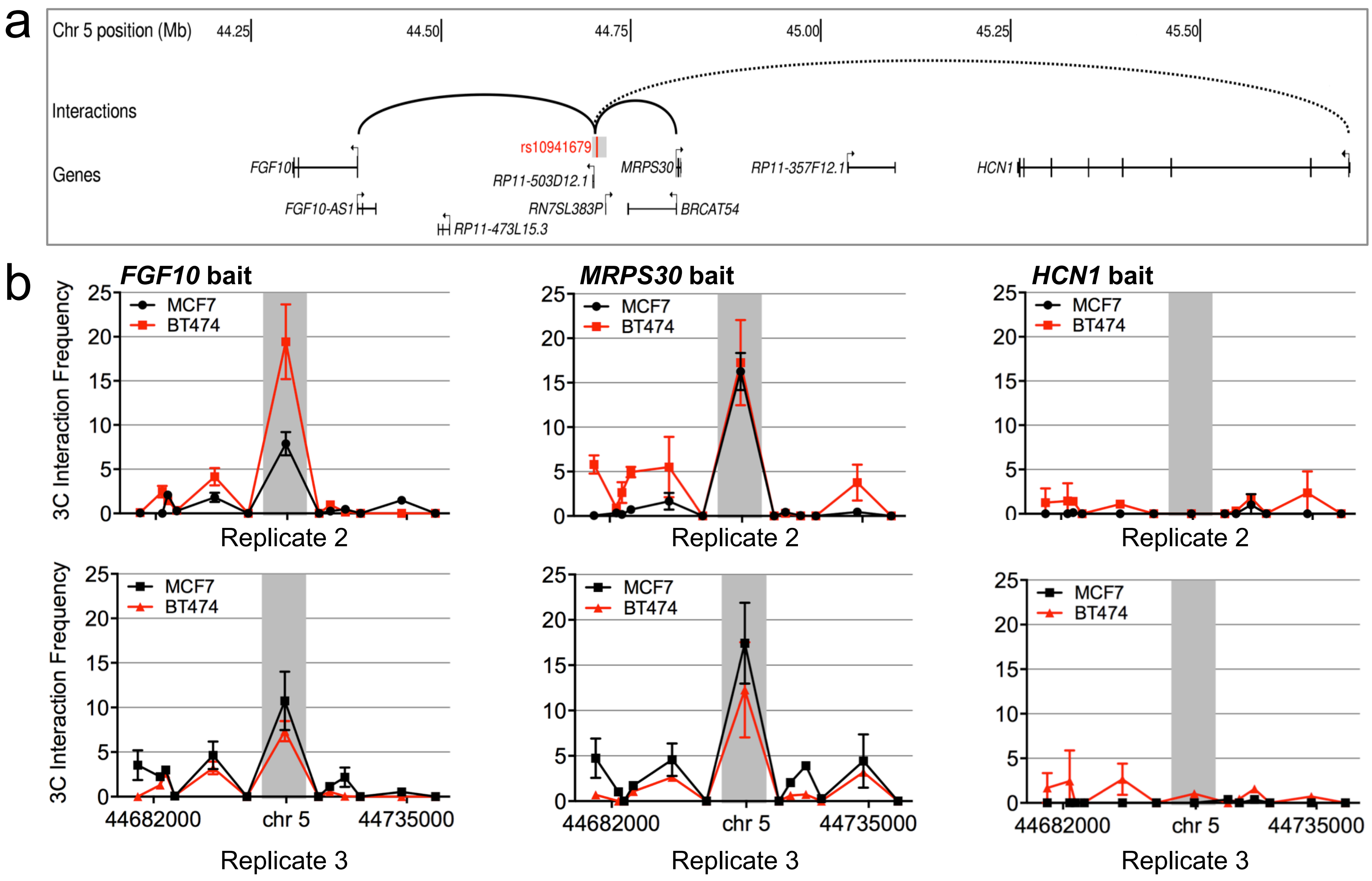
a
Chr 5 position (Mb)
44.25
44.50
44.75
45.00
45.25
45.50
Interactions
Genes
rs10941679
FGF10
MRPS30
RP11-357F12.1
HCN1
RP11-503D12.1
FGF10-AS1
RN7SL383P
BRCAT54
RP11-473L15.3
b
FGF10 bait
MRPS30 bait
HCN1 bait
3C Interaction Frequency
MCF7
BT474
Replicate 2
Replicate 3
44682000
chr 5
44735000

C

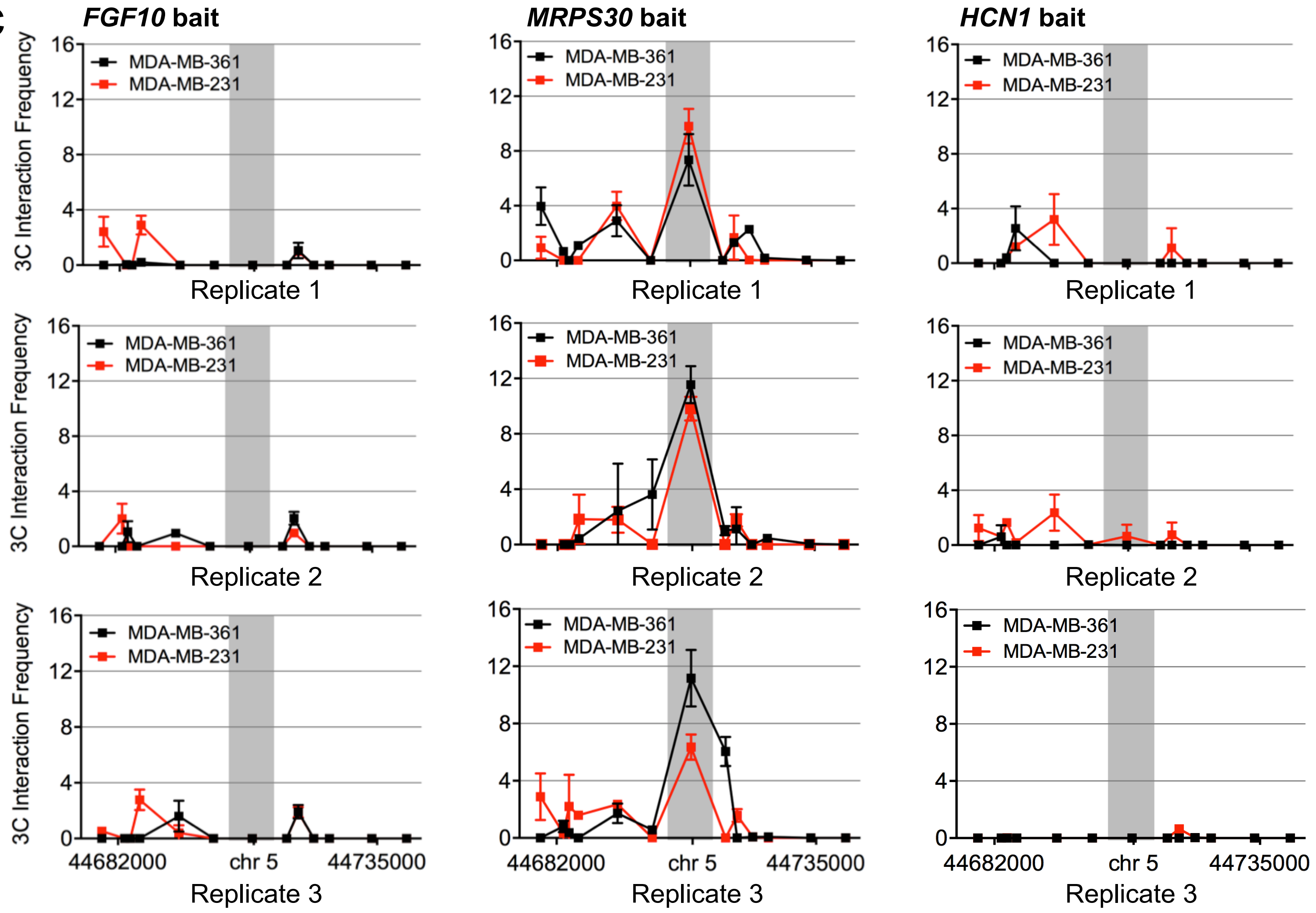
FGF10 bait
MRPS30 bait
HCN1 bait
3C Interaction Frequency
MDA-MB-361
MDA-MB-231
Replicate 1
Replicate 2
Replicate 3
44682000
chr 5
44735000
0
4
8
12
16

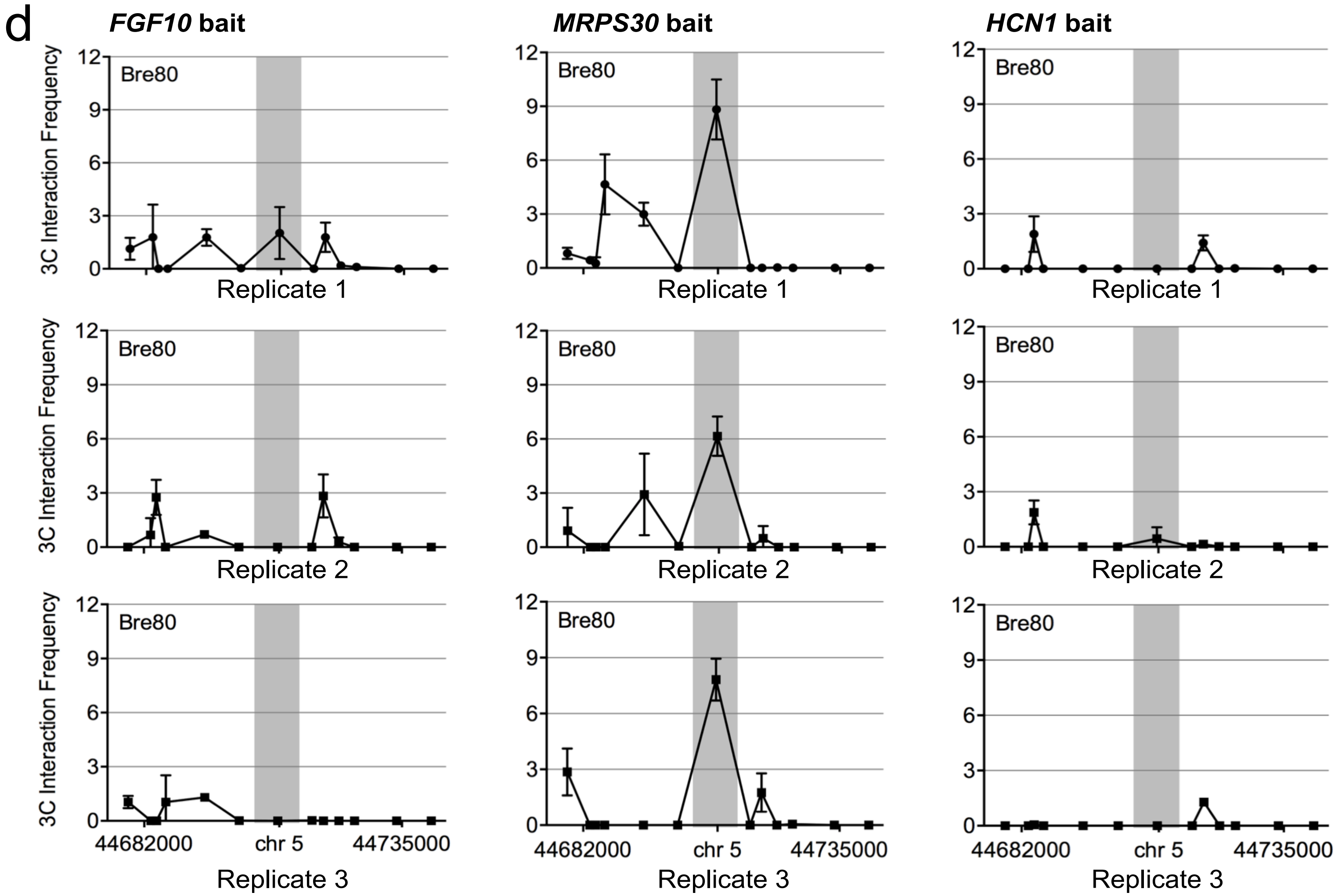

Supplementary Figure 5. **Chromatin interactions at 5p12 in breast cancer cell lines**. **(a)** Physical map of the region interrogated by 3C. 3C interaction profiles between the *FGF10/FGF10AS-1*, *MRPS30/BRCAT54* or *HCN1* promoters and the putative regulatory element (PRE, grey bars) containing SNP rs10941679 in **(b)** ER^+/PR^+ MCF7 and BT474, **(c)** ER^+/PR^- MDA-MB-361 or ER^-/PR^- MDA-MB-231 breast cancer cell lines and **(d)** ER^- Bre80 normal breast cells. 3C libraries were generated with *Eco*RI*,* with the anchor points set at the promoters. Error bars denote SD.

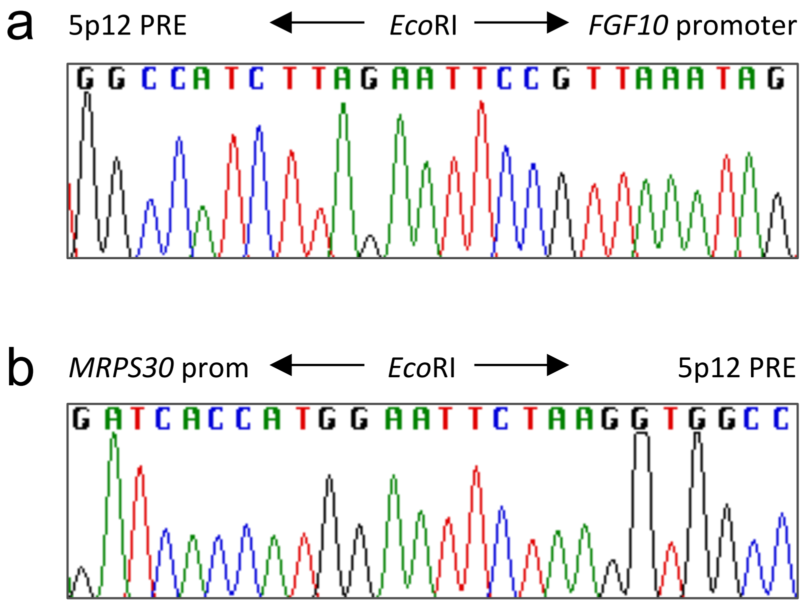

Supplementary Figure 6. DNA sequencing to confirm chromatin interactions. Chromatogram of 3C products amplified between the **(a)** *FGF10* or **(b)** *MRPS30* promoters and the 5p12 putative regulatory element (PRE) containing SNP rs10941679.

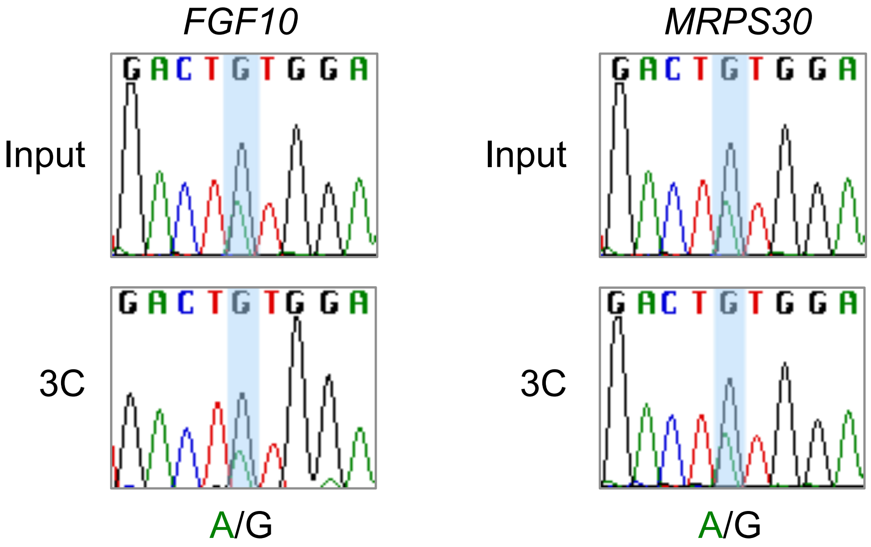

Supplementary Figure 7. SNP rs10941679 does not influence chromatin looping between the PRE and *FGF10 or MRPS30* promoters. 3C followed by sequencing for the rs10941679-containing region in heterozygous BT474 ER+ breast cancer cells.

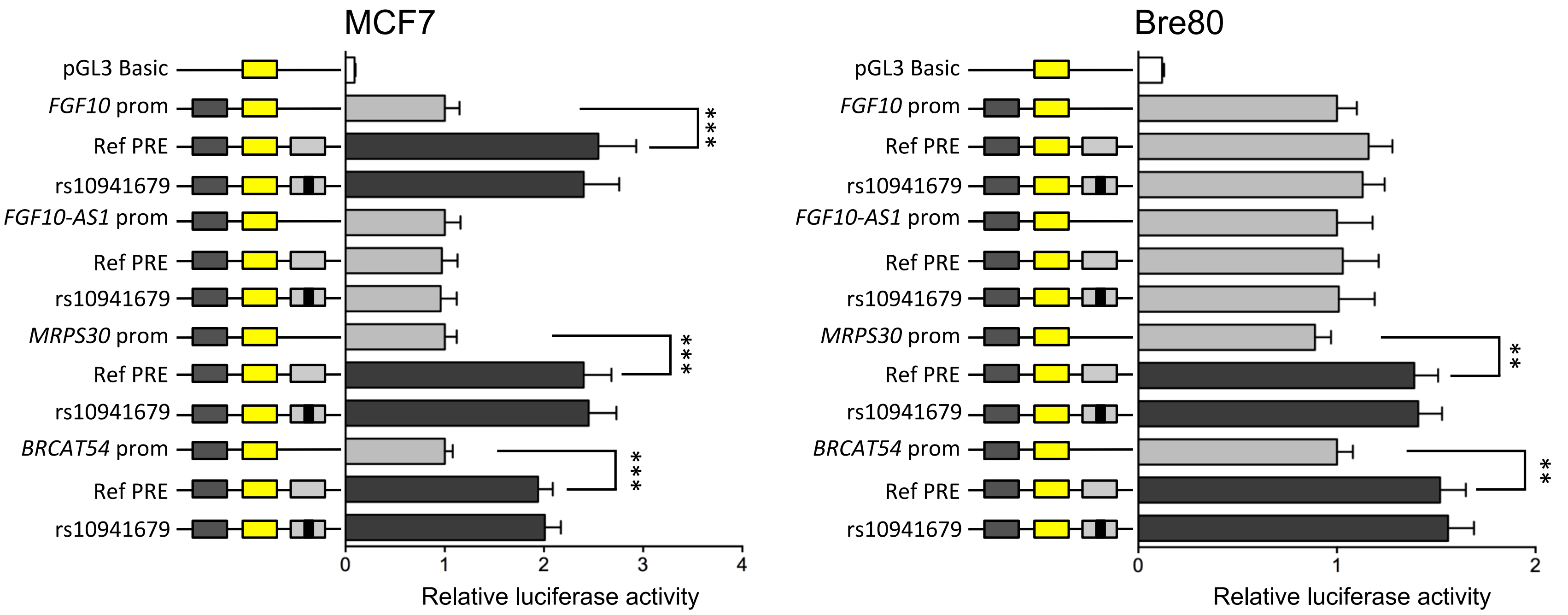

Supplementary Figure 8. Luciferase reporter assays in ER+ MCF7 and ER- Bre80 breast cell lines. The putative regulatory element containing the major SNP allele was cloned downstream of target gene promoter-driven luciferase constructs (Ref PRE). The minor SNP allele was engineered into the constructs and is designated by the rs ID. Error bars denote 95% confidence intervals (n=3). *P*-values were determined by 2-way ANOVA followed by Dunnett's multiple comparisons test. $^{**}P<0.01$, $^{***}P<0.001$.

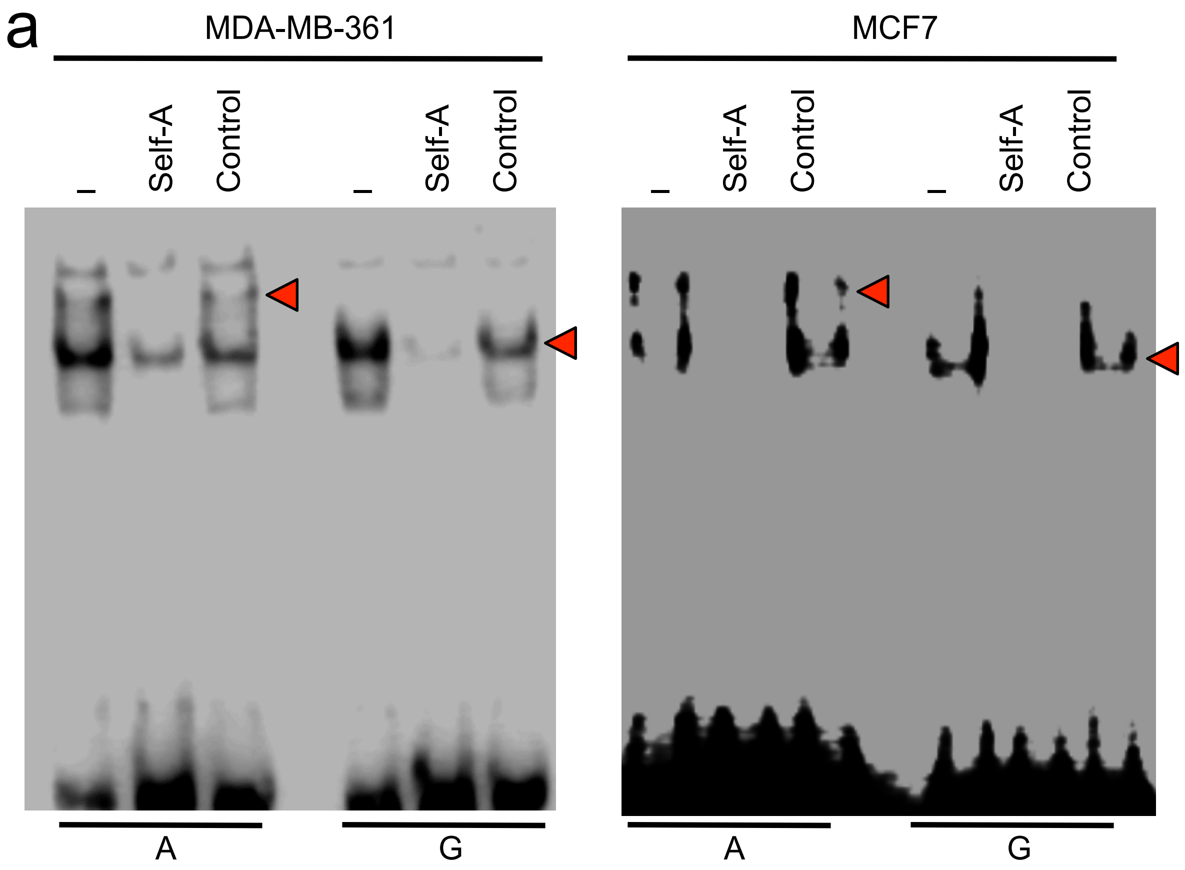
a
MDA-MB-361
MCF7
–
Self-A
Control
–
Self-A
Control
–
Self-A
Control
–
Self-A
Control
A
G
A
G

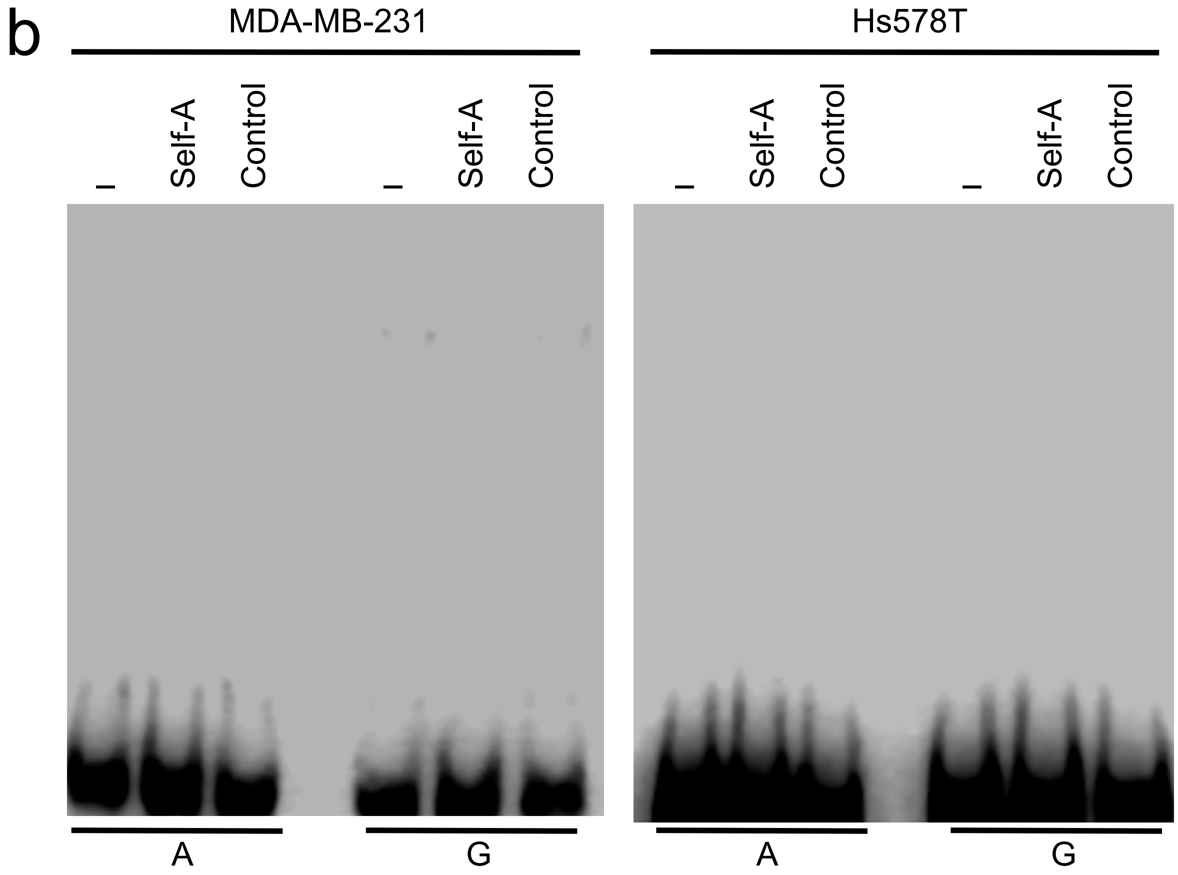

Supplementary Figure 9. EMSAs for oligonucleotides containing SNP rs10941679. The A=common allele and G=minor allele as indicated below the panel, assayed using **(a)** ER^{+}/PR^{-} MDA-MB-361, ER^{+}/PR^{+} MCF7 and **(b)** ER^{-}/PR^{-} MDA-MB-231 and Hs578T nuclear extracts. Primers are listed in **Table S8**. Labels above each lane indicate inclusion of competitor oligonucleotides at 100-fold molar excess, respectively: (-) no competitor and control denotes a non-specific competitor. Red arrowheads show bands of different mobility detected between the A and G alleles.

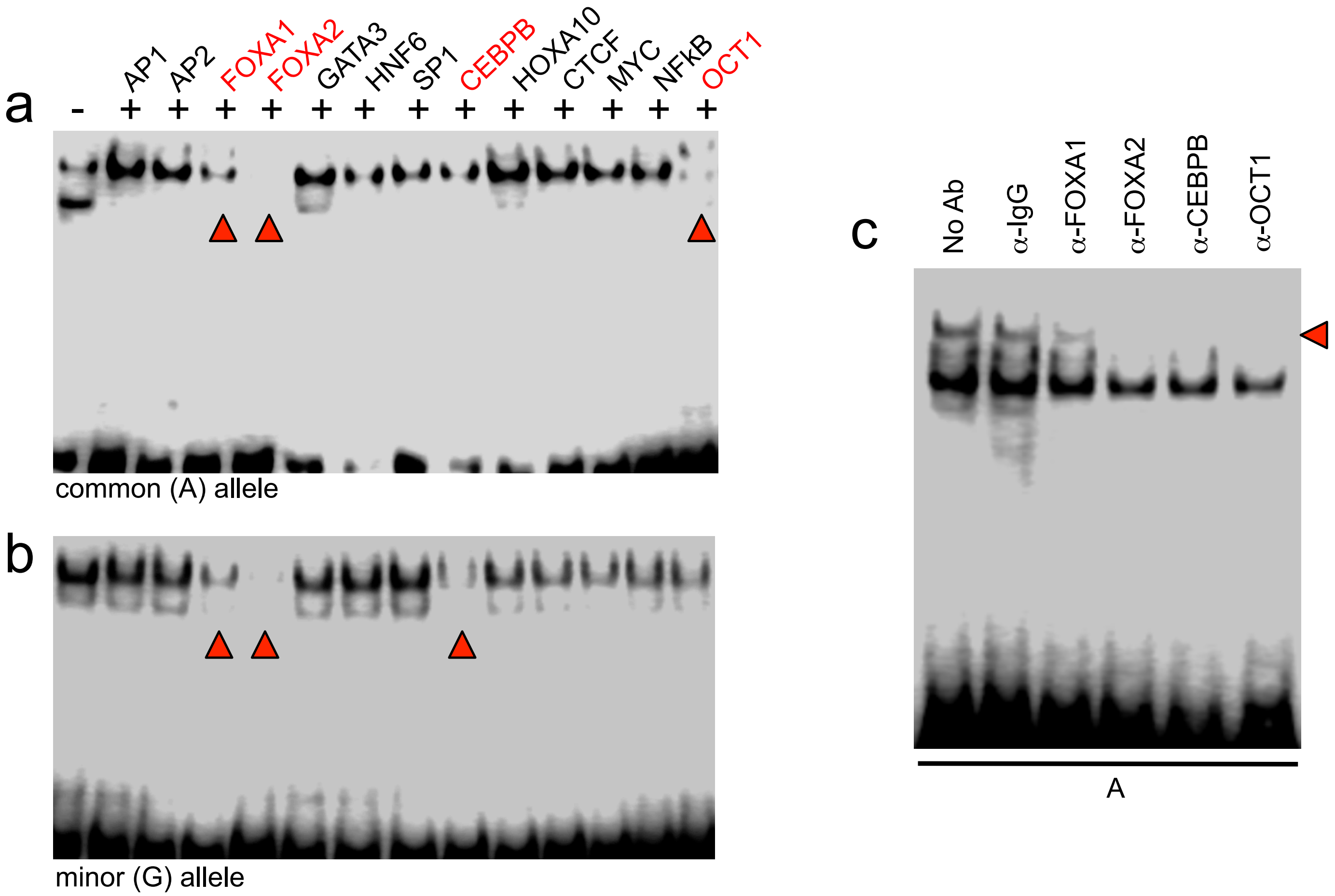

Supplementary Figure 10. Competition EMSAs for SNP rs10941679 to identify candidate nuclear proteins. Competitor oligonucleotides for predicted transcription factors (100-fold molar excess) were incubated with ER^+ BT474 nuclear extracts (competitor sequences are listed in **Table S9**). Red arrowheads indicate bands that were competed for complex formation on either the **(a)** common or **(b)** minor alleles. **(c)** EMSA-supershift using the common DNA duplex and 2 μg of polyclonal antibody against FOXA1 (ab5089, abcam), FOXA2 (sc-20692, Santa Cruz Biotechnology), CEBPB (sc-150) or OCT1 (sc-232) with BT474 nuclear extracts. Rabbit IgG (sc-2027) was used as a negative control. The red arrowhead denotes supershifted complexes.

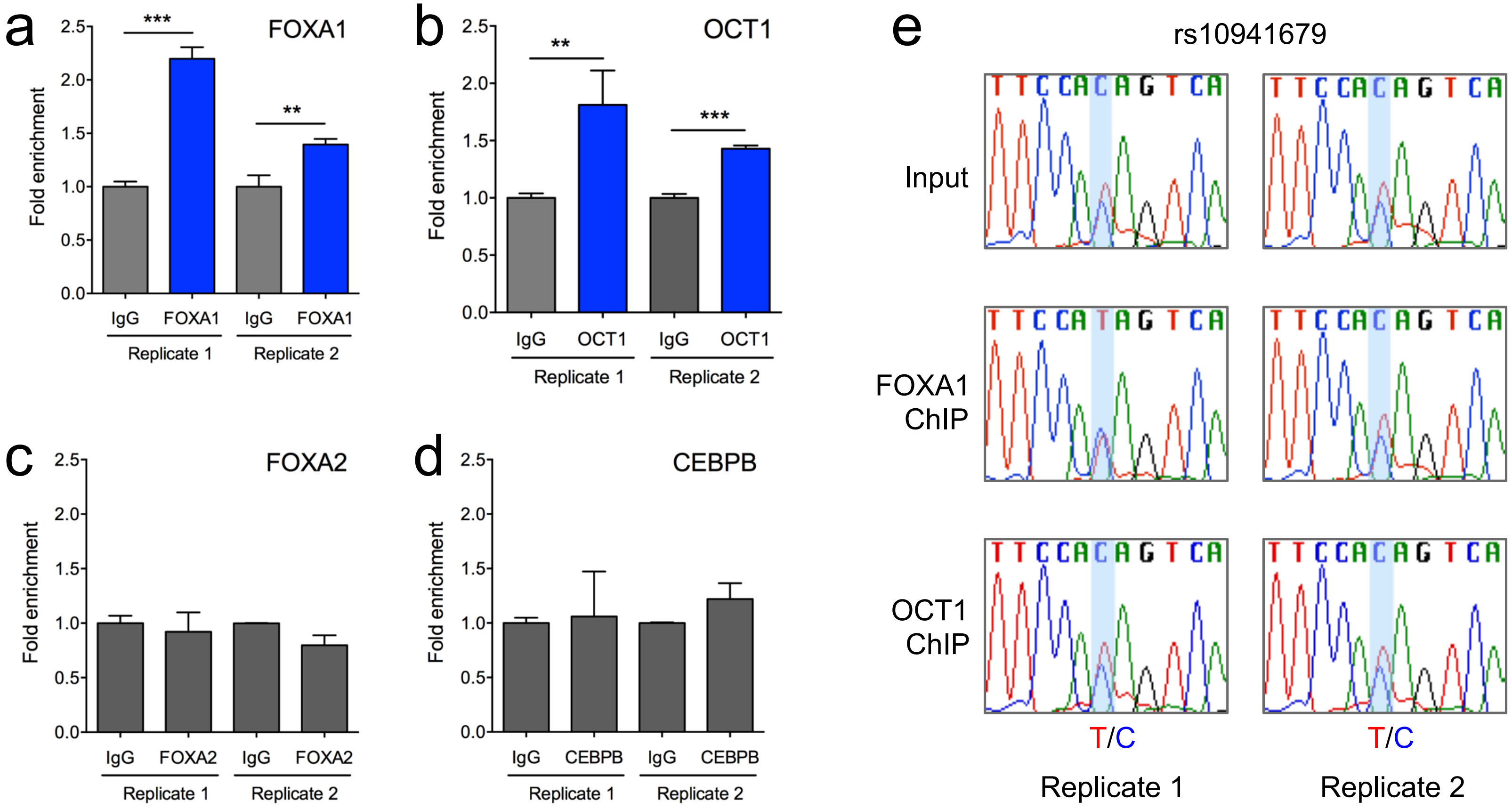

Supplementary Figure 11. FOXA1 and OCT1 binding *in vivo*. ChIP-qPCR results against **(a)** FOXA1, **(b)** OCT1, **(c)** FOXA2 or **(d)** CEBPB in heterozygous BT474 breast cancer cells. Error bars denote SD (n=2). A region within the second intron of *ESR1* served as a negative (Neg) control. *P*-values were determined by two-tailed t-t test. **P<0.01, ***P<0.001. **(e)** Sanger sequencing of the PCR fragment generated using primers flanking SNP rs10941679 following ChIP-qPCR. Primers are listed in **Table S10**.

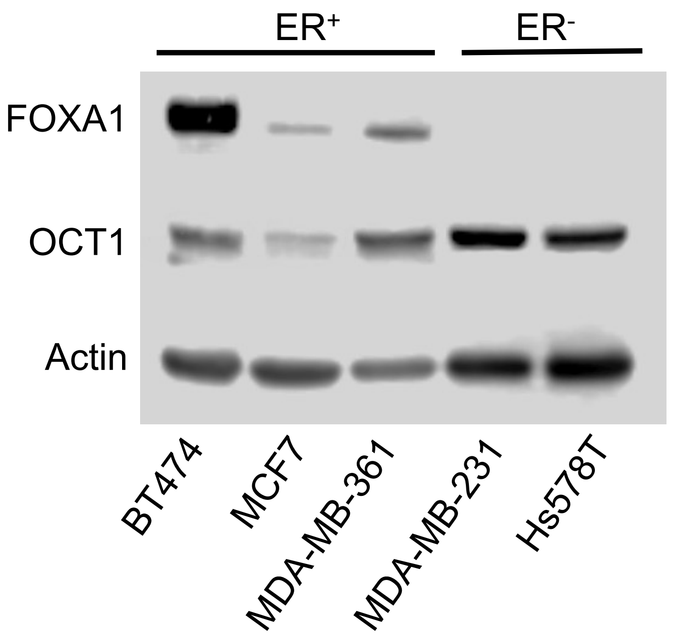

Supplementary Figure 12. Western blot analysis of FOXA1 and OCT1 protein expression in breast cancer cell lines. Actin (Sigma A2066) served as a loading control.

Supplementary Table 1. Strongest Associated SNPs (n=461 *P*<10-4) with overall breast cancer risk from 41 European BCAC studies (n=104,660). * Type refers to either genotyped or imputed SNPs.

SNP	position	Ref	Alt	EAF	OR overall	Pvalue overall	OR ER+	Pvalue ER+	OR ER-	Pvalue ER-	Type	R^2
rs10941679	44706498	A	G	0.27	1.12	2.55E-26	1.15	2.44E-29	1.04	0.08	Gen	0.99
rs10462082	44800917	A	C	0.50	1.1	9.11E-22	1.11	3.40E-20	1.06	0.00	Imp	0.95
c5_pos44911206	44875449	A	G	0.48	0.91	1.67E-21	0.90	3.28E-21	0.95	0.01	Gen	1.00
rs6451770	44691395	T	G	0.39	1.1	2.50E-21	1.13	4.76E-25	1.01	0.59	Gen	1.00
rs7716600	44875005	A	C	0.77	0.9	4.13E-21	0.87	3.17E-24	0.96	0.09	Gen	1.00
rs930395	44822458	G	A	0.23	1.11	7.58E-21	1.14	4.87E-24	1.04	0.08	Gen	1.00
rs13185555	44690964	G	A	0.39	1.1	7.59E-21	1.13	5.36E-25	1.01	0.75	Imp	0.98
rs76514876	44673354	A	C	0.41	1.1	4.91E-20	1.12	5.39E-23	1.01	0.72	Imp	0.98
rs4463188	44642670	T	C	0.41	1.09	5.35E-20	1.12	8.29E-23	1.00	0.80	Gen	1.00
rs4321755	44646195	C	T	0.41	1.09	6.44E-20	1.12	6.75E-23	1.00	0.81	Gen	1.00
rs4339357	44670741	T	C	0.40	1.1	6.64E-20	1.12	9.24E-23	1.01	0.56	Imp	0.97
rs4479849	44644006	G	A	0.41	1.09	6.93E-20	1.12	4.92E-23	1.00	0.85	Gen	1.00
rs12187196	44683819	A	C	0.41	1.09	7.47E-20	1.12	8.48E-23	1.01	0.62	Gen	1.00
rs36068815	44645998	T	C	0.41	1.09	7.80E-20	1.12	5.15E-23	1.00	0.84	Gen	1.00
rs13155752	44680687	A	C	0.41	1.09	1.65E-19	1.12	7.46E-23	1.01	0.71	Imp	0.98
rs13156283	44680758	G	C	0.41	1.09	1.66E-19	1.12	7.48E-23	1.01	0.71	Imp	0.98
rs4492118	44646625	G	A	0.41	1.09	1.86E-19	1.12	1.98E-22	1.01	0.79	Gen	1.00
rs7735881	44650176	A	G	0.41	1.09	1.90E-19	1.12	1.15E-22	1.00	0.82	Gen	1.00
rs12516900	44644495	A	G	0.41	1.09	2.27E-19	1.12	2.34E-22	1.01	0.78	Gen	1.00
rs6861560	44708378	C	G	0.41	1.09	2.31E-19	1.12	7.82E-23	1.01	0.71	Imp	0.98
rs1821936	44699482	A	C	0.41	1.09	2.42E-19	1.12	1.30E-22	1.01	0.67	Imp	0.99
chr5:44648592:I	44648592	C	CTAT	0.41	1.09	2.42E-19	1.12	2.22E-22	1.01	0.76	Imp	0.98
chr5:44645330:D	44645330	AG	A	0.40	1.1	2.50E-19	1.12	3.61E-22	1.01	0.77	Imp	0.95
rs6874055	44666965	T	A	0.41	1.09	2.51E-19	1.12	1.70E-22	1.01	0.75	Imp	0.99
rs12522626	44685698	G	T	0.41	1.09	2.67E-19	1.12	1.73E-22	1.01	0.69	Gen	1.00
rs10805686	44662128	T	C	0.41	1.09	2.95E-19	1.12	2.24E-22	1.01	0.76	Gen	1.00
rs6890556	44648666	C	G	0.41	1.09	3.01E-19	1.12	3.17E-22	1.01	0.76	Gen	1.00
rs2165010	44706780	A	C	0.41	1.09	3.12E-19	1.12	1.68E-22	1.01	0.68	Imp	0.99
rs13176502	44697908	T	C	0.41	1.09	3.36E-19	1.12	2.17E-22	1.01	0.66	Imp	0.99
rs714130	44701418	T	C	0.41	1.09	3.55E-19	1.12	2.03E-22	1.01	0.68	Gen	1.00
rs1438825	44706931	G	A	0.41	1.09	3.58E-19	1.12	1.63E-22	1.01	0.72	Gen	1.00
rs10941677	44662399	G	A	0.41	1.09	3.64E-19	1.12	2.24E-22	1.01	0.77	Gen	1.00
rs1371027	44699857	T	C	0.41	1.09	3.65E-19	1.12	1.90E-22	1.01	0.69	Gen	1.00
rs2218081	44705140	C	T	0.41	1.09	3.76E-19	1.12	1.50E-22	1.01	0.74	Gen	1.00
rs4415084	44662515	C	T	0.41	1.09	3.81E-19	1.12	2.67E-22	1.01	0.78	Gen	1.00

rs1898701	44695429	A	G	0.41	1.09	3.87E-19	1.12	2.53E-22	1.01	0.67	Imp	0.99
rs2165009	44697916	C	T	0.41	1.09	4.09E-19	1.12	1.53E-22	1.01	0.72	Gen	0.99
rs4415085	44662959	C	G	0.41	1.09	4.21E-19	1.12	2.45E-22	1.00	0.82	Gen	1.00
rs6871484	44676305	T	C	0.41	1.09	4.24E-19	1.12	2.26E-22	1.01	0.75	Imp	0.99
rs920329	44702507	T	C	0.41	1.09	4.54E-19	1.12	1.71E-22	1.01	0.75	Gen	1.00
rs6863598	44668623	C	T	0.41	1.09	4.62E-19	1.12	2.67E-22	1.01	0.76	Imp	0.98
rs4419600	44678534	T	G	0.41	1.09	4.72E-19	1.12	4.27E-22	1.01	0.74	Gen	1.00
rs34762678	44705514	T	C	0.41	1.09	4.75E-19	1.12	2.22E-22	1.01	0.67	Gen	1.00
rs4571480	44687188	C	T	0.41	1.09	4.85E-19	1.12	1.64E-22	1.01	0.77	Gen	1.00
rs13156930	44698035	G	C	0.41	1.09	4.92E-19	1.12	1.76E-22	1.01	0.75	Gen	1.00
c5_pos44711954	44676197	C	T	0.41	1.09	5.20E-19	1.12	3.06E-22	1.00	0.80	Gen	1.00
chr5:44637587:I	44637587	T	TC	0.40	1.09	5.28E-19	1.12	2.86E-22	1.00	0.87	Imp	0.97
chr5:44929230:D	44929230	ATAATAT	A	0.60	0.91	5.60E-19	0.89	4.32E-21	0.98	0.44	Imp	0.92
rs10462079	44700110	G	T	0.41	1.09	5.77E-19	1.12	3.09E-22	1.01	0.69	Gen	0.99
chr5:44678911:D	44678911	TA	T	0.40	1.09	5.83E-19	1.12	3.22E-22	1.01	0.79	Imp	0.97
rs2218080	44714330	T	C	0.42	1.09	5.99E-19	1.12	2.50E-22	1.01	0.76	Imp	0.96
rs2013513	44702306	G	A	0.41	1.09	6.02E-19	1.12	3.39E-22	1.01	0.68	Imp	0.99
rs13179137	44702692	C	T	0.41	1.09	6.55E-19	1.12	2.82E-22	1.01	0.69	Gen	1.00
rs7720551	44664477	C	T	0.41	1.09	7.05E-19	1.12	4.87E-22	1.00	0.81	Imp	0.99
rs10941678	44672783	C	A	0.38	1.1	7.18E-19	1.13	7.22E-22	1.00	0.98	Imp	0.89
rs4607355	44868070	A	G	0.52	0.91	7.56E-19	0.89	3.46E-20	0.97	0.12	Imp	0.84
rs144629423	44669403	G	T	0.41	1.09	8.10E-19	1.12	4.28E-22	1.01	0.75	Imp	0.97
rs6884702	44682589	A	G	0.40	1.09	8.55E-19	1.12	3.18E-22	1.01	0.75	Imp	0.99
rs7723539	44660210	G	C	0.41	1.09	8.60E-19	1.12	5.63E-22	1.00	0.84	Imp	0.99
rs10805685	44661958	C	G	0.41	1.09	9.02E-19	1.12	5.83E-22	1.00	0.84	Imp	0.99
rs4571481	44687433	C	T	0.39	1.09	9.77E-19	1.11	4.21E-21	1.03	0.19	Gen	0.95
rs12515012	44694535	G	A	0.40	1.09	9.84E-19	1.12	4.89E-22	1.01	0.75	Imp	0.98
rs181516187	44672104	C	T	0.40	1.09	1.11E-18	1.12	6.79E-22	1.00	0.82	Imp	0.97
rs16901937	44709141	A	G	0.41	1.09	1.71E-18	1.12	7.12E-22	1.01	0.79	Gen	1.00
rs7701466	44663137	C	T	0.40	1.09	3.42E-18	1.12	1.08E-21	1.00	1.00	Imp	0.97
rs11949847	44752169	A	G	0.42	1.09	3.92E-18	1.11	1.08E-20	1.00	0.81	Gen	0.99
c5_pos44685154	44649397	A	C	0.38	1.09	4.42E-18	1.11	1.23E-20	1.02	0.32	Gen	0.98
rs56248730	44681267	A	G	0.38	1.09	4.64E-18	1.12	9.44E-21	1.02	0.32	Imp	0.98
c5_pos44720000	44684243	A	G	0.38	1.09	4.65E-18	1.12	8.11E-21	1.02	0.32	Gen	1.00
rs6867533	44827292	G	T	0.40	1.09	5.17E-18	1.11	2.05E-20	1.00	0.84	Gen	0.96
rs994793	44743247	A	G	0.42	1.09	5.41E-18	1.11	1.04E-20	1.00	0.82	Gen	1.00
rs920328	44699051	G	A	0.38	1.09	6.04E-18	1.12	7.48E-21	1.02	0.31	Gen	1.00
rs11746980	44777878	G	A	0.42	1.09	6.64E-18	1.11	2.04E-20	1.00	0.80	Gen	1.00
rs2330572	44740989	A	C	0.42	1.09	7.56E-18	1.11	1.25E-20	1.00	0.84	Gen	1.00
rs7737491	44790842	A	G	0.37	1.09	8.66E-18	1.11	4.58E-19	1.03	0.20	Imp	0.96
rs10447145	44689796	T	G	0.38	1.09	8.76E-18	1.11	1.33E-20	1.02	0.34	Imp	0.98

rs145342568	44672237	A	G	0.39	1.09	1.00E-17	1.12	8.13E-21	1.01	0.69	Imp	0.91
rs13160259	44828964	G	C	0.42	1.09	1.17E-17	1.11	5.45E-20	1.01	0.77	Imp	0.98
rs10072259	44834791	T	C	0.61	0.92	1.22E-17	0.90	1.39E-19	0.98	0.35	Imp	0.98
rs10512865	44823367	C	T	0.41	1.09	1.27E-17	1.11	6.61E-20	1.01	0.77	Imp	0.99
rs11958808	44945090	G	C	0.41	1.09	1.36E-17	1.11	1.25E-19	1.01	0.76	Gen	1.00
rs4866922	44932505	C	G	0.59	0.92	1.41E-17	0.90	1.11E-19	0.99	0.72	Imp	0.99
chr5:44837254:I	44837254	T	TA	0.58	0.92	1.43E-17	0.90	7.54E-20	0.99	0.77	Imp	0.99
rs1048758	44828594	G	A	0.41	1.09	1.43E-17	1.11	6.71E-20	1.00	0.80	Gen	1.00
rs1371023	44846575	C	T	0.59	0.92	1.52E-17	0.90	8.79E-20	1.00	0.79	Gen	1.00
rs10054773	44930234	C	G	0.59	0.92	1.54E-17	0.90	1.30E-19	0.99	0.71	Imp	0.98
rs10044096	44927365	A	C	0.59	0.92	1.55E-17	0.90	1.49E-19	0.99	0.71	Imp	0.99
rs7716571	44816984	G	A	0.42	1.09	1.62E-17	1.11	1.20E-19	1.01	0.78	Gen	0.96
chr5:44884873:D	44884873	TAAAC	T	0.58	0.92	1.73E-17	0.90	1.36E-19	0.99	0.73	Imp	0.98
rs2118764	44787723	C	T	0.41	1.09	1.77E-17	1.11	1.40E-19	1.00	0.80	Imp	0.98
rs10064434	44834702	G	A	0.58	0.92	1.81E-17	0.90	7.93E-20	1.00	0.81	Imp	0.98
rs930396	44856451	T	G	0.59	0.92	1.89E-17	0.90	1.58E-19	0.99	0.79	Gen	1.00
rs7380559	44837010	A	T	0.59	0.92	1.91E-17	0.90	1.02E-19	1.00	0.80	Gen	1.00
rs1821934	44789172	T	C	0.41	1.09	1.91E-17	1.11	1.63E-19	1.01	0.74	Imp	0.98
rs7711697	44780403	A	T	0.41	1.09	1.95E-17	1.11	1.24E-19	1.00	0.82	Gen	1.00
rs7711136	44823079	G	C	0.41	1.09	1.96E-17	1.11	1.10E-19	1.00	0.79	Gen	0.99
rs1438820	44797770	G	A	0.41	1.09	2.03E-17	1.11	1.20E-19	1.00	0.84	Gen	1.00
rs4866923	44932641	A	C	0.58	0.92	2.07E-17	0.90	2.19E-19	0.99	0.74	Imp	0.97
rs6896417	44802638	A	G	0.41	1.09	2.15E-17	1.11	1.56E-19	1.00	0.84	Gen	1.00
rs1061310	44820850	G	C	0.41	1.09	2.15E-17	1.11	1.16E-19	1.01	0.79	Gen	1.00
rs1438821	44858451	G	A	0.59	0.92	2.17E-17	0.90	1.82E-19	1.00	0.80	Gen	1.00
rs13177711	44796962	A	T	0.41	1.09	2.23E-17	1.11	1.69E-19	1.00	0.83	Gen	0.99
rs4629607	44857467	G	T	0.58	0.92	2.23E-17	0.90	6.97E-18	0.97	0.11	Imp	0.90
rs13159598	44805926	A	G	0.41	1.09	2.27E-17	1.11	1.61E-19	1.00	0.83	Gen	1.00
rs7730841	44823134	T	C	0.42	1.09	2.32E-17	1.11	1.24E-19	1.00	0.80	Gen	0.98
rs13362132	44858260	T	G	0.59	0.92	2.54E-17	0.90	2.03E-19	1.00	0.79	Gen	1.00
rs10043344	44926518	A	T	0.59	0.92	2.57E-17	0.90	1.50E-19	0.99	0.76	Gen	1.00
rs187822318	44671468	C	G	0.35	1.1	2.71E-17	1.12	2.72E-19	1.02	0.28	Imp	0.84
rs7720787	44817309	G	A	0.41	1.09	2.88E-17	1.11	1.52E-19	1.00	0.82	Gen	1.00
rs7705343	44879577	G	A	0.59	0.92	3.06E-17	0.90	2.70E-19	0.99	0.79	Gen	1.00
rs149708574	44825919	T	A	0.30	1.1	3.10E-17	1.12	2.35E-18	1.04	0.07	Imp	0.84
rs2877172	44790567	A	G	0.41	1.09	3.13E-17	1.11	2.26E-19	1.00	0.83	Imp	0.97
rs4518409	44870852	T	C	0.59	0.92	3.18E-17	0.90	2.75E-19	1.00	0.83	Gen	1.00
rs10077814	44916789	C	T	0.59	0.92	3.39E-17	0.90	1.88E-19	1.00	0.80	Gen	1.00
rs4329028	44872353	T	A	0.59	0.92	3.64E-17	0.90	4.23E-19	0.99	0.76	Gen	1.00
chr5:44921964:I	44921964	A	AT	0.59	0.92	4.14E-17	0.90	3.42E-19	0.99	0.78	Imp	0.96
c5_pos44933593	44897836	T	C	0.59	0.92	4.45E-17	0.90	2.78E-19	0.99	0.78	Gen	0.99

rs9292915	44871657	C	T	0.59	0.92	4.49E-17	0.90	4.55E-19	0.99	0.78	Gen	1.00
rs9292913	44870879	G	A	0.59	0.92	4.63E-17	0.90	4.50E-19	1.00	0.80	Gen	1.00
rs4412123	44876288	T	C	0.59	0.92	4.79E-17	0.90	4.15E-19	1.00	0.86	Gen	1.00
rs9790896	44900091	G	A	0.59	0.92	5.21E-17	0.90	2.76E-19	1.00	0.86	Gen	1.00
chr5:44818855:D	44818855	CCAA	C	0.38	1.09	5.37E-17	1.11	1.48E-18	1.02	0.26	Imp	0.99
rs9790879	44899885	C	T	0.59	0.92	5.65E-17	0.90	2.82E-19	1.00	0.86	Gen	1.00
rs11957920	44905086	T	C	0.59	0.92	5.72E-17	0.90	4.80E-19	1.00	0.86	Gen	1.00
rs10070339	44907882	G	A	0.59	0.92	6.40E-17	0.90	4.39E-19	1.00	0.90	Gen	1.00
rs7726586	44848822	C	T	0.62	0.92	9.03E-17	0.90	1.89E-18	0.98	0.33	Gen	1.00
rs13154781	44775027	C	T	0.39	1.09	9.35E-17	1.11	9.73E-19	1.02	0.35	Imp	0.99
rs1438827	44751956	C	A	0.39	1.09	9.41E-17	1.11	7.00E-19	1.02	0.35	Gen	1.00
rs7712949	44770345	C	T	0.39	1.09	9.90E-17	1.11	7.94E-19	1.02	0.37	Gen	1.00
chr5:44810239:D	44810239	TG	T	0.41	1.09	1.01E-16	1.11	6.06E-19	1.01	0.74	Imp	0.96
rs7708213	44848401	T	C	0.62	0.92	1.03E-16	0.90	2.52E-18	0.98	0.30	Gen	1.00
rs34237302	44852862	C	A	0.61	0.92	1.04E-16	0.90	2.61E-18	0.98	0.29	Gen	0.99
rs4440370	44853352	A	G	0.62	0.92	1.10E-16	0.90	2.63E-18	0.98	0.31	Gen	1.00
rs11747159	44737710	C	T	0.39	1.09	1.12E-16	1.11	1.12E-18	1.02	0.34	Gen	1.00
rs4492119	44855614	A	G	0.61	0.92	1.37E-16	0.90	2.83E-18	0.98	0.33	Gen	0.98
rs1371022	44847992	T	A	0.62	0.92	1.42E-16	0.90	3.56E-18	0.98	0.32	Gen	1.00
rs987394	44846378	A	G	0.62	0.92	1.49E-16	0.90	3.64E-18	0.98	0.31	Gen	1.00
rs10462081	44800665	G	A	0.38	1.09	1.58E-16	1.11	4.22E-18	1.02	0.33	Gen	1.00
rs1438819	44797846	T	A	0.38	1.09	1.60E-16	1.11	4.54E-18	1.02	0.34	Gen	1.00
rs7715731	44846844	A	C	0.62	0.92	1.69E-16	0.90	3.01E-18	0.98	0.33	Gen	1.00
rs1371025	44834233	G	A	0.62	0.92	1.70E-16	0.90	3.46E-18	0.98	0.32	Gen	0.99
rs1866406	44809945	G	C	0.39	1.09	1.74E-16	1.11	4.31E-18	1.02	0.32	Gen	0.99
rs9637797	44818181	A	G	0.39	1.09	1.74E-16	1.11	3.90E-18	1.02	0.32	Gen	1.00
rs930394	44822617	G	A	0.38	1.09	1.83E-16	1.11	3.68E-18	1.02	0.32	Gen	1.00
rs3761650	44808356	G	A	0.38	1.09	1.83E-16	1.11	4.39E-18	1.02	0.33	Gen	1.00
rs6451781	44860506	T	C	0.61	0.92	1.86E-16	0.90	6.29E-18	0.98	0.31	Gen	1.00
rs10462080	44799052	C	A	0.38	1.09	1.90E-16	1.11	5.95E-18	1.02	0.34	Imp	0.98
rs71610376	44825023	G	C	0.38	1.09	1.92E-16	1.11	3.97E-18	1.02	0.33	Imp	0.99
rs12517690	44939293	G	A	0.39	1.09	1.93E-16	1.11	5.48E-18	1.02	0.32	Gen	1.00
chr5:44816067:D	44816067	TAAAAG	T	0.38	1.09	1.96E-16	1.11	4.43E-18	1.02	0.33	Imp	0.99
rs727305	44796042	T	C	0.39	1.09	1.97E-16	1.11	4.67E-18	1.02	0.35	Gen	0.99
rs7703497	44857028	A	G	0.61	0.92	1.97E-16	0.90	5.87E-18	0.98	0.34	Gen	1.00
rs4457089	44821736	C	T	0.38	1.09	2.02E-16	1.11	4.19E-18	1.02	0.32	Gen	1.00
rs1371024	44843590	C	T	0.61	0.92	2.04E-16	0.90	4.26E-18	0.98	0.32	Gen	1.00
rs727304	44796290	G	A	0.38	1.09	2.05E-16	1.11	5.92E-18	1.02	0.34	Gen	1.00
rs10038562	44832226	C	T	0.61	0.92	2.05E-16	0.90	4.15E-18	0.98	0.32	Gen	0.99
rs13174122	44810740	T	C	0.39	1.09	2.06E-16	1.11	4.57E-18	1.02	0.32	Gen	0.99
rs7703059	44826436	G	A	0.38	1.09	2.06E-16	1.11	4.34E-18	1.02	0.34	Imp	0.99

rs2165008	44821679	G	A	0.38	1.09	2.08E-16	1.11	4.35E-18	1.02	0.32	Gen	1.00
rs7717459	44804525	C	T	0.38	1.09	2.12E-16	1.11	5.66E-18	1.02	0.34	Gen	1.00
rs4298259	44920711	G	A	0.61	0.92	2.12E-16	0.90	4.29E-18	0.98	0.33	Gen	1.00
rs11746506	44812566	C	T	0.38	1.09	2.13E-16	1.11	4.36E-18	1.02	0.32	Gen	1.00
rs12513749	44828203	G	A	0.39	1.09	2.16E-16	1.11	4.46E-18	1.02	0.34	Imp	0.99
rs13189120	44822283	A	T	0.39	1.09	2.17E-16	1.11	4.55E-18	1.02	0.32	Gen	1.00
rs6868232	44827680	G	C	0.38	1.09	2.18E-16	1.11	4.19E-18	1.02	0.34	Imp	0.99
rs16901989	44837129	C	T	0.61	0.92	2.18E-16	0.90	4.61E-18	0.98	0.32	Gen	1.00
rs13155698	44828681	A	C	0.39	1.09	2.18E-16	1.11	3.97E-18	1.02	0.33	Gen	1.00
rs6896350	44832571	C	A	0.61	0.92	2.19E-16	0.90	4.25E-18	0.98	0.33	Gen	1.00
rs1438822	44859172	C	G	0.61	0.92	2.21E-16	0.90	7.25E-18	0.98	0.32	Gen	1.00
rs729599	44842260	G	A	0.62	0.92	2.23E-16	0.90	3.96E-18	0.98	0.33	Imp	0.99
rs6451772	44785763	A	C	0.39	1.09	2.24E-16	1.11	6.13E-18	1.02	0.37	Imp	0.98
rs6872254	44803784	C	T	0.39	1.09	2.28E-16	1.11	6.90E-18	1.02	0.34	Gen	0.99
rs1837286	44858741	C	T	0.62	0.92	2.28E-16	0.90	6.49E-18	0.98	0.32	Imp	0.99
rs4373287	44862884	G	T	0.61	0.92	2.28E-16	0.90	7.68E-18	0.98	0.33	Imp	0.99
rs11741772	44814597	A	C	0.38	1.09	2.29E-16	1.11	5.23E-18	1.02	0.33	Imp	0.99
rs3761648	44808079	A	G	0.38	1.09	2.31E-16	1.11	5.47E-18	1.02	0.34	Imp	0.99
rs16901964	44783255	C	T	0.38	1.09	2.33E-16	1.11	5.86E-18	1.02	0.35	Gen	1.00
rs12651949	44798112	C	T	0.38	1.09	2.33E-16	1.11	6.20E-18	1.02	0.34	Gen	1.00
rs10462083	44806737	T	G	0.38	1.09	2.33E-16	1.11	5.65E-18	1.02	0.35	Imp	0.99
rs4596389	44836556	A	C	0.62	0.92	2.35E-16	0.90	3.59E-18	0.98	0.33	Gen	1.00
rs969679	44804052	T	C	0.38	1.09	2.35E-16	1.11	5.95E-18	1.02	0.34	Imp	0.99
rs16901990	44842740	A	C	0.62	0.92	2.35E-16	0.90	3.54E-18	0.98	0.33	Gen	1.00
rs7707315	44918805	T	C	0.61	0.92	2.38E-16	0.90	4.48E-18	0.98	0.35	Gen	1.00
rs16901965	44783358	G	A	0.38	1.09	2.39E-16	1.11	5.51E-18	1.02	0.35	Gen	1.00
rs3761649	44808164	T	C	0.38	1.09	2.40E-16	1.11	5.60E-18	1.02	0.34	Imp	0.99
rs16901963	44783102	T	A	0.39	1.09	2.40E-16	1.11	5.55E-18	1.02	0.35	Gen	1.00
rs6451775	44836788	G	A	0.62	0.92	2.41E-16	0.90	4.60E-18	0.98	0.33	Imp	0.99
rs12518851	44828231	A	G	0.38	1.09	2.44E-16	1.11	4.45E-18	1.02	0.34	Imp	0.99
rs9637796	44817919	G	A	0.38	1.09	2.45E-16	1.11	5.69E-18	1.02	0.33	Imp	0.99
rs7736952	44790379	C	T	0.39	1.09	2.49E-16	1.11	6.96E-18	1.02	0.35	Imp	0.99
rs6451783	44918293	G	A	0.61	0.92	2.51E-16	0.90	4.43E-18	0.98	0.35	Gen	1.00
rs6893319	44863729	T	G	0.62	0.92	2.53E-16	0.90	7.70E-18	0.98	0.34	Imp	0.99
rs2083243	44840147	C	T	0.62	0.92	2.55E-16	0.90	4.35E-18	0.98	0.33	Imp	0.99
rs12188871	44814004	G	A	0.39	1.09	2.55E-16	1.11	5.73E-18	1.02	0.32	Gen	1.00
rs13185174	44791791	G	A	0.38	1.09	2.56E-16	1.11	5.98E-18	1.02	0.36	Imp	0.98
rs10041518	44927406	T	C	0.61	0.92	2.57E-16	0.90	6.23E-18	0.98	0.34	Gen	1.00
rs2330620	44839822	G	A	0.62	0.92	2.58E-16	0.90	4.62E-18	0.98	0.33	Imp	0.99
rs4604199	44855716	T	C	0.62	0.92	2.59E-16	0.90	6.78E-18	0.98	0.34	Imp	0.99
rs4605791	44855712	T	G	0.62	0.92	2.60E-16	0.90	6.78E-18	0.98	0.34	Imp	0.99

rs1837285	44858490	T	G	0.62	0.92	2.60E-16	0.90	6.52E-18	0.98	0.33	Imp	0.99
rs3747479	44809162	G	C	0.39	1.09	2.60E-16	1.11	5.48E-18	1.02	0.34	Gen	1.00
rs6871052	44863317	C	T	0.62	0.92	2.63E-16	0.90	7.44E-18	0.98	0.34	Gen	1.00
rs6451784	44922331	A	G	0.58	0.92	2.63E-16	0.90	3.17E-18	0.98	0.34	Imp	0.90
rs7728431	44922679	T	C	0.61	0.92	2.65E-16	0.90	5.40E-18	0.98	0.34	Gen	1.00
rs4566804	44855739	T	C	0.62	0.92	2.66E-16	0.90	6.87E-18	0.98	0.34	Imp	0.99
rs7736092	44920995	C	T	0.61	0.92	2.66E-16	0.90	5.15E-18	0.98	0.33	Gen	1.00
rs10064437	44834721	G	C	0.61	0.92	2.69E-16	0.90	3.66E-18	0.98	0.37	Imp	0.98
rs10070928	44888557	C	G	0.60	0.92	2.72E-16	0.91	9.63E-18	0.98	0.31	Gen	0.96
rs7710952	44785791	C	T	0.38	1.09	2.74E-16	1.11	7.59E-18	1.02	0.35	Imp	0.99
rs7710978	44785864	A	G	0.38	1.09	2.75E-16	1.11	7.62E-18	1.02	0.35	Imp	0.99
rs10053247	44863959	C	T	0.62	0.92	2.78E-16	0.90	8.60E-18	0.98	0.34	Imp	0.99
rs12656984	44824702	A	G	0.38	1.09	2.79E-16	1.11	6.67E-18	1.02	0.32	Imp	0.98
rs7703618	44914579	G	A	0.61	0.92	2.83E-16	0.90	7.58E-18	0.98	0.34	Gen	1.00
rs10044321	44890760	G	A	0.61	0.92	2.85E-16	0.91	8.30E-18	0.98	0.35	Imp	0.99
rs10073945	44932134	A	G	0.61	0.92	2.86E-16	0.90	6.86E-18	0.98	0.35	Imp	0.99
chr5:44916609:D	44916609	GT	G	0.63	0.92	2.92E-16	0.90	4.50E-18	0.98	0.33	Imp	0.96
rs13179835	44908703	G	A	0.61	0.92	2.94E-16	0.90	5.90E-18	0.99	0.45	Imp	0.97
rs6871820	44919557	T	C	0.61	0.92	2.99E-16	0.90	6.36E-18	0.98	0.33	Gen	0.99
rs10038554	44927107	G	A	0.61	0.92	2.99E-16	0.90	6.52E-18	0.98	0.34	Gen	1.00
rs34325259	44936860	G	T	0.38	1.08	3.02E-16	1.10	9.54E-18	1.02	0.42	Gen	0.96
rs13183209	44803749	G	A	0.38	1.09	3.04E-16	1.11	7.72E-18	1.02	0.35	Imp	0.99
rs6451774	44793984	G	C	0.38	1.09	3.04E-16	1.10	8.79E-18	1.02	0.35	Imp	0.99
rs10063172	44930709	T	C	0.61	0.92	3.11E-16	0.90	7.55E-18	0.98	0.35	Imp	0.99
rs6451778	44857988	C	T	0.62	0.92	3.12E-16	0.90	8.11E-18	0.98	0.34	Imp	0.99
rs10039866	44925061	T	A	0.61	0.92	3.13E-16	0.90	5.21E-18	0.98	0.35	Gen	1.00
rs11948636	44881229	A	C	0.61	0.92	3.15E-16	0.91	1.00E-17	0.98	0.34	Gen	1.00
rs34115673	44892801	T	G	0.61	0.92	3.20E-16	0.91	8.96E-18	0.98	0.36	Imp	0.98
rs10035358	44892902	C	A	0.61	0.92	3.21E-16	0.91	8.71E-18	0.98	0.36	Imp	0.98
rs10072025	44843494	C	T	0.62	0.92	3.22E-16	0.90	5.21E-18	0.98	0.34	Imp	0.99
rs6875933	44786696	C	T	0.38	1.09	3.26E-16	1.11	6.99E-18	1.02	0.35	Imp	0.97
rs11750119	44786141	G	T	0.38	1.09	3.29E-16	1.11	6.37E-18	1.02	0.35	Imp	0.99
rs13154729	44892929	C	T	0.61	0.92	3.30E-16	0.91	9.07E-18	0.98	0.36	Imp	0.99
rs10057341	44931840	C	T	0.61	0.92	3.34E-16	0.90	6.56E-18	0.98	0.36	Gen	1.00
chr5:44904186:D	44904186	CACTTA	C	0.59	0.92	3.37E-16	0.90	1.97E-17	0.98	0.43	Imp	0.94
rs13153556	44928101	G	A	0.61	0.92	3.40E-16	0.90	6.16E-18	0.98	0.36	Gen	1.00
rs10059086	44872007	C	T	0.61	0.92	3.42E-16	0.91	1.13E-17	0.98	0.34	Gen	1.00
rs2330619	44796062	T	C	0.38	1.08	3.43E-16	1.10	9.62E-18	1.02	0.36	Imp	0.99
rs13168400	44883536	C	T	0.61	0.92	3.45E-16	0.91	1.22E-17	0.98	0.36	Imp	0.99
rs67274820	44782295	T	C	0.38	1.08	3.50E-16	1.11	7.18E-18	1.02	0.37	Imp	0.99
chr5:44795637:I	44795637	G	GA	0.38	1.08	3.51E-16	1.11	8.66E-18	1.02	0.36	Imp	0.99

rs10069220	44881978	G	A	0.61	0.92	3.53E-16	0.91	1.05E-17	0.98	0.33	Gen	1.00
rs4495192	44929177	T	C	0.62	0.92	3.56E-16	0.90	5.47E-18	0.98	0.35	Imp	0.99
rs13157608	44881504	C	T	0.61	0.92	3.62E-16	0.91	1.27E-17	0.98	0.36	Imp	0.99
rs10941687	44921183	G	A	0.62	0.92	3.67E-16	0.90	6.65E-18	0.98	0.35	Imp	0.99
rs10040488	44880288	G	T	0.62	0.92	3.69E-16	0.91	1.30E-17	0.98	0.36	Imp	0.99
rs9637783	44819646	T	G	0.39	1.08	3.70E-16	1.11	6.26E-18	1.02	0.37	Gen	1.00
rs4866920	44879146	G	T	0.62	0.92	3.76E-16	0.91	1.35E-17	0.98	0.36	Imp	0.99
rs10473376	44931228	C	T	0.62	0.92	3.79E-16	0.90	7.90E-18	0.98	0.36	Imp	0.99
rs4591754	44895091	G	A	0.62	0.92	3.80E-16	0.91	9.81E-18	0.98	0.36	Imp	0.99
rs7356604	44895199	T	C	0.62	0.92	3.80E-16	0.91	9.82E-18	0.98	0.36	Imp	0.99
rs12652026	44798609	C	T	0.38	1.08	3.83E-16	1.11	8.81E-18	1.02	0.36	Imp	0.99
rs4642377	44885240	A	T	0.61	0.92	3.84E-16	0.91	1.40E-17	0.98	0.35	Imp	0.99
rs6875287	44941630	C	T	0.39	1.09	3.85E-16	1.10	1.44E-17	1.02	0.32	Imp	0.98
rs10042455	44925018	A	G	0.62	0.92	3.91E-16	0.90	6.70E-18	0.98	0.35	Imp	0.99
rs13187933	44883243	T	C	0.62	0.92	3.93E-16	0.91	1.08E-17	0.98	0.36	Imp	0.99
rs6883465	44891620	T	C	0.62	0.92	4.01E-16	0.91	9.16E-18	0.98	0.37	Imp	0.99
rs4391175	44890056	A	G	0.62	0.92	4.03E-16	0.90	9.00E-18	0.98	0.37	Imp	0.99
rs10070037	44870237	A	T	0.61	0.92	4.07E-16	0.91	1.06E-17	0.98	0.37	Gen	1.00
rs7718354	44891014	T	G	0.62	0.92	4.08E-16	0.91	9.29E-18	0.98	0.37	Imp	0.99
rs7734331	44895588	G	A	0.62	0.92	4.13E-16	0.91	9.36E-18	0.98	0.37	Imp	0.99
rs11948387	44884241	T	C	0.62	0.92	4.16E-16	0.91	1.13E-17	0.98	0.36	Imp	0.99
rs6859157	44783838	G	A	0.38	1.08	4.17E-16	1.11	8.33E-18	1.02	0.37	Imp	0.99
rs11958451	44866902	A	G	0.61	0.92	4.20E-16	0.91	1.53E-17	0.98	0.35	Imp	0.99
rs4323241	44929156	T	C	0.61	0.92	4.28E-16	0.90	1.72E-17	0.98	0.33	Imp	0.97
rs10060878	44899424	T	C	0.61	0.92	4.37E-16	0.91	8.78E-18	0.98	0.36	Gen	1.00
rs6894324	44867336	G	C	0.62	0.92	4.37E-16	0.91	1.38E-17	0.98	0.35	Imp	0.99
rs10040082	44865854	T	C	0.62	0.92	4.43E-16	0.91	1.50E-17	0.98	0.34	Gen	1.00
rs9292914	44871381	A	C	0.61	0.92	4.45E-16	0.91	1.23E-17	0.98	0.37	Gen	1.00
rs10065638	44866162	T	C	0.61	0.92	4.48E-16	0.91	1.39E-17	0.98	0.34	Gen	0.98
rs10473377	44931246	T	C	0.62	0.92	4.49E-16	0.90	8.71E-18	0.98	0.35	Imp	0.99
rs34501299	44913079	T	G	0.61	0.92	4.50E-16	0.91	1.16E-17	0.98	0.38	Gen	1.00
rs4395640	44869100	T	C	0.62	0.92	4.53E-16	0.91	1.47E-17	0.98	0.35	Gen	1.00
rs7708686	44922838	C	G	0.62	0.92	4.69E-16	0.90	7.26E-18	0.98	0.35	Imp	0.99
rs7708506	44922704	C	G	0.62	0.92	4.73E-16	0.90	7.31E-18	0.98	0.35	Imp	0.99
rs12109710	44929928	A	G	0.62	0.92	4.74E-16	0.90	9.42E-18	0.98	0.35	Imp	0.98
rs6868779	44886000	A	G	0.61	0.92	4.80E-16	0.91	1.42E-17	0.98	0.36	Gen	1.00
rs13356086	44887679	T	C	0.62	0.92	4.84E-16	0.91	1.08E-17	0.98	0.37	Imp	0.98
rs11951760	44872172	G	A	0.62	0.92	4.97E-16	0.91	1.18E-17	0.98	0.39	Imp	0.99
rs4866784	44901131	T	C	0.61	0.92	5.05E-16	0.91	1.01E-17	0.98	0.38	Gen	1.00
rs10057521	44865986	C	T	0.60	0.92	5.07E-16	0.91	1.51E-17	0.98	0.36	Gen	0.95
rs4129642	44898129	G	T	0.61	0.92	5.12E-16	0.91	9.43E-18	0.98	0.36	Gen	1.00

rs6881563	44912853	C	T	0.61	0.92	5.28E-16	0.91	1.38E-17	0.98	0.38	Gen	1.00
rs4866783	44876507	C	T	0.62	0.92	5.62E-16	0.91	1.38E-17	0.98	0.39	Imp	0.98
rs7708705	44922925	A	G	0.62	0.92	5.67E-16	0.90	7.88E-18	0.98	0.35	Imp	0.98
rs10039173	44903376	C	T	0.61	0.92	5.74E-16	0.91	1.03E-17	0.98	0.41	Gen	1.00
rs6870136	44910662	G	A	0.61	0.92	5.76E-16	0.91	1.41E-17	0.98	0.40	Gen	1.00
rs10060645	44908199	C	T	0.61	0.92	6.23E-16	0.91	1.57E-17	0.98	0.40	Imp	0.99
rs10045264	44886432	G	A	0.61	0.92	6.24E-16	0.91	1.93E-17	0.98	0.36	Gen	1.00
rs6880275	44908935	T	C	0.62	0.92	7.75E-16	0.91	1.48E-17	0.98	0.41	Imp	0.99
rs9791056	44903891	T	C	0.61	0.92	8.09E-16	0.91	1.25E-17	0.98	0.41	Gen	1.00
rs9791059	44900447	A	C	0.62	0.92	8.13E-16	0.90	8.88E-18	0.98	0.42	Imp	0.98
chr5:44904190:D	44904190	TAACTC	T	0.61	0.92	8.31E-16	0.91	1.55E-17	0.98	0.41	Imp	0.99
chr5:44842792:D	44842792	CT	C	0.56	0.92	8.78E-16	0.90	3.54E-19	1.00	0.97	Imp	0.94
chr5:44842794:D	44842794	CCAGTT	C	0.56	0.92	8.80E-16	0.90	3.56E-19	1.00	0.97	Imp	0.94
chr5:44842796:D	44842796	AGTTC	A	0.56	0.92	8.80E-16	0.90	3.55E-19	1.00	0.97	Imp	0.94
rs4457088	44900954	T	A	0.62	0.92	8.85E-16	0.91	1.29E-17	0.98	0.41	Imp	0.99
chr5:44782479:D	44782479	AT	A	0.38	1.08	8.87E-16	1.10	1.30E-17	1.02	0.37	Imp	0.98
rs4866921	44917137	T	C	0.62	0.92	9.83E-16	0.91	1.34E-17	0.98	0.41	Imp	0.99
rs9791164	44904030	A	G	0.62	0.92	9.92E-16	0.91	1.70E-17	0.98	0.42	Imp	0.99
rs6874167	44916674	G	A	0.62	0.92	1.01E-15	0.91	1.49E-17	0.98	0.41	Imp	0.99
rs4360054	44929004	A	G	0.61	0.92	1.10E-15	0.91	2.52E-17	0.98	0.41	Imp	0.98
rs6895062	44916492	T	C	0.62	0.92	1.12E-15	0.91	1.84E-17	0.98	0.40	Imp	0.98
chr5:44852861:D	44852861	AC	A	0.61	0.92	1.59E-15	0.91	6.38E-17	0.98	0.31	Imp	0.96
rs7449277	44841457	T	A	0.60	0.92	2.06E-15	0.91	7.34E-17	0.98	0.35	Imp	0.95
chr5:44885826:I	44885826	A	AAATT	0.61	0.92	2.29E-15	0.90	1.66E-17	0.99	0.53	Imp	0.96
rs7702464	44826259	A	C	0.27	1.11	2.32E-15	1.13	6.87E-17	1.03	0.20	Imp	0.74
chr5:44928978:D	44928978	ACCCTC	A	0.55	0.92	2.80E-15	0.90	3.15E-17	0.99	0.58	Imp	0.88
chr5:44921956:I	44921956	T	TA	0.61	0.92	3.19E-15	0.90	3.29E-17	0.99	0.54	Imp	0.95
rs74724331	44758161	G	A	0.40	1.08	3.28E-15	1.10	7.61E-17	1.01	0.47	Imp	0.95
rs13178923	44929285	T	A	0.57	0.92	1.03E-14	0.91	4.38E-15	0.98	0.34	Imp	0.87
rs12658334	44689131	G	A	0.39	1.09	2.72E-14	1.12	3.65E-16	1.02	0.50	Imp	0.73
chr5:44832897:I	44832897	A	ATGTT	0.59	0.93	5.26E-14	0.91	6.86E-16	0.99	0.79	Imp	0.94
rs183946926	44688992	G	A	0.37	1.09	1.05E-13	1.12	1.55E-16	1.03	0.25	Imp	0.69
chr5:44868315:D	44868315	AAT	A	0.48	0.93	1.26E-12	0.91	1.39E-13	0.97	0.13	Imp	0.79
rs200229088	44701817	TTG	T	0.31	1.09	2.28E-12	1.12	1.26E-13	1.04	0.15	Imp	0.65
rs4562047	44868093	C	T	0.48	0.93	1.52E-11	0.91	7.18E-14	0.99	0.68	Imp	0.77
rs2067980	44982317	A	G	0.16	1.09	2.51E-11	1.12	4.35E-13	1.04	0.17	Gen	1.00
rs73093976	44609392	C	T	0.17	1.09	6.67E-11	1.12	8.34E-13	1.02	0.36	Imp	0.95
rs147039293	44673324	C	T	0.14	0.92	3.98E-10	0.90	2.66E-10	0.93	0.01	Imp	0.96
rs75209549	44629545	A	G	0.14	1.09	6.64E-10	1.11	1.25E-10	1.02	0.53	Imp	0.89
rs112234443	44922557	C	T	0.05	1.16	1.10E-09	1.19	5.72E-10	1.07	0.14	Imp	0.90
rs76001691	44633373	T	C	0.05	1.16	1.14E-09	1.18	4.23E-09	1.09	0.07	Imp	0.82

rs4549535	44630291	A	G	0.05	1.15	1.58E-09	1.17	9.53E-10	1.08	0.09	Imp	0.93
rs6880469	44975989	A	G	0.35	1.06	1.83E-09	1.08	2.19E-10	1.01	0.57	Imp	0.98
rs6451787	45009269	T	G	0.17	1.09	2.53E-09	1.11	2.10E-10	1.03	0.23	Imp	0.84
rs28705196	44631795	T	C	0.05	1.14	4.04E-09	1.17	1.01E-09	1.06	0.18	Imp	0.97
chr5:44633359:I	44633359	T	TTC	0.05	1.14	4.39E-09	1.17	1.36E-09	1.06	0.17	Imp	0.95
chr5:44633372:I	44633372	T	TC	0.05	1.14	4.39E-09	1.17	1.36E-09	1.06	0.17	Imp	0.95
rs5004228	44632008	A	G	0.05	1.14	4.63E-09	1.16	2.03E-09	1.07	0.13	Gen	1.00
rs116443643	44606969	C	A	0.15	0.92	5.30E-09	0.91	1.78E-09	0.95	0.04	Imp	0.95
rs13357090	44632713	C	T	0.05	1.14	7.05E-09	1.16	3.72E-09	1.07	0.12	Imp	0.96
chr5:44681918:I	44681918	C	CA	0.04	0.84	7.16E-09	0.82	3.71E-08	0.88	0.03	Imp	0.58
c5_pos44839303	44803546	A	G	0.14	0.92	7.49E-09	0.91	2.36E-08	0.93	0.01	Gen	1.00
rs112754768	44718764	A	G	0.05	1.15	8.65E-09	1.18	6.28E-09	1.06	0.19	Imp	0.88
rs34692501	45029217	A	G	0.13	1.09	9.49E-09	1.11	7.72E-10	1.03	0.30	Gen	1.00
rs10941712	45917605	C	T	0.23	0.93	9.61E-09	0.91	1.42E-10	0.95	0.02	Imp	0.78
rs11750654	44494303	T	C	0.15	0.92	1.13E-08	0.91	2.84E-09	0.95	0.04	Imp	0.95
rs11750655	44494312	T	C	0.15	0.92	1.13E-08	0.91	2.84E-09	0.95	0.04	Imp	0.95
rs3935086	44960923	T	A	0.33	1.06	1.14E-08	1.07	2.76E-09	1.01	0.64	Gen	1.00
rs79670114	44861045	A	G	0.04	1.16	1.24E-08	1.19	2.52E-09	1.06	0.23	Imp	0.88
rs11742346	45003293	C	T	0.13	1.09	1.29E-08	1.11	1.45E-09	1.03	0.24	Imp	0.94
rs12520604	44476872	A	C	0.04	1.16	1.34E-08	1.20	4.72E-09	1.03	0.56	Imp	0.91
c5_pos44832736	44796979	C	T	0.14	0.92	1.36E-08	0.91	4.34E-08	0.93	0.01	Gen	0.99
c5_pos44671855	44636098	G	C	0.20	0.93	1.41E-08	0.92	6.26E-09	0.96	0.10	Gen	1.00
rs187108781	44619502	A	G	0.15	0.92	1.59E-08	0.91	6.61E-09	0.95	0.04	Imp	0.95
chr5:44795636:I	44795636	A	AG	0.28	1.07	1.68E-08	1.09	9.96E-10	1.00	0.87	Imp	0.85
c5_pos45369617	45333860	T	C	0.26	0.94	1.71E-08	0.92	1.56E-10	0.96	0.05	Gen	1.00
rs12516986	44526154	A	G	0.04	1.15	1.80E-08	1.18	6.09E-09	1.03	0.50	Gen	1.00
rs7702731	44914285	A	G	0.68	0.94	2.80E-08	0.94	2.63E-08	0.99	0.71	Gen	0.98
rs75036127	44802928	A	T	0.04	1.15	3.47E-08	1.19	5.58E-09	1.05	0.33	Imp	0.88
rs72748037	44449376	G	A	0.15	0.93	3.51E-08	0.91	1.02E-08	0.95	0.04	Imp	0.97
rs13183434	45074633	G	A	0.13	1.08	3.67E-08	1.11	1.91E-09	1.04	0.21	Gen	1.00
chr5:45943429:D	45943429	CTCT	C	0.16	0.92	3.72E-08	0.89	1.09E-09	0.92	0.01	Imp	0.70
rs11745472	44929256	A	T	0.12	1.11	3.83E-08	1.13	5.18E-08	1.03	0.39	Imp	0.61
rs72748026	44431924	C	G	0.14	0.92	3.86E-08	0.91	3.78E-09	0.95	0.05	Imp	0.95
rs17343002	44853593	G	C	0.31	0.94	4.36E-08	0.94	9.13E-08	0.96	0.06	Gen	1.00
c5_pos44489027	44453270	T	G	0.15	0.93	4.89E-08	0.91	1.65E-08	0.95	0.04	Gen	1.00
rs11738503	45011742	A	G	0.16	1.08	5.09E-08	1.10	4.71E-09	1.04	0.19	Imp	0.87
rs11741260	44412065	G	A	0.14	0.93	5.14E-08	0.91	5.32E-09	0.95	0.05	Gen	1.00
rs11948186	45051434	A	G	0.34	1.06	5.17E-08	1.07	7.10E-09	1.01	0.69	Imp	0.97
rs7701656	44947702	T	C	0.13	1.08	5.71E-08	1.11	2.64E-09	1.00	0.90	Gen	1.00
chr5:44692568:I	44692568	G	GT	0.21	0.93	6.86E-08	0.92	8.13E-08	0.96	0.09	Imp	0.90
rs78797445	44795797	G	A	0.19	0.94	8.81E-08	0.93	1.30E-07	0.96	0.08	Imp	0.97

chr5:45162687:D	45162687	AGATCT	A	0.14	0.93	1.03E-07	0.91	2.86E-08	0.93	0.01	Imp	0.95
c5_pos44754116	44718359	G	A	0.19	0.94	1.03E-07	0.93	1.25E-07	0.95	0.05	Gen	1.00
rs111385188	44675187	G	A	0.04	1.16	1.22E-07	1.19	2.96E-08	1.07	0.22	Imp	0.85
rs62372990	45988038	G	A	0.06	1.14	1.26E-07	1.15	3.53E-07	1.07	0.12	Imp	0.76
c5_pos44754266	44718509	G	A	0.07	1.11	1.27E-07	1.15	5.01E-10	1.04	0.30	Gen	1.00
rs7293402	45990381	G	A	0.06	1.13	1.40E-07	1.15	4.28E-07	1.07	0.13	Imp	0.76
rs147872430	45239336	C	T	0.14	0.93	1.52E-07	0.91	3.35E-08	0.93	0.01	Imp	0.96
chr5:44945030:I	44945030	T	TA	0.19	0.93	1.76E-07	0.93	3.34E-07	0.96	0.08	Imp	0.92
rs10051592	45090306	T	G	0.33	1.06	1.83E-07	1.07	1.67E-08	1.01	0.79	Gen	1.00
c5_pos45171325	45135568	G	C	0.14	0.93	1.97E-07	0.91	4.97E-08	0.93	0.01	Gen	1.00
chr5:44518580:I	44518580	C	CT	0.21	0.94	2.25E-07	0.93	2.21E-06	0.93	0.00	Imp	0.87
rs11743309	45122388	A	G	0.14	0.93	2.27E-07	0.92	8.71E-08	0.93	0.00	Gen	1.00
c5_pos45322045	45286288	T	A	0.14	0.93	2.61E-07	0.92	6.68E-08	0.94	0.01	Gen	0.99
c5_pos45028737	44992980	G	T	0.14	0.93	3.09E-07	0.92	1.57E-06	0.94	0.02	Gen	1.00
c5_pos45149948	45114191	C	T	0.14	0.93	3.74E-07	0.92	1.15E-07	0.93	0.01	Gen	1.00
rs9763350	45882593	T	C	0.26	1.07	4.25E-07	1.08	6.84E-07	1.08	0.00	Imp	0.71
rs11740651	45302476	C	A	0.14	0.93	7.20E-07	0.91	7.54E-08	0.94	0.03	Imp	0.91
chr5:44976998:I	44976998	G	GTGAT	0.11	1.08	9.83E-07	1.11	5.80E-08	1.02	0.44	Imp	0.93
rs13172124	45160377	C	T	0.46	0.95	1.13E-06	0.94	4.25E-07	0.98	0.22	Imp	0.96
rs13186320	44302177	A	T	0.47	1.05	1.19E-06	1.07	1.46E-07	1.04	0.05	Imp	0.86
rs72750030	45903837	G	A	0.11	0.92	1.26E-06	0.89	1.33E-07	0.92	0.02	Imp	0.70
rs72751936	45984433	G	A	0.11	0.91	1.33E-06	0.88	2.66E-07	0.90	0.01	Imp	0.59
c5_pos45135048	45099291	G	A	0.09	0.92	1.53E-06	0.91	7.00E-06	0.92	0.01	Gen	1.00
rs147517548	44711323	C	T	0.01	1.3	1.55E-06	1.37	5.35E-07	1.18	0.11	Imp	0.68
rs13156720	44823321	A	G	0.02	1.19	1.79E-06	1.25	1.18E-07	1.08	0.28	Imp	0.77
c5_pos44444935	44409178	C	A	0.06	0.91	1.90E-06	0.89	8.08E-07	0.95	0.18	Gen	1.00
rs4866929	45266589	A	G	0.47	0.95	1.98E-06	0.94	5.79E-07	0.98	0.21	Gen	1.00
chr5:45210850:D	45210850	CTATAA	C	0.45	0.95	2.02E-06	0.94	7.09E-07	0.97	0.17	Imp	0.92
rs11959880	45958226	G	A	0.09	0.9	2.06E-06	0.89	2.01E-06	0.90	0.01	Imp	0.58
rs17268417	44700201	T	C	0.05	1.12	2.08E-06	1.15	4.60E-07	1.08	0.07	Gen	0.95
chr5:45210851:D	45210851	TATAAA	T	0.45	0.95	2.31E-06	0.94	7.39E-07	0.97	0.16	Imp	0.92
chr5:44349569:D	44349569	TATCAGA	T	0.43	1.06	2.33E-06	1.06	3.88E-06	1.03	0.22	Imp	0.70
rs11738948	44999799	G	C	0.20	0.94	2.45E-06	0.94	1.32E-05	0.95	0.05	Imp	0.96
rs72750027	45899603	G	A	0.01	1.43	2.55E-06	1.46	1.45E-05	1.42	0.01	Imp	0.66
rs72765759	45846620	C	T	0.11	0.92	2.91E-06	0.90	2.28E-07	0.94	0.06	Imp	0.73
rs981782	45285718	A	C	0.47	0.96	3.13E-06	0.95	8.27E-07	0.98	0.30	Imp	0.97
rs137877813	45084544	G	C	0.14	0.94	3.13E-06	0.93	4.96E-06	0.94	0.02	Imp	0.96
rs138117035	45938300	T	C	0.10	0.91	3.23E-06	0.89	7.08E-07	0.93	0.05	Imp	0.61
rs145696903	45933347	G	A	0.10	0.91	3.38E-06	0.89	9.01E-07	0.92	0.04	Imp	0.61
chr5:44302175:D	44302175	CA	C	0.51	1.05	3.46E-06	1.06	3.54E-07	1.03	0.17	Imp	0.94
rs75442098	44498735	C	A	0.03	0.86	3.56E-06	0.84	1.40E-05	0.91	0.15	Imp	0.65

rs16902086	45285752	A	G	0.34	1.05	3.60E-06	1.06	6.45E-07	1.02	0.34	Gen	1.00
rs72762052	45427937	C	T	0.13	0.93	3.83E-06	0.91	6.81E-07	0.93	0.03	Imp	0.81
rs72750038	45912279	T	C	0.10	0.91	3.87E-06	0.89	1.15E-06	0.92	0.02	Imp	0.65
rs74833952	44961224	G	A	0.02	0.76	4.76E-06	0.75	2.64E-05	0.80	0.06	Imp	0.42
rs11747840	44984579	C	T	0.21	0.95	4.97E-06	0.94	1.74E-05	0.96	0.08	Imp	0.94
rs72751917	45949866	G	A	0.01	1.4	5.21E-06	1.40	6.02E-05	1.38	0.02	Imp	0.51
rs11750845	44373060	C	T	0.51	1.05	5.39E-06	1.06	4.54E-07	1.02	0.27	Gen	1.00
rs72759922	45005236	C	T	0.21	0.95	6.67E-06	0.94	1.09E-05	0.97	0.16	Imp	0.94
rs72763959	45707287	C	A	0.01	1.33	6.84E-06	1.36	3.85E-05	1.28	0.03	Imp	0.69
rs35190075	44776604	C	G	0.02	1.18	7.49E-06	1.25	5.20E-07	1.11	0.15	Imp	0.76
rs185330077	45366544	G	A	0.01	1.48	7.60E-06	1.69	9.92E-08	0.94	0.70	Imp	0.39
rs182455548	45746470	G	A	0.01	1.33	7.67E-06	1.35	3.70E-05	1.28	0.04	Imp	0.69
rs72763975	45767075	G	A	0.01	1.33	7.78E-06	1.35	3.66E-05	1.28	0.04	Imp	0.69
c5_pos44897529	44861772	T	C	0.04	1.11	8.41E-06	1.14	9.56E-07	1.05	0.26	Gen	1.00
rs72763992	45806419	A	T	0.01	1.33	8.60E-06	1.36	3.32E-05	1.28	0.04	Imp	0.67
rs148456234	45922475	C	T	0.11	0.92	8.93E-06	0.90	1.29E-06	0.93	0.05	Imp	0.65
rs72750006	45862423	A	G	0.01	1.3	8.95E-06	1.35	1.57E-05	1.24	0.05	Imp	0.63
rs72762048	45408899	T	C	0.12	0.93	9.74E-06	0.91	2.49E-06	0.93	0.03	Imp	0.80
rs5003385	44907327	A	C	0.04	1.11	1.01E-05	1.14	9.29E-07	1.05	0.27	Gen	1.00
rs191491730	45160378	G	A	0.00	1.6	1.03E-05	1.83	4.17E-07	1.21	0.37	Imp	0.59
rs148415520	45317307	G	A	0.45	0.96	1.13E-05	0.95	6.59E-06	0.97	0.12	Imp	0.91
rs76810418	45882608	G	A	0.25	1.06	1.21E-05	1.07	1.71E-05	1.06	0.02	Imp	0.74
chr5:44606887:D	44606887	AT	A	0.15	0.94	1.28E-05	0.93	2.00E-05	0.97	0.26	Imp	0.95
rs147763247	45313905	G	C	0.45	0.96	1.32E-05	0.95	6.73E-06	0.97	0.12	Imp	0.91
rs10473395	45882607	T	C	0.25	1.06	1.52E-05	1.07	2.30E-05	1.06	0.02	Imp	0.74
chr5:45799799:D	45799799	AT	A	0.04	1.12	1.59E-05	1.13	5.54E-05	1.06	0.26	Imp	0.81
rs192741232	44838737	A	G	0.01	1.3	1.59E-05	1.38	4.74E-06	1.14	0.27	Imp	0.75
rs150276216	44889423	T	C	0.04	1.11	1.71E-05	1.14	2.26E-06	1.06	0.26	Imp	0.97
rs191712805	45795107	A	G	0.01	1.33	1.72E-05	1.36	5.89E-05	1.28	0.05	Imp	0.61
rs4613718	44649944	C	T	0.60	1.04	1.92E-05	1.06	5.73E-07	0.98	0.22	Gen	1.00
rs114416420	45951524	G	T	0.10	0.91	2.00E-05	0.90	3.06E-05	0.90	0.01	Imp	0.56
c5_pos44663080	44627323	C	T	0.21	0.95	2.02E-05	0.95	4.76E-05	0.95	0.04	Gen	1.00
rs180724159	45562846	T	C	0.00	1.53	2.04E-05	1.71	1.34E-06	1.20	0.36	Imp	0.72
rs181673846	45837084	C	A	0.01	1.34	2.08E-05	1.38	4.81E-05	1.29	0.05	Imp	0.69
chr5:45853664:D	45853664	CAT	C	0.02	1.22	2.15E-05	1.22	1.25E-04	1.10	0.30	Imp	0.58
rs139424826	45522979	C	A	0.12	0.93	2.44E-05	0.90	1.23E-06	0.94	0.10	Imp	0.68
rs6859397	45136050	T	C	0.33	1.04	2.79E-05	1.06	4.14E-06	1.00	0.83	Imp	0.95
rs4492120	45187804	G	A	0.33	1.04	2.82E-05	1.06	2.90E-06	1.00	0.81	Gen	1.00
chr5:44349567:D	44349567	TATAT	T	0.38	1.05	2.86E-05	1.06	2.14E-05	1.02	0.43	Imp	0.69
rs10035564	45252500	A	G	0.34	1.04	2.98E-05	1.06	2.91E-06	1.01	0.72	Gen	0.96
rs75513092	44544909	G	T	0.21	0.95	3.22E-05	0.95	6.48E-05	0.96	0.06	Imp	0.98

rs6867827	45317216	G	T	0.17	1.06	3.36E-05	1.06	5.26E-05	1.04	0.11	Imp	0.97
rs139928702	45938421	A	C	0.05	1.12	3.47E-05	1.13	1.02E-04	1.08	0.18	Imp	0.62
rs11748830	44265513	C	T	0.16	0.94	3.70E-05	0.94	3.87E-05	0.94	0.02	Imp	0.92
rs17268006	44538881	C	T	0.21	0.95	4.19E-05	0.95	9.20E-05	0.96	0.05	Gen	0.99
chr5:45686224:D	45686224	TA	T	0.07	1.12	4.22E-05	1.09	5.64E-03	1.15	0.01	Imp	0.50
chr5:44816722:D	44816722	ACTT	A	0.04	1.11	4.29E-05	1.14	7.24E-06	1.07	0.18	Imp	0.95
rs72759920	44998794	T	G	0.04	1.11	5.13E-05	1.13	1.88E-05	1.06	0.21	Imp	0.92
rs72759916	44990010	T	C	0.04	1.11	6.09E-05	1.13	1.69E-05	1.06	0.24	Imp	0.92
rs62370579	45792329	A	T	0.03	1.15	6.40E-05	1.15	7.86E-04	1.10	0.17	Imp	0.77
chr5:45843775:D	45843775	TTA	T	0.04	1.12	6.78E-05	1.13	2.15E-04	1.06	0.29	Imp	0.84
rs181701912	44888831	C	T	0.11	0.92	6.92E-05	0.90	2.49E-05	0.96	0.36	Imp	0.51
rs10078961	44888856	T	C	0.29	0.95	7.00E-05	0.93	8.16E-06	0.99	0.73	Imp	0.63
chr5:45379590:D	45379590	CA	C	0.19	1.05	7.02E-05	1.06	7.32E-05	1.04	0.15	Imp	0.97
rs75946047	44454153	C	T	0.03	1.14	7.34E-05	1.20	6.92E-06	1.08	0.26	Imp	0.81
rs62372989	45987296	A	G	0.03	1.14	7.45E-05	1.15	3.22E-04	1.07	0.31	Imp	0.70
rs12187122	44577879	A	G	0.03	1.13	7.77E-05	1.16	3.14E-05	1.01	0.93	Imp	0.91
rs6451798	45387854	C	T	0.19	1.05	7.93E-05	1.06	8.43E-05	1.03	0.16	Gen	1.00
rs62370581	45793718	G	A	0.03	1.12	8.32E-05	1.12	3.67E-04	1.06	0.28	Imp	0.91
rs1816683	44701827	G	A	0.57	1.04	8.34E-05	1.06	2.73E-06	0.98	0.30	Imp	0.85
rs75964308	45946479	G	C	0.04	1.12	8.48E-05	1.13	2.36E-04	1.06	0.33	Imp	0.77
rs1501362	45378207	C	T	0.19	1.05	8.52E-05	1.06	7.26E-05	1.03	0.18	Imp	0.98
rs141075872	45790426	A	T	0.03	1.13	8.55E-05	1.13	7.34E-04	1.07	0.25	Imp	0.82
rs7709262	45342044	A	G	0.19	1.05	8.69E-05	1.06	8.62E-05	1.03	0.18	Imp	0.98
rs144163740	45376455	G	T	0.36	0.95	8.87E-05	0.94	3.12E-05	0.98	0.52	Imp	0.61
rs62370604	45843007	T	C	0.03	1.12	9.55E-05	1.13	3.17E-04	1.06	0.32	Imp	0.86
rs74859464	45951751	C	A	0.03	1.12	9.58E-05	1.13	2.33E-04	1.06	0.33	Imp	0.83
rs6874127	45305615	A	G	0.19	1.05	9.63E-05	1.06	9.12E-05	1.03	0.17	Gen	1.00
rs75212852	45805592	C	T	0.03	1.12	9.76E-05	1.12	4.25E-04	1.06	0.29	Imp	0.91
chr5:45804917:I	45804917	T	TA	0.03	1.12	9.79E-05	1.12	4.27E-04	1.06	0.29	Imp	0.91
c5_pos45103929	45068172	G	A	0.04	1.1	9.85E-05	1.12	1.27E-04	1.05	0.31	Gen	1.00

Supplementary Table 2. The three independent signals showing the lead SNP in each signal together with the correlated SNPs that could not be excluded from causality.

SNP	Position	Ref/Alt	MAF	Imput-ation r2	OR (95% CI)	Single SNP p_value	P-value conditional on signal 1 top hit	P-value conditional on signal 1 + 2	cor_ rs10941679	cor_rs6864776	cor_ rs200229088
**Signal 1**											
rs10941679	44706498	A/G	0.27	0.99	1.12(1.1-1.15)	2.60E-26	NA	NA	1.00	0.07	0.14
**Signal 2**											
rs6864776	44513304	G/A	0.23	0.97	1.04(1.02-1.06)	0.00078	6.22E-11	NA	0.07	1.00	0.05
chr5.44527739.I	44527739	ATACT/A	0.24	0.96	1.04(1.02-1.07)	0.00044	6.40E-11	NA	0.06	0.98	0.05
rs4634356	44553611	C/T	0.23	0.99	1.04(1.02-1.06)	0.00076	7.89E-11	NA	0.07	0.99	0.06
rs1905192	44531538	A/T	0.23	0.99	1.04(1.02-1.06)	0.00077	8.76E-11	NA	0.07	0.99	0.06
rs4866905	44555867	C/T	0.23	1.00	1.04(1.02-1.06)	0.00086	1.02E-10	NA	0.07	0.98	0.06
rs1482663	44543102	A/G	0.23	1.00	1.04(1.02-1.06)	0.00085	1.04E-10	NA	0.07	0.99	0.06
chr5.44496660.I	44496660	AG/A	0.23	0.98	1.04(1.02-1.06)	0.00087	1.04E-10	NA	0.06	0.99	0.05
rs7710996	44514350	G/A	0.23	0.99	1.04(1.02-1.06)	0.00085	1.07E-10	NA	0.06	0.99	0.05
rs6451763	44527841	C/A	0.23	0.99	1.04(1.02-1.06)	0.00087	1.09E-10	NA	0.07	0.99	0.05
c5_pos44562807	44527050	A/C	0.23	1.00	1.04(1.02-1.06)	0.00087	1.15E-10	NA	0.07	0.99	0.06
rs1351633	44543851	C/T	0.23	0.99	1.04(1.02-1.06)	0.00094	1.18E-10	NA	0.07	0.99	0.06
rs1384453	44496114	T/C	0.23	0.99	1.04(1.02-1.06)	0.00091	1.20E-10	NA	0.06	0.99	0.05
rs1482665	44546628	G/A	0.23	1.00	1.04(1.02-1.06)	0.00093	1.20E-10	NA	0.07	0.99	0.06
rs983940	44544136	G/A	0.23	0.99	1.04(1.02-1.06)	0.00095	1.21E-10	NA	0.07	0.99	0.06
rs6897963	44554781	C/A	0.23	1.00	1.04(1.02-1.06)	0.00098	1.33E-10	NA	0.07	0.98	0.06
rs1384454	44492998	C/T	0.23	0.99	1.04(1.02-1.06)	0.00098	1.43E-10	NA	0.06	0.99	0.05
rs10079222	44561473	G/C	0.23	1.00	1.04(1.02-1.06)	0.001	1.45E-10	NA	0.07	0.98	0.06
rs7736427	44524276	A/G	0.25	0.93	1.04(1.02-1.07)	0.00028	1.48E-10	NA	0.05	0.94	0.05
rs10512860	44539953	G/C	0.23	1.00	1.04(1.02-1.06)	0.001	1.50E-10	NA	0.07	0.99	0.05
rs4866776	44525589	T/A	0.23	1.00	1.04(1.02-1.06)	0.001	1.52E-10	NA	0.07	0.99	0.05
rs1482690	44488840	T/A	0.24	0.98	1.04(1.02-1.06)	0.0011	1.91E-10	NA	0.06	0.99	0.05
rs12516346	44488156	A/C	0.23	0.99	1.04(1.01-1.06)	0.0016	2.39E-10	NA	0.07	0.97	0.06
rs1482684	44478742	G/A	0.23	1.00	1.04(1.01-1.06)	0.0013	2.41E-10	NA	0.06	0.99	0.05
chr5.44496659.I	44496659	TA/T	0.25	0.93	1.04(1.01-1.06)	0.0017	2.41E-10	NA	0.07	0.95	0.05
rs1482691	44487477	C/A	0.23	0.99	1.04(1.01-1.06)	0.0013	2.52E-10	NA	0.06	0.99	0.05
rs7724859	44471395	T/C	0.23	1.00	1.04(1.01-1.06)	0.0014	2.84E-10	NA	0.06	0.99	0.05
rs2128430	44462926	T/C	0.23	0.99	1.04(1.01-1.06)	0.0014	3.03E-10	NA	0.06	0.99	0.05
rs7707044	44471392	A/T	0.23	1.00	1.04(1.01-1.06)	0.0015	3.15E-10	NA	0.06	0.99	0.05
rs1905191	44456782	C/T	0.23	0.98	1.04(1.01-1.06)	0.0015	3.21E-10	NA	0.06	0.99	0.05

rs1120718	44466578	T/C	0.23	1.00	1.04(1.01-1.06)	0.0017	3.86E-10	NA	0.06	0.99	0.05
rs4866899	44444857	C/T	0.23	0.98	1.04(1.01-1.06)	0.0017	4.49E-10	NA	0.06	0.98	0.05
rs7712213	44451269	A/C	0.23	0.98	1.04(1.01-1.06)	0.002	5.30E-10	NA	0.07	0.98	0.05
rs6451762	44463186	C/T	0.24	0.96	1.04(1.01-1.06)	0.0023	7.36E-10	NA	0.06	0.98	0.05
rs7703171	44441175	C/T	0.23	0.95	1.04(1.01-1.06)	0.0018	8.24E-10	NA	0.06	0.95	0.05
rs6879342	44473605	A/G	0.24	0.98	1.03(1.01-1.06)	0.0067	3.57E-09	NA	0.07	0.96	0.06
**Signal 3**											
rs200229088	44701817	TTG/T	0.31	0.65	1.09(1.07-1.12)	2.30E-12	5.61E-04	1.12E-05	0.14	0.05	1.00

Supplementary Table 3. ER/PR status, genotype and copy number at 5p12 of breast cell lines used in functional assays.

	ER[a] status	PR[b] status	Signal 1 rs10941679	Signal 2 rs6864776	Signal 3 rs200229088	Copy Number[c]	Haplotype
SVCT	−	−	AA	GG	TTG/T	Not det	A/B
MCF10A	−	−	AA	GG	TTG/T	Not det	A/B
T47D	+	+	AA	AA	TTG/T	Gain	C/D
ZR751	+	+	AA	AA	TTG/T	Normal	C/D
MDA-MB-361	+	−	AA	AA	TTG/T	Gain	C/D
MCF7	+	+	AA	AG	TTG/T	Gain	Not det
Hs578T	−	−	AA	AG	TTG/T	Gain	Not det
BT474	+	+	AG	AG	TTG/T	Gain	Not det
Bre80	−	−	AG	AG	TTG/T	Not det	Not det
MDA-MB-231	−	−	AG	AG	TTG/T	Gain	Not det
BT-20	−	−	GG	AA	TTG/T	Gain	G/H
MDA-MB-468	−	−	GG	AA	TTG/T	Gain	G/H

[a]Estrogen receptor-alpha, [b]Progesterone receptor, [c]Copy number status from array CGH data (segmentation of normalized log2 ratios) generated by the Cancer Cell Line Encyclopedia (CCLE), Not det = Not determined.

Supplementary Table 4. Oligonucleotides used for qRTPCR of putative noncoding RNAs.

qRT Primer	Sequence (5' to 3')
FGF10-AS1FOR	TGCACCAACATCCATAACTCCTCG
FGF10-AS1REV	CTTTGTGAAGGAAAGCTGGACAGG
BRCAT54FOR	TGTGTGCTTAGACCTGACGCTGG
BRCAT54REV	CTCCTCACATGGCAGGAAGAAGG
RP11-503D12.1FOR	CAAGGTCAGTTGGCATTGTTCTGG
RP11-503D12.1REV	CAACCACCATGTCAAGGAACTCTAGC
RP11-473L15.3FOR	CACATGCATTGGCTATGACCTTGC
RP11-473L15.3REV	GCTGGCCATAAAGCTTCAATGTCC
TATA-binding proteinFOR	CATGGATCAGAACAACAGCCTGC
TATA-binding proteinREV	TTGTGAGAGTCTGTGAGTGGAAGAGC

Supplementary Table 5. Cell lines used in the study.

Cell line[1,2]	Source
SVCT	ECACC 94122105
MCF10A	ATCC CRL-10317
T47D	ATCC HTB-133
ZR751	ATCC CRL-1500
MDA-MB-361	Gift from S. Lankani, UQCCR, AUS
MCF7	ATCC HTB-22
Hs578T	ATCC HTB-126
BT474	ATCC HTB-20
Bre80	Gift from R. Reddel, CMRI, AUS
MDA-MB-231	ATCC HTB-26
BT-20	ATCC HTB-19
MDA-MB-468	ATCC HTB-132

[1] Cell lines used in experiments were within passages 15-30.
[2] Cell lines were routinely tested for mycoplasma and short tandem repeat (STR) profiled.

Supplementary Table 6. Oligonucleotides used in 3C assays.

**3C Primer (*Eco*RI)**	**Sequence (5' to 3')**
FGF10 promoter bait	GCTGCTCTTCTGTACAGCGTGATGACAAGAGG
MRPS30 promoter bait	CCTTCACAGAAGTAAGCAACATGAGGGAAGC
HCN1 promoter bait	TCCCACTGGCACCTGCAAGTATGTGC
PRE Fragment 1	CTAAGACTGTCTCACCATGGAGCAACTCATGC
PRE Fragment 2	TGGCTTCCTGCCACTTGTTTCCTAATGC
PRE Fragment 3	CTCCTTGCTGTGAGAAGGTTGCACATCC
PRE Fragment 4	AGCACAAGAGCAGCCTCTACTGGGATATGC
PRE Fragment 5	TCCAAGTGAGTCAGTCTCCTGCCTCAGG
PRE Fragment 6	TCACTTGCTTTCTAGATTTGTGATTCTGCTTTCC
PRE Fragment 7	GTCCAGAGTCAAGTGGAGGAGATAACTCAAGGG
PRE Fragment 8	GAGGAATCAGGCAATCTTCATAAATATGGCTTACC
PRE Fragment 9	GGAATGCAAAGTGCAGCTATTGCTCTGC
PRE Fragment 10	GAAAATTTGGCATCATCCTTAGTGCACAGTAGG
PRE Fragment 11	CTGTGTAAGTTACAGAGCCATGATCATTGGTGG
PRE Fragment 12	GCCACATCCCTAGATGCCATGGTCC

Supplementary Table 7. Oligonucleotides used in cloning luciferase constructs.

Primer	Sequence (5' to 3')
FGF10 promoter FOR	GATCGCCATAAAGTGCGTTTGC
FGF10 promoter REV	CTGGAAGGGTAAGACCCGATGC
FGF10-AS1 promoter FOR	CCCAGGCATAATTTACGCTGAGG
FGF10-AS1 promoter REV	GCATTCACTTCTGGCCAGATCC
MRPS30 promoter FOR	ACTTTCCATGGCTCTGACTCAGTCC
MRPS30 promoter REV	CGGACCCAGAGGTCAACTTAAGC
BRCAT54 promoter FOR	CCTATCGTGAACGGTTATTGTGAGC
BRCAT54 promoter REV	AGTTCCTGGCAAAGGAGGTTGC
5p12 enhancer FOR	GACTCTGTATCTTCACGCATTTCCAGG
5p12 enhancer REV	TTAGAGACCAGAAATGTGGGCAAAGG
rs10941679 mut FOR	GCTTTTTATTGACTGTGGAAAGAACACAGC
rs10941679 mut REV	GCTGTGTTCTTTCCACAGTCAATAAAAAGC

Supplementary Table 8. Oligonucleotides used in EMSAs.

SNP	allele[a]	Sequence (5' to 3')[b]
rs10941679	com	[BIO]GCTTTTTATTGACTATGGAAAGAACACAGC
rs10941679	min	[BIO]GCTTTTTATTGACTGTGGAAAGAACACAGC
Neg control		AGAGATTGCCTGACGTCAGAGAGCTAG

[a] com: common allele, min: minor allele

[b] BIO: 5' biotinylation (present on both the sense and antisense strands of the duplex)

Supplementary Table 9. EMSA competitor duplexes and their target DNA binding proteins.

Competition Target	Sequence (5' to 3')
AP1	CGCTTGATGACTCAGCCGGAA
AP2	GATCGAACTGACCGCCCGCGGCCCGT
CEBP	TGCAGATTGCGCAATCTGCA
CTCF	AAGAAACCGCTAGGGGGCCTACT
FOXA1	CTGGTCTTAAAGGTGTTTACCTTGTCTGAT
FOXA2	GTTGACTAAGTCAATAATCAGAATCAG
GATA3	CACTTGATAACAGAAAGTGATAACTCT
HNF6	GATTCCATATTGATTTCAAAA
HOXA10	GCATTCAGAAGGTTATAGCTTT
MYC	TCAGACCACGTGGTCGGG
NFkB	AGTTGAGGGGACTTTCCCAGGC
OCT1	TGTCGAATGCAAATCACTAGAA
SP1	ATTCGATCGGGGCGGGGCGAGC

Supplementary Table 10. Oligonucleotides used for ChIP-qPCR analyses.

Name	Sequence (5' to 3')
ChIP rs10941679 FOR	TTGAATCAGATGTGTAGTGCTTCC
ChIP rs10941679 REV	AACCTGAATGCAGCAAAATAGC
ChIP negcontrol FOR	CTAAGGACGAGATGCACATGG
ChIP negcontrol REV	TCAAGTTTCCAACTCCAACAGG

CONSORTIA

For members of kConFab, see http://www.kconfab.org/Organisation/Members.aspx; for AOCS, see http://www.aocstudy.org/org_coll.asp.

Norwegian Breast Cancer Study (NBCS) members:

Kristine K.Sahlberg, Lars Ottestad, Rolf Kåresen, Anita Langerød, Ellen Schlichting, Marit Muri Holmen, Toril Sauer, Vilde Haakensen, Olav Engebråten, Bjørn Naume, Cecile E. Kiserud, Kristin V. Reinertsen, Åslaug Helland, Margit Riis, Ida Bukholm, Per Eystein Lønning, Jürgen Geisler, Grethe I. Grenaker Alnæs, OSBREAC

BCAC INVESTIGATING GROUPS:

The Australian Breast Cancer Family Study (ABCFS) would like to thank Maggie Angelakos, Judi Maskiell, Gillian Dite. ABCFS was supported by grant UM1 CA164920 from the National Cancer Institute (USA). The content of this manuscript does not necessarily reflect the views or policies of the National Cancer Institute or any of the collaborating centers in the Breast Cancer Family Registry (BCFR), nor does mention of trade names, commercial products, or organizations imply endorsement by the USA Government or the BCFR. The ABCFS was also supported by the National Health and Medical Research Council of Australia, the New South Wales Cancer Council, the Victorian Health Promotion Foundation (Australia) and the Victorian Breast Cancer Research Consortium. J.L.H. is a National Health and Medical Research Council (NHMRC) Australia Fellow and a Victorian Breast Cancer Research Consortium Group Leader. M.C.S. is a NHMRC Senior Research Fellow and a Victorian Breast Cancer Research Consortium Group Leader. The ABCS study was supported by the Dutch Cancer Society [grants NKI 2007-3839; 2009 4363]; BBMRI-NL, which is a Research Infrastructure financed by the Dutch government (NWO 184.021.007); and the Dutch National Genomics Initiative. The ACP study wishes to thank the participants in the Thai Breast Cancer study. Special Thanks also go to the Thai Ministry of Public Health (MOPH), doctors and nurses who helped with the data collection process. Finally, the study would like to thank Dr Prat Boonyawongviroj, the former Permanent Secretary of MOPH and Dr Pornthep Siriwanarungsan, the Department Director-General of Disease Control who have supported the study throughout. The ACP study is funded by the Breast Cancer Research Trust, UK. The BBCC study would like to thank Matthias Rübner, Alexander Hein, Michael Schneider. The work of the BBCC was partly funded by ELAN-Fond of the University Hospital of Erlangen. The BBCS would like to thank Eileen Williams, Elaine Ryder-Mills, Kara Sargus. The BBCS is funded by Cancer Research UK and Breast Cancer Now and acknowledges NHS funding to the NIHR Biomedical Research Centre, and the National Cancer Research Network (NCRN). The BIGGS would like to thank Niall McInerney, Gabrielle Colleran, Andrew Rowan, Angela Jones. ES is supported by NIHR Comprehensive Biomedical Research Centre, Guy's & St. Thomas' NHS Foundation Trust in partnership with King's College London, United Kingdom. I.T. is supported by the Oxford Biomedical Research Centre and by core funding to the Wellcome Trust Centre for Human Genetics from the Wellcome Trust (090532/Z/09/Z). The BSUCH would like to thank Peter Bugert, Medical Faculty Mannheim. The BSUCH study was supported by the

Dietmar-Hopp Foundation, the Helmholtz Society and the German Cancer Research Center (DKFZ). The CECILE study was funded by Fondation de France, Institut National du Cancer (INCa), Ligue Nationale contre le Cancer, Ligue contre le Cancer Grand Ouest, Agence Nationale de Sécurité Sanitaire (ANSES), Agence Nationale de la Recherche (ANR). The CGPS study would like to thank Staff and participants of the Copenhagen General Population Study. It would also like to thank Dorthe Uldall Andersen, Maria Birna Arnadottir, Anne Bank and Dorthe Kjeldgård Hansen for the excellent technical assistance: The Danish Breast Cancer Group (DBCG) is acknowledged for the tumor information. The Danish Cancer Biobank is acknowledged for providing infrastructure for the collection of blood samples for the cases. The CGPS was supported by the Chief Physician Johan Boserup and Lise Boserup Fund, the Danish Medical Research Council and Herlev Hospital. The CNIO-BCS would like to thank Guillermo Pita, Charo Alonso, Daniel Herrero, Nuria Álvarez, Pilar Zamora, Primitiva Menendez, the Human Genotyping-CEGEN Unit (CNIO). The CNIO-BCS was supported by the Genome Spain Foundation, the Red Temática de Investigación Cooperativa en Cáncer and grants from the Asociación Española Contra el Cáncer and the Fondo de Investigación Sanitario (PI11/00923 and PI081120). The Human Genotyping-CEGEN Unit (CNIO) is supported by the Instituto de Salud Carlos III. The CTS study would like to thank the CTS steering committee: Leslie Bernstein, Susan Neuhausen, James Lacey, Sophia Wang, Huiyan Ma, Yani Lu, and Jessica Clague DeHart at the Beckman Research Institute of the City of Hope, Dennis Deapen, Rich Pinder, Eunjung Lee, and Fred Schumacher at the University of Southern California, Pam Horn-Ross, Peggy Reynolds, Christina Clarke and David Nelson at the Cancer Prevention Institute of California, and Hoda Anton-Culver, Hannah Park, and Al Ziogas at the University of California Irvine. The CTS was initially supported by the California Breast Cancer Act of 1993 and the California Breast Cancer Research Fund (contract 97-10500) and is currently funded through the National Institutes of Health (R01 CA77398). Collection of cancer incidence data was supported by the California Department of Public Health as part of the statewide cancer reporting program mandated by California Health and Safety Code Section 103885. HAC receives support from the Lon V Smith Foundation (LVS39420). The ESTHER study would like to thank Hartwig Ziegler, Sonja Wolf, Volker Hermann. The ESTHER study was supported by a grant from the Baden Württemberg Ministry of Science, Research and Arts. Additional cases were recruited in the context of the VERDI study, which was supported by a grant from the German Cancer Aid (Deutsche Krebshilfe). The GENICA Network would like to thank Dr. Margarete Fischer-Bosch-Institute of Clinical Pharmacology, Stuttgart, and University of Tübingen, Germany; [HB, Wing-Yee Lo, Christina Justenhoven], Department of Internal Medicine, Evangelische Kliniken Bonn gGmbH, Johanniter Krankenhaus, Bonn, Germany [Yon-Dschun Ko, Christian Baisch], Institute of Pathology, University of Bonn, Germany [Hans-Peter Fischer], Molecular Genetics of Breast Cancer, Deutsches Krebsforschungszentrum (DKFZ), Heidelberg, Germany [UH], Institute for Prevention and Occupational Medicine of the German Social Accident Insurance, Institute of the Ruhr University Bochum (IPA), Bochum, Germany [Thomas Brüning, Beate Pesch, Sylvia Rabstein, Anne Lotz]; and Institute of Occupational Medicine and Maritime Medicine, University Medical Center Hamburg-Eppendorf, Germany [Volker Harth]. The GENICA was funded by the Federal Ministry of Education and Research (BMBF) Germany grants 01KW9975/5, 01KW9976/8, 01KW9977/0 and 01KW0114, the Robert Bosch Foundation, Stuttgart, Deutsches Krebsforschungszentrum

(DKFZ), Heidelberg, the Institute for Prevention and Occupational Medicine of the German Social Accident Insurance, Institute of the Ruhr University Bochum (IPA), Bochum, as well as the Department of Internal Medicine, Evangelische Kliniken Bonn gGmbH, Johanniter Krankenhaus, Bonn, Germany. The HEBCS would like to thank Carl Blomqvist, Kristiina Aittomäki, Taru A. Muranen and Irja Erkkilä. The HEBCS was financially supported by the Helsinki University Central Hospital Research Fund, Academy of Finland (266528), the Finnish Cancer Society, The Nordic Cancer Union and the Sigrid Juselius Foundation. The HERPACC was supported by MEXT Kakenhi (No. 170150181 and 26253041) from the Ministry of Education, Science, Sports, Culture and Technology of Japan, by a Grant-in-Aid for the Third Term Comprehensive 10-Year Strategy for Cancer Control from Ministry Health, Labour and Welfare of Japan, by Health and Labour Sciences Research Grants for Research on Applying Health Technology from Ministry Health, Labour and Welfare of Japan, by National Cancer Center Research and Development Fund, and "Practical Research for Innovative Cancer Control (15ck0106177h0001)" from Japan Agency for Medical Research and development, AMED, and Cancer Bio Bank Aichi. The HMBCS would like to thank Natalia Antonenkova, Peter Hillemanns, Hans Christiansen and Johann H. Karstens. The HMBCS was supported by a grant from the Friends of Hannover Medical School and by the Rudolf Bartling Foundation. Financial support for KARBAC was provided through the regional agreement on medical training and clinical research (ALF) between Stockholm County Council and Karolinska Institutet, the Swedish Cancer Society, The Gustav V Jubilee foundation and Bert von Kantzows foundation. The KBCP would like to thank Eija Myöhänen, Helena Kemiläinen. The KBCP was financially supported by the special Government Funding (EVO) of Kuopio University Hospital grants, Cancer Fund of North Savo, the Finnish Cancer Organizations, the Academy of Finland and by the strategic funding of the University of Eastern Finland. The kConFab/AOCS study would like to thank Heather Thorne, Eveline Niedermayr, all the kConFab research nurses and staff, the heads and staff of the Family Cancer Clinics, and the Clinical Follow Up Study (which has received funding from the NHMRC, the National Breast Cancer Foundation, Cancer Australia, and the National Institute of Health (USA)) for their contributions to this resource, and the many families who contribute to kConFab. kConFab is supported by a grant from the National Breast Cancer Foundation, and previously by the National Health and Medical Research Council (NHMRC), the Queensland Cancer Fund, the Cancer Councils of New South Wales, Victoria, Tasmania and South Australia, and the Cancer Foundation of Western Australia. The LAABC study would like to thank all the study participants and the entire data collection team, especially Annie Fung and June Yashiki. LAABC is supported by grants (1RB-0287, 3PB-0102, 5PB-0018, 10PB-0098) from the California Breast Cancer Research Program. Incident breast cancer cases were collected by the USC Cancer Surveillance Program (CSP), which is supported under subcontract by the California Department of Health. The CSP is also part of the National Cancer Institute's Division of Cancer Prevention and Control Surveillance, Epidemiology, and End Results Program, under contract number N01CN25403. The LMBC would like to thank Gilian Peuteman, Dominiek Smeets, Thomas Van Brussel and Kathleen Corthouts. LMBC is supported by the 'Stichting tegen Kanker' (232-2008 and 196-2010). Diether Lambrechts is supported by the FWO and the KULPFV/10/016-SymBioSysII. The MARIE study would like to thank Petra Seibold, Dieter Flesch-Janys, Judith Heinz, Nadia Obi, Alina Vrieling, Sabine Behrens, Ursula Eilber, Muhabbet Celik, Til Olchers, Stefan

Nickels. The MARIE study was supported by the Deutsche Krebshilfe e.V. [70-2892-BR I], the Hamburg Cancer Society, the German Cancer Research Center and the genotype work in part by the Federal Ministry of Education and Research (BMBF) Germany [01KH0402]. The MBCSG (Milan Breast Cancer Study Group) would like to thank Paolo Peterlongo of the Fondazione FIRC Institute of Molecular Oncology (IFOM); Siranoush Manoukian, Bernard Peissel, Daniela Zaffaroni and Jacopo Azzollini of the Fondazione IRCCS Istituto Nazionale dei Tumori (INT); Bernado Bonanni and Irene Feroce of the Istituto Europeo di Oncologia (IEO); and the personnel of the Cogentech Cancer Genetic Test Laboratory. MBCSG is supported by grants from the Italian Association for Cancer Research (AIRC) and by funds from the Italian citizens who allocated the 5/1000 share of their tax payment in support of the Fondazione IRCCS Istituto Nazionale Tumori, according to Italian laws (INT-Institutional strategic projects “5x1000”). The MCBCS was supported by the NIH grants CA192393, CA116167, CA176785 an NIH Specialized Program of Research Excellence (SPORE) in Breast Cancer [CA116201], and the Breast Cancer Research Foundation and a generous gift from the David F. and Margaret T. Grohne Family Foundation. MCCS cohort recruitment was funded by VicHealth and Cancer Council Victoria. The MCCS was further supported by Australian NHMRC grants 209057, 251553 and 504711 and by infrastructure provided by Cancer Council Victoria. Cases and their vital status were ascertained through the Victorian Cancer Registry (VCR) and the Australian Institute of Health and Welfare (AIHW), including the National Death Index and the Australian Cancer Database. The MEC was supported by NIH grants CA63464, CA54281, CA098758 and CA132839. The MTLGEBCS would like to thank Martine Tranchant (Cancer Genomics Laboratory, CHU de Québec Research Center), Marie-France Valois, Annie Turgeon and Lea Heguy (McGill University Health Center, Royal Victoria Hospital; McGill University) for DNA extraction, sample management and skillful technical assistance. J.S. is Chairholder of the Canada Research Chair in Oncogenetics. The work of MTLGEBCS was supported by the Quebec Breast Cancer Foundation, the Canadian Institutes of Health Research for the “CIHR Team in Familial Risks of Breast Cancer” program – grant # CRN- 87521 and the Ministry of Economic Development, Innovation and Export Trade – grant # PSR-SIIRI-701. The MYBRCA study would like to thank Phuah Sze Yee, Peter Kang, Kang In Nee, Kavitta Sivanandan, Shivaani Mariapun, Yoon Sook-Yee, Teh Yew Ching and Nur Aishah Mohd Taib for DNA Extraction and patient recruitment. MYBRCA is funded by research grants from the Malaysian Ministry of Science, Technology and Innovation (MOSTI), Malaysian Ministry of Higher Education (UM.C/HIR/MOHE/06) and Cancer Research Initiatives Foundation (CARIF). Additional controls were recruited by the Singapore Eye Research Institute, which was supported by a grant from the Biomedical Research Council (BMRC08/1/35/19/550), Singapore and the National medical Research Council, Singapore (NMRC/CG/SERI/2010). The NBCS has received funding from the K.G. Jebsen Centre for Breast Cancer Research; the Research Council of Norway grant 193387/V50 (to A-L Børresen-Dale and V.N. Kristensen) and grant 193387/H10 (to A-L Børresen-Dale and V.N. Kristensen), South Eastern Norway Health Authority (grant 39346 to A-L Børresen-Dale) and the Norwegian Cancer Society (to A-L Børresen-Dale and V.N. Kristensen). The NBCS Collaborators are: Dr. Kristine K. Sahlberg, PhD (Department of Research, Vestre Viken Hospital, Drammen, Norway and Department of Cancer Genetics, Institute for Cancer Research, Oslo University Hospital-Radiumhospitalet, Oslo, Norway), Dr. Lars Ottestad, MD (Department of

Cancer Genetics, Institute for Cancer Research, Oslo University Hospital-Radiumhospitalet, Oslo, Norway), Prof. Em. Rolf Kåresen, MD (Institute of Clinical Medicine, Faculty of Medicine, University of Oslo, Oslo, Norway and Department of Breast- and Endocrine Surgery, Division of Surgery, Cancer and Transplantation, Oslo University Hospital, Oslo, Norway), Dr. Anita Langerød, PhD (Department of Cancer Genetics, Institute for Cancer Research, Oslo University Hospital-Radiumhospitalet, Oslo, Norway), Dr. Ellen Schlichting, MD (Section for Breast- and Endocrine Surgery, Department of Cancer, Division of Surgery, Cancer and Transplantation Medicine, Oslo University Hospital, Oslo, Norway), Dr. Marit Muri Holmen, MD (Department of Radiology and Nuclear Medicine, Oslo University Hospital, Oslo, Norway), Prof. Toril Sauer, MD (Department of Pathology at Akershus University hospital, Lørenskog, Norway and Institute of Clinical Medicine, Faculty of Medicine, University of Oslo, Oslo, Norway), Dr. Vilde Haakensen, MD (Department of Cancer Genetics, Institute for Cancer Research, Oslo University Hospital-Radiumhospitalet, Oslo, Norway), Dr. Olav Engebråten, MD (Department of Tumor Biology, Institute for Cancer Research, Oslo University Hospital, Oslo, Norway, Department of Oncology, Division of Surgery and Cancer and Transplantation Medicine, Oslo University Hospital, Oslo, Norway and Institute for Clinical Medicine, Faculty of Medicine, University of Oslo, Oslo, Norway), Prof. Bjørn Naume, MD (Department of Oncology, Division of Surgery and Cancer and Transplantation Medicine, Oslo University Hospital-Radiumhospitalet, Oslo, Norway), Dr. Cecile E. Kiserud, MD (National Advisory Unit on Late Effects after Cancer Treatment, Department of Oncology, Oslo University Hospital, Oslo, Norway and Department of Oncology, Oslo University Hospital, Oslo, Norway), Dr. Kristin V. Reinertsen, MD (National Advisory Unit on Late Effects after Cancer Treatment, Department of Oncology, Oslo University Hospital, Oslo, Norway and Department of Oncology, Oslo University Hospital, Oslo, Norway), Assoc. Prof. Åslaug Helland, MD (Department of Cancer Genetics, Institute for Cancer Research and Department of Oncology, Oslo University Hospital-Radiumhospitalet, Oslo, Norway), Dr. Margit Riis, MD (Dept of Breast- and Endocrine Surgery, Oslo University Hospital, Ullevål, Oslo, Norway), Dr. Ida Bukholm, MD (Department of Breast-Endocrine Surgery, Akershus University Hospital, Oslo, Norway and Department of Oncology, Division of Cancer Medicine, Surgery and Transplantation, Oslo University Hospital, Oslo, Norway), Prof. Per Eystein Lønning, MD (Section of Oncology, Institute of Medicine, University of Bergen and Department of Oncology, Haukeland University Hospital, Bergen, Norway), OSBREAC (Oslo Breast Cancer Research Consortium; Oslo University Hospital) and Grethe I. Grenaker Alnæs, M.Sc. (Department of Cancer Genetics, Institute for Cancer Research, Oslo University Hospital-Radiumhospitalet, Oslo, Norway).The NBHS_TN study would like to thank participants and research staff for their contributions and commitment to this study. The NBHS was supported by NIH grant R01CA100374. Biological sample preparation was conducted the Survey and Biospecimen Shared Resource, which is supported by P30 CA68485. The OBCS study would like to thank Meeri Otsukka, Kari Mononen, Mervi Grip, Saila Kauppila. The OBCS was supported by research grants from the Finnish Cancer Foundation, the Academy of Finland (grant number 250083, 122715 and Center of Excellence grant number 251314), the Finnish Cancer Foundation, the Sigrid Juselius Foundation, the University of Oulu, the University of Oulu Support Foundation and the special Governmental EVO funds for Oulu University Hospital -based research activities. The OFBCR study would like to thank Teresa Selander, Nayana Weerasooriya. The Ontario Familial Breast Cancer Registry

(OFBCR) was supported by grant UM1 CA164920 from the National Cancer Institute (USA). The content of this manuscript does not necessarily reflect the views or policies of the National Cancer Institute or any of the collaborating centers in the Breast Cancer Family Registry (BCFR), nor does mention of trade names, commercial products, or organizations imply endorsement by the USA Government or the BCFR. The ORIGO study would like to thank E. Krol-Warmerdam, and J. Blom for patient accrual, administering questionnaires, and managing clinical information. The LUMC survival data were retrieved from the Leiden hospital-based cancer registry system (ONCDOC) with the help of Dr. J. Molenaar. The ORIGO study was supported by the Dutch Cancer Society (RUL 1997-1505) and the Biobanking and Biomolecular Resources Research Infrastructure (BBMRI-NL CP16). The PBCS study would like to thank Louise Brinton, Mark Sherman, Neonila Szeszenia-Dabrowska, Beata Peplonska, Witold Zatonski, Pei Chao, Michael Stagner. The PBCS was funded by Intramural Research Funds of the National Cancer Institute, Department of Health and Human Services, USA. The pKARMA study would like to thank The Swedish Medical Research Counsel. The pKARMA study was supported by Märit and Hans Rausings Initiative Against Breast Cancer. The RBCS study would like to thank Petra Bos, Jannet Blom, Ellen Crepin, Elisabeth Huijskens, Annette Heemskerk, the Erasmus MC Family Cancer Clinic. The RBCS was funded by the Dutch Cancer Society (DDHK 2004-3124, DDHK 2009- 4318). The SASBAC would like to thank The Swedish Medical Research Counsel. The SASBAC study was supported by funding from the Agency for Science, Technology and Research of Singapore (A*STAR), the US National Institute of Health (NIH) and the Susan G. Komen Breast Cancer Foundation. The SBCGS would like to thank participants and research staff for their contributions and commitment to this study. The SBCGS was supported primarily by NIH grants R01CA64277, R01CA148667, and R37CA70867. Biological sample preparation was conducted the Survey and Biospecimen Shared Resource, which is supported by P30 CA68485. The scientific development and funding of this project were, in part, supported by the Genetic Associations and Mechanisms in Oncology (GAME-ON) Network U19 CA148065. The SBCS would like to thank Sue Higham, Helen Cramp, Sabapathy Balasubramanian, Ian Borck and Dan Connley. The SBCS was supported by Yorkshire Cancer Research S295, S299, S305PA. The SEARCH study would like to thank The SEARCH and EPIC teams. SEARCH is funded by a programme grant from Cancer Research UK [C490/A10124] and supported by the UK National Institute for Health Research Biomedical Research Centre at the University of Cambridge. SEBCS was supported by the BRL (Basic Research Laboratory) program through the National Research Foundation of Korea funded by the Ministry of Education, Science and Technology (2012-0000347). The SGBCC study would like to thank the participants and research coordinator Kimberley Chua. SGBCC is funded by the NUS start-up Grant, National University Cancer Institute Singapore (NCIS) Centre Grant and the NMRC Clinician Scientist Award. Additional controls were recruited by the Singapore Consortium of Cohort Studies-Multi-ethnic cohort (SCCS-MEC), which was funded by the Biomedical Research Council, grant number: 05/1/21/19/425. The SKKDKFZS study thanks all study participants, clinicians, family doctors, researchers and technicians for their contributions and commitment to this study. SKKDKFZS is supported by the DKFZ. The SZBCS was supported by Grant PBZ_KBN_122/P05/2004. The TBCS was funded by The National Cancer Institute Thailand. The TNBCC was supported by: a Specialized Program of Research Excellence (SPORE) in Breast Cancer (CA116201), a

grant from the Breast Cancer Research Foundation, a generous gift from the David F. and Margaret T. Grohne Family Foundation and the Ting Tsung and Wei Fong Chao Foundation, the Stefanie Spielman Breast Cancer fund and the OSU Comprehensive Cancer Center, DBBR (a CCSG Share Resource by National Institutes of Health Grant P30 CA016056), the Hellenic Cooperative Oncology Group research grant (HR R_BG/04) and the Greek General Secretary for Research and Technology (GSRT) Program, Research Excellence II, the European Union (European Social Fund – ESF), and Greek national funds through the Operational Program "Education and Lifelong Learning" of the National Strategic Reference Framework (NSRF) - ARISTEIA. The TWBCS is supported by the Taiwan Biobank project of the Institute of Biomedical Sciences, Academia Sinica, Taiwan. The UKBGS study would like to thank Breast Cancer Now and the Institute of Cancer Research (ICR) for support and funding of the Breakthrough Generations Study, and the study participants, study staff, and the doctors, nurses and other health care providers and health information sources who have contributed to the study. We acknowledge NHS funding to the Royal Marsden/ICR NIHR Biomedical Research Centre. The UKBGS is funded by Breast Cancer Now and the Institute of Cancer Research (ICR), London. ICR acknowledges NHS funding to the NIHR Biomedical Research Centre.
